# Genetic and immunologic features of recurrent stage I lung adenocarcinoma

**DOI:** 10.1038/s41598-021-02946-0

**Published:** 2021-12-08

**Authors:** Johannes R. Kratz, Jack Z. Li, Jessica Tsui, Jen C. Lee, Vivianne W. Ding, Arjun A. Rao, Michael J. Mann, Vincent Chan, Alexis J. Combes, Matthew F. Krummel, David M. Jablons

**Affiliations:** 1 Department of Surgery, Division of Cardiothoracic Surgery, University of California San Francisco, 500 Parnassus Ave, MUW-405, San Francisco, CA 94143 USA; 2 Thoracic Oncology Laboratory, University of California San Francisco, San Francisco, CA 94143 USA; 3 Helen Diller Family Comprehensive Cancer Center, University of California San Francisco, San Francisco, CA 94143 USA; 4 ImmunoX Initiative, University of California San Francisco, San Francisco, CA 94143 USA; 5 Department of Pathology, University of California San Francisco, San Francisco, CA 94143 USA; 6 UCSF CoLabs, University of California San Francisco, San Francisco, CA 94143 USA; 7 Department of Microbiology and Immunology, University of California San Francisco, San Francisco, CA 94143 USA

**Keywords:** Non-small-cell lung cancer, Tumour immunology

## Abstract

Although surgery for early-stage lung cancer offers the best chance of cure, recurrence still occurs between 30 and 50% of the time. Why patients frequently recur after complete resection of early-stage lung cancer remains unclear. Using a large cohort of stage I lung adenocarcinoma patients, distinct genetic, genomic, epigenetic, and immunologic profiles of recurrent tumors were analyzed using a novel recurrence classifier. To characterize the tumor immune microenvironment of recurrent stage I tumors, unique tumor-infiltrating immune population markers were identified using single cell RNA-seq on a separate cohort of patients undergoing stage I lung adenocarcinoma resection and applied to a large study cohort using digital cytometry. Recurrent stage I lung adenocarcinomas demonstrated higher mutation and lower methylation burden than non-recurrent tumors, as well as widespread activation of known cancer and cell cycle pathways. Simultaneously, recurrent tumors displayed downregulation of immune response pathways including antigen presentation and Th1/Th2 activation. Recurrent tumors were depleted in adaptive immune populations, and depletion of adaptive immune populations and low cytolytic activity were prognostic of stage I recurrence. Genomic instability and impaired adaptive immune responses are key features of stage I lung adenocarcinoma immunosurveillance escape and recurrence after surgery.

## Introduction

Over 1.8 million people are diagnosed with lung cancer worldwide annually, resulting in 1.6 million deaths each year^1^. Although early detection has been shown to improve survival in other cancers, early detection of lung cancer is not enough. Even after complete surgical resection, early-stage lung cancers recur up to 30–50% of the time within 5 years^2^, causing a staggering number of deaths worldwide. Despite these overwhelming recurrence rates, the current standard of care for most patients who undergo surgery for early-stage lung cancer is “observation”. Chemotherapy after surgery does not improve survival for the majority of these patients^3^.

To predict which patients may recur after surgery, clinicians have traditionally grouped patients using the TNM (Tumor, Node, Metastasis) staging system^4^. Multiple groups, however, have identified molecular signatures that more accurately predict recurrence and mortality beyond conventional TNM staging^5–13^. These signatures are based on a variety of genetic, genomic, and epigenetic biomarkers prognostic of recurrence. Despite an improved ability to predict who may recur based on these biomarkers, the biology of why these patients recur after surgery is less well understood. While multiple studies have described the genetic, genomic, and epigenetic alterations in tumor vs. normal tissue^14,15^, only a handful of groups have investigated the biology of early-stage lung cancer recurrence^16,17^. These studies have primarily focused on mutation profiles associated with recurrence by utilizing targeted NGS panels of known cancer mutations^16,17^. A more comprehensive account of the genomic and epigenetic features of recurrence, however, has not been described.

The relationship between the tumor immune microenvironment and early-stage lung cancer recurrence after surgery also remains poorly understood. Advances in cancer immunotherapy have highlighted the importance of the immune system in keeping tumor progression and metastasis in check. Several cancer-immune landscapes have been described: 1. inflamed or “hot” tumors, infiltrated by exhausted and ineffective immune cells, 2. immune-desert or “cold” tumors, which lack appropriate T-cell activation and priming, and 3. immune-excluded tumors, in which vascular or stromal barriers prevent immune infiltration^18^. Precisely defining the cancer-immune landscape of recurrent early-stage lung cancer has important clinical implications. Multiple studies, for example, have demonstrated that checkpoint inhibitors are primarily effective in “hot” tumor landscapes with a large number of infiltrating but dormant T-cells^18^. In fact, the presence of alternative “cold” and “excluded” tumor landscapes may explain why a durable clinical response to checkpoint inhibitors is seen in only approximately 20% of patients^18^. To address this, several groups have assessed the prognostic significance of tumor infiltrating leukocytes in lung adenocarcinomas using immunohistochemistry with a pre-defined set of immune cell surface markers and cytokines^19,20^ or by using computational immune profiling techniques^21,22^. These groups, however, have only assessed small focused panels of immune cell types^19,20^ or used pre-defined sets of immune cell markers not derived from tumor infiltrating leukocytes to perform computational profiling^21,22^. More comprehensive and specific descriptions of the tumor immune microenvironment characteristic of early recurrence after surgery are still needed to guide our current immunotherapy treatments and reveal novel immunotherapy targets.

This study investigates the molecular and immunologic features of early recurrence in stage I lung adenocarcinoma using a large cohort of patients with complete genomic, genetic, epigenetic, and clinical profiles. Unique early-stage lung adenocarcinoma immune population profiles developed using single cell RNA-seq profiling of freshly resected stage I lung adenocarcinomas allows for comprehensive characterization of immune populations in this cohort. Our analysis reveals distinct genetic, genomic, epigenetic, and immunologic differences between recurrent and non-recurrent early-stage lung adenocarcinomas and identifies several novel biomarkers and therapeutic targets associated with early recurrence.

## Results

### Recurrence score

**Figure 1:**
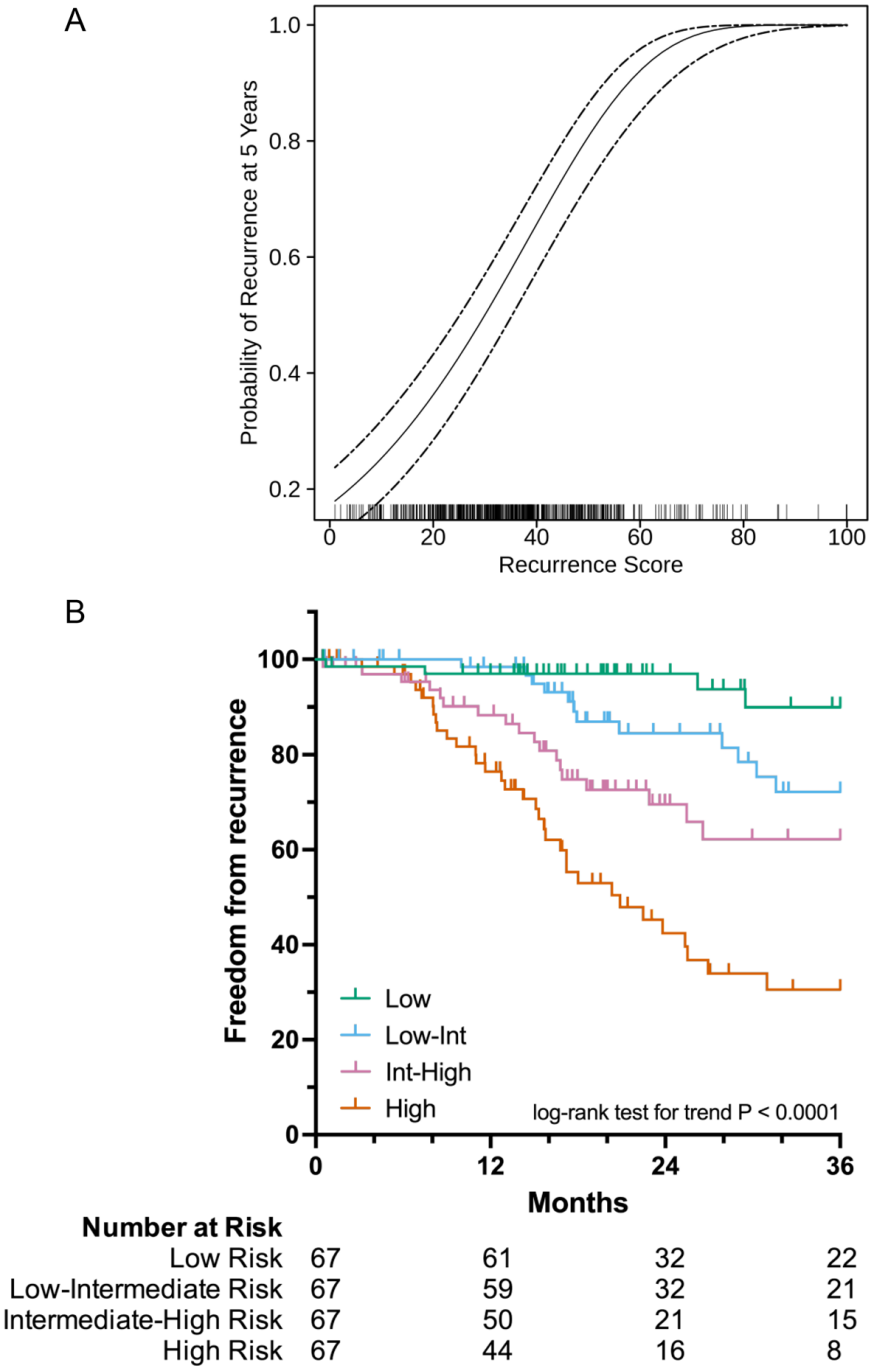
Recurrence score is prognostic of recurrence in the TCGA lung adenocarcinoma cohort. Recurrence score is directly proportional to probability of recurrence at 5 years within the entire TCGA lung adenocarcinoma cohort (**A**) and is prognostic of recurrence when stage I patients are divided into cohorts by recurrence score quartile (**B**).

In order to incorporate time-to-recurrence data, a score that predicts the risk of recurrence for each patient in the TCGA lung adenocarcinoma was developed. This recurrence score relates each patient’s gene expression profile to both recurrence as well as time-to-recurrence. Elastic net penalization using cross-validation yielded a parsimonious set of genes associated with recurrence in 500 patients with stage I-IV lung adenocarcinoma in the TCGA cohort (Supplementary Table S1). Weighting and combining the expression of these risk classifier genes by coefficients assigned by elastic net penalization (Supplementary Table S1) resulted in a recurrence score for each patient in the TCGA lung adenocarcinoma cohort. Higher recurrence scores were directly proportional to the probability of recurrence within 5 years of diagnosis (Fig. 1A). Grouping patients into cohorts by recurrence score quartile in the stage I-IV TCGA lung adenocarcinoma cohort resulted in significantly different recurrence rates by Kaplan–Meier analysis (5-year Freedom From Recurrence (FFR) of 68.9%, 39.5%, 34.8%, 25.0% for low-, low-intermediate, intermediate-high, and high-risk groups respectively, *P* < 0.0001). The TCGA lung adenocarcinoma cohort includes patients who presented with nodal and distant organ spread. In order to study the biology of recurrence in patients not known to have disease spread, we limited further analysis to stage I patients only. Grouping stage I lung adenocarcinoma patients into cohorts by recurrence score quartile identified a high-risk cohort with 57.6% incidence of recurrence within 2 years of surgical resection as opposed 3.1% in the low-risk cohort (Fig. 1B, 2-year FFR 96.9%, 84.5%, 69.6%, 42.4% for low-, low-intermediate, intermediate-high, and high-risk groups respectively, *P* < 0.0001). By univariate analysis, recurrence risk category and stage were statistically significant predictors of recurrence (Table 1). Adjusting for age, smoking history, and stage, only recurrence risk category (HR 2.09, 95% CI 1.54–2.84, *P* < 0.0001) and female sex (HR 0.67, 95% CI 0.49–0.92, *P* = 0.012) were significant predictors of recurrence by multivariate analysis (Table 1).

**Table 1:** Cox proportional hazards models for 5-year freedom from recurrence.

	Univariate analysis			Multivariate analysis		
	HR	95% CI	Wald test *P* value	HR	95% CI	Wald test *P* value
Risk category*	**2.01**	**1.59–2.52**	** < 0.001**	**2.09**	**1.54–2.84**	** < 0.001**
Age > 65	0.92	0.58–1.46	0.731	1.01	0.58–1.75	0.976
Smoking history^§^	1.20	0.86–1.68	0.292	0.99	0.70–1.38	0.937
Female sex	0.97	0.61–1.55	0.914	**0.67**	**0.49–0.92**	**0.012**
Stage**	**1.61**	**1.01–2.56**	**0.045**	1.28	0.72–2.29	0.402

Univariate and multivariate cox proportional hazards modeling of the association between recurrence, recurrence risk category, and clinicopathologic factors is shown.

Significant values are in bold.

*Compared to low risk category.

^§^< 5, 6–20, 21–40, > 40 pack-years.

**Stage IA v. IB.

Conventionally, TNM stage is used to predict risk of recurrence. In order to compare risk predictions using our risk categorization method vs. TNM stage in this cohort, we performed time-dependent area under the receiver operating characteristic curve (AUROC) analysis (Supplementary Fig. S1A). The recurrence score better predicted risk of recurrence versus stage alone, increasing the time-dependent AUROC to 0.714 from 0.584 (*P* < 0.0001).

### Genetic alterations

**Figure 2:**
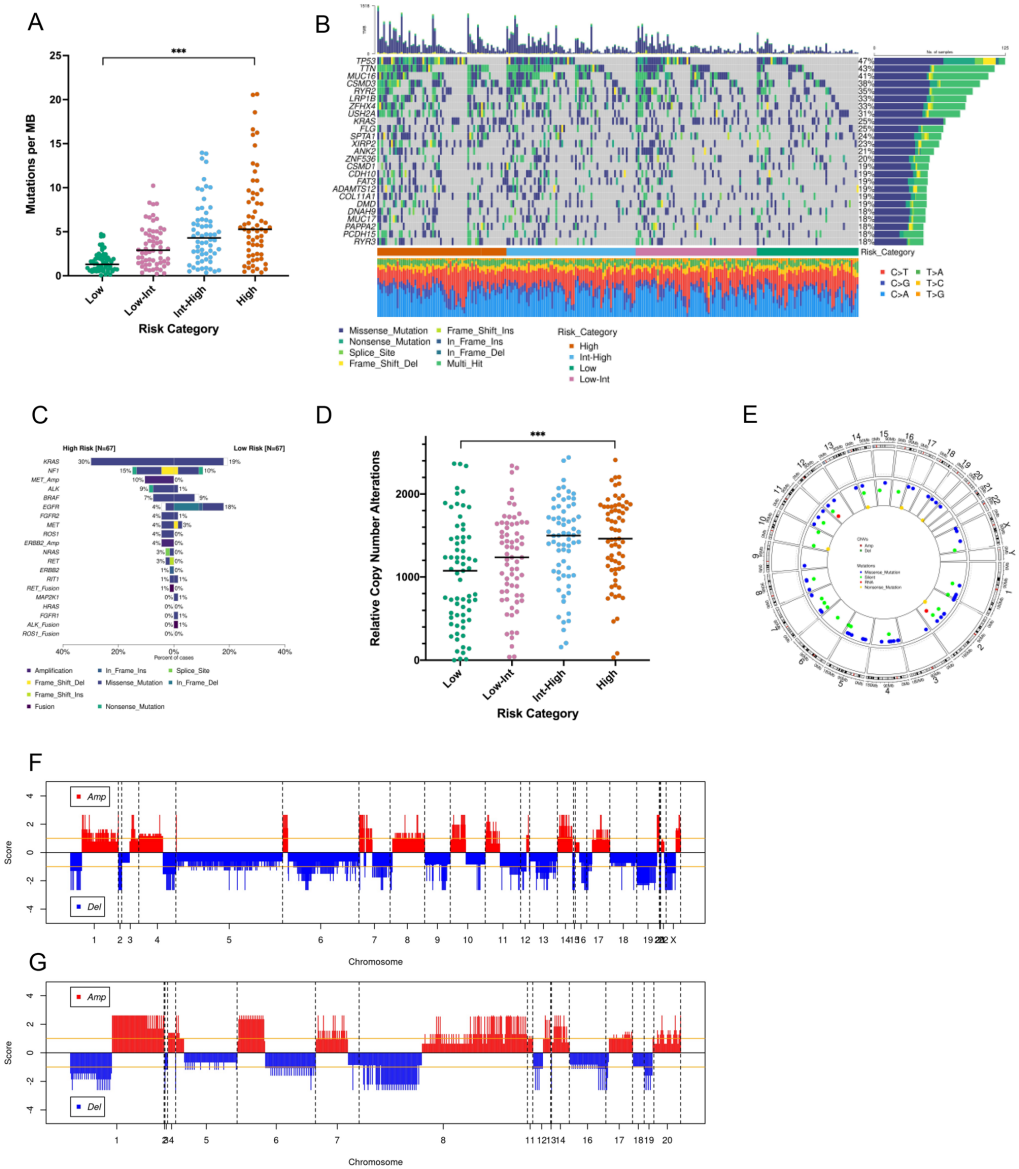
Genetic alterations in recurrent lung adenocarcinomas. Increasing risk of recurrence is associated with increasing tumor mutation burden (**A**). An oncoplot of the most frequently mutated genes in the stage I lung adenocarcinoma cohort is shown in (**B**). Canonical driver mutations are present in stage I tumors but do not occur more frequently in recurrent tumors (**C**). Copy number alterations occur more frequently in tumors with increasing risk of recurrence (**D**). Although copy number alterations and mutations are evenly distributed through the genome in recurrence high-risk tumors (**E**), high-risk (**F**) versus low-risk (**G**) tumors show different genome-wide copy number alteration patterns. ***P < 0.0001.

The median tumor mutation burden observed in the TCGA stage I lung adenocarcinoma cohort was 3.52 mutations per megabase. When assessing tumor mutation burden by recurrence risk category in this cohort, a positive association between tumor mutation burden and risk of recurrence was observed (median tumor mutations per MB of 1.62, 3.15, 4.66, and 5.48 in recurrent low-, low-intermediate, intermediate-high, and high-risk tumor phenotypes respectively [*P* < 0.0001], Fig. 2A). *TP53* was most frequently mutated in the stage I cohort; 47% of samples had a *TP53* mutation (Fig. 2B). Other frequently mutated genes included *TTN*, *MUC16*, *CSMD3*, *RYR2*, *LRP1B*, *ZFHX4*, *USH2A*, *KRAS*, and *FLG* (Fig. 2B). 23 genes were mutated significantly more frequently in high- vs. low-risk stage I tumors, including *TP53*, *TTN*, *SLC8A1*, *AHNAK*, *KCNU1*, *COLA5A2*, *COL22A1*, *PKHD1L1*, *SMARCA4*, and *ZFHX4* (Table 2). Canonical lung cancer driver mutations^23^ were also assessed. Although *KRAS* mutations were observed more frequently in high-risk tumors (30% vs. 19% in low-risk tumors, adjusted *P* = 0.31) and *EGFR* mutations were observed more frequently in low-risk tumors (18% vs. 4% in high-risk tumors, adjusted *P* = 0.13), these differences were not statistically significant. In fact, no significant differences in canonical lung cancer driver mutations were observed between low- and high-risk tumors (Fig. 2C). Similar to tumor mutation burden, copy number alterations were significantly increased in recurrence high- vs. low-risk tumors (median relative CNA of 1075 vs. 1462 in low- vs. high-risk categories respectively [*P* < 0.0001], Fig. 2D). Although copy number alterations and mutations did not show any predilection for a particular chromosome in high-risk tumors (Fig. 2E), different patterns of copy number alterations were observed between low- and high-risk tumors across the genome (Fig. 2F and G).

**Table 2:** Genes more frequently mutated in recurrence high- versus low-risk stage I lung adenocarcinomas.

Hugo symbol	# High risk mutated samples	# Low risk mutated samples	*P*-value	Odds ratio	95% upper CI	95% lower CI	Adj *P*-value
TP53	46	16	0	6.866	16.123	3.059	0
TTN	39	12	0	6.286	15.403	2.722	0.001
SLC8A1	14	0	0	Inf	Inf	3.92	0.021
AHNAK	13	0	0	Inf	Inf	3.54	0.028
KCNU1	13	0	0	Inf	Inf	3.54	0.028
COL5A2	15	1	0	18.728	810.111	2.713	0.031
COL22A1	12	0	0	Inf	Inf	3.174	0.031
PKHD1L1	12	0	0	Inf	Inf	3.174	0.031
SMARCA4	12	0	0	Inf	Inf	3.174	0.031
ZFHX4	31	11	0	4.334	10.836	1.844	0.031
CSMD3	37	16	0	3.889	8.86	1.769	0.031
ADAMTS12	22	5	0	5.984	21.784	2.01	0.031
NRXN1	22	5	0	5.984	21.784	2.01	0.031
CACNA1E	16	2	0.001	10.046	94.084	2.204	0.031
FAT4	16	2	0.001	10.046	94.084	2.204	0.031
XIRP2	26	8	0.001	4.623	13.044	1.81	0.031
CDH18	11	0	0.001	Inf	Inf	2.82	0.031
CTNND2	11	0	0.001	Inf	Inf	2.82	0.031
DMXL1	11	0	0.001	Inf	Inf	2.82	0.031
MAGI2	11	0	0.001	Inf	Inf	2.82	0.031
SLC39A12	11	0	0.001	Inf	Inf	2.82	0.031
MUC16	30	11	0.001	4.083	10.214	1.734	0.031
TCHH	13	1	0.001	15.644	682.961	2.218	0.049

Twenty-three genes significantly more frequently mutated in recurrence high- versus low-risk tumors are shown.

### Transcriptome analysis

**Figure 3:**
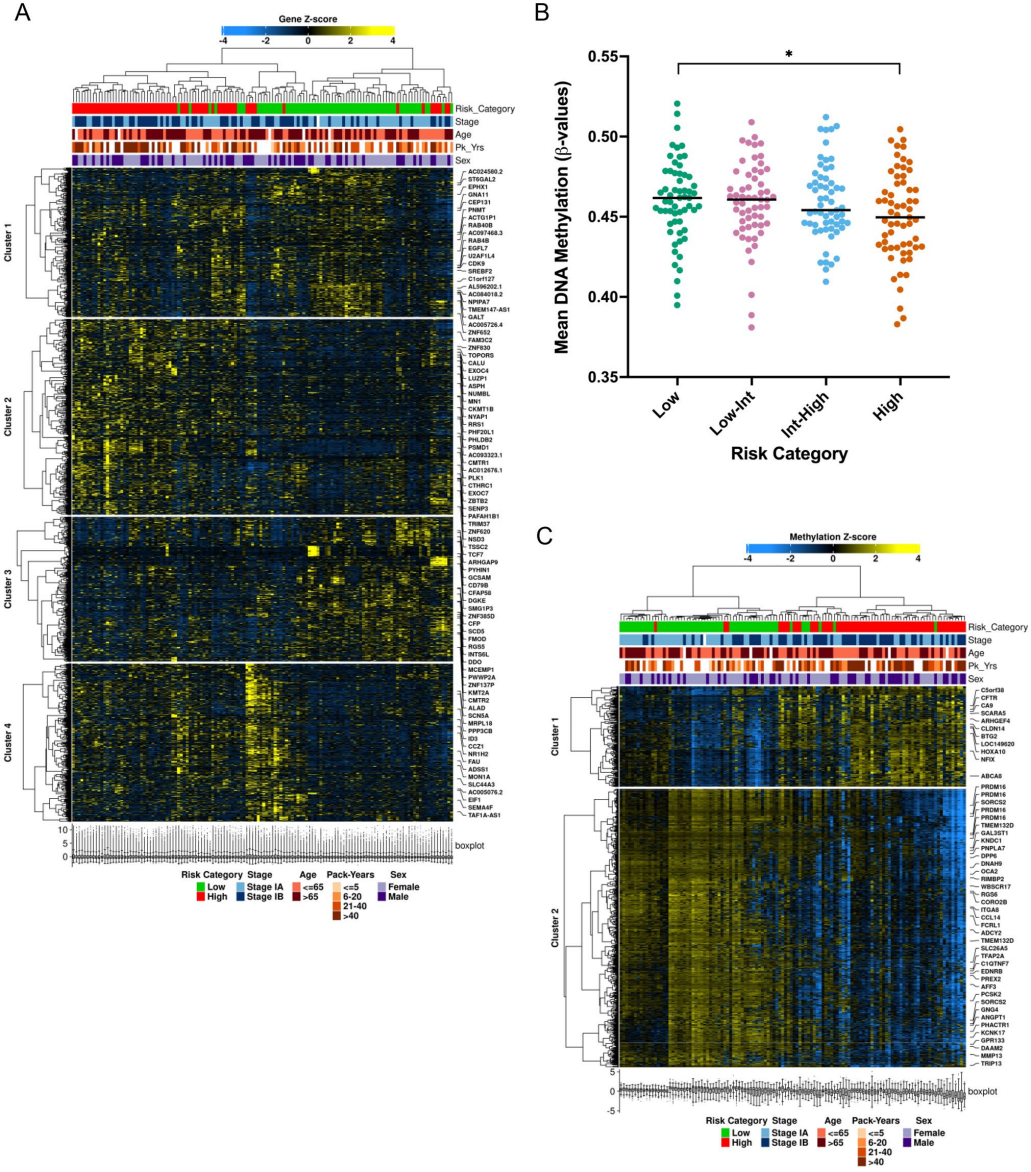
Transcriptomic analysis demonstrates distinct genomic and immunologic features of recurrent lung adenocarcinomas. Heatmap clustering of differentially regulated genes results in clear separation between high- and low-risk tumors across four different gene clusters (**A**). Differential methylation patterns are also associated with recurrent tumors; decreasing DNA methylation is observed in tumors with increasing risk of recurrence (**B**) while heatmap clustering of differentially methylated genes results in clear separation between high- and low-risk tumors across two different gene clusters (**C**). *P < 0.05.

Comparing transcriptomes of recurrence high vs. low-risk tumors, 10,398 genes were differentially expressed (adjusted *P* value < 0.05). Filtering these genes by an absolute log fold-change > 0.6 and adjusted *P* value < 0.0001, we identified a total of 4150 top differentially regulated genes (2544 downregulated and 1606 upregulated genes, Supplementary Fig. S2A). Heatmap clustering of these top differentially regulated genes resulted in clear separation between high- and low-risk tumors across four different gene clusters (*P* < 0.0001, Fig. 3A). Stage (*P* = 0.0003) and smoking history (*P* = 0.0163) also showed separation by heatmap clustering. In contrast, age and sex were not significantly correlated with these gene clusters (Fig. 3A).

**Figure 4:**
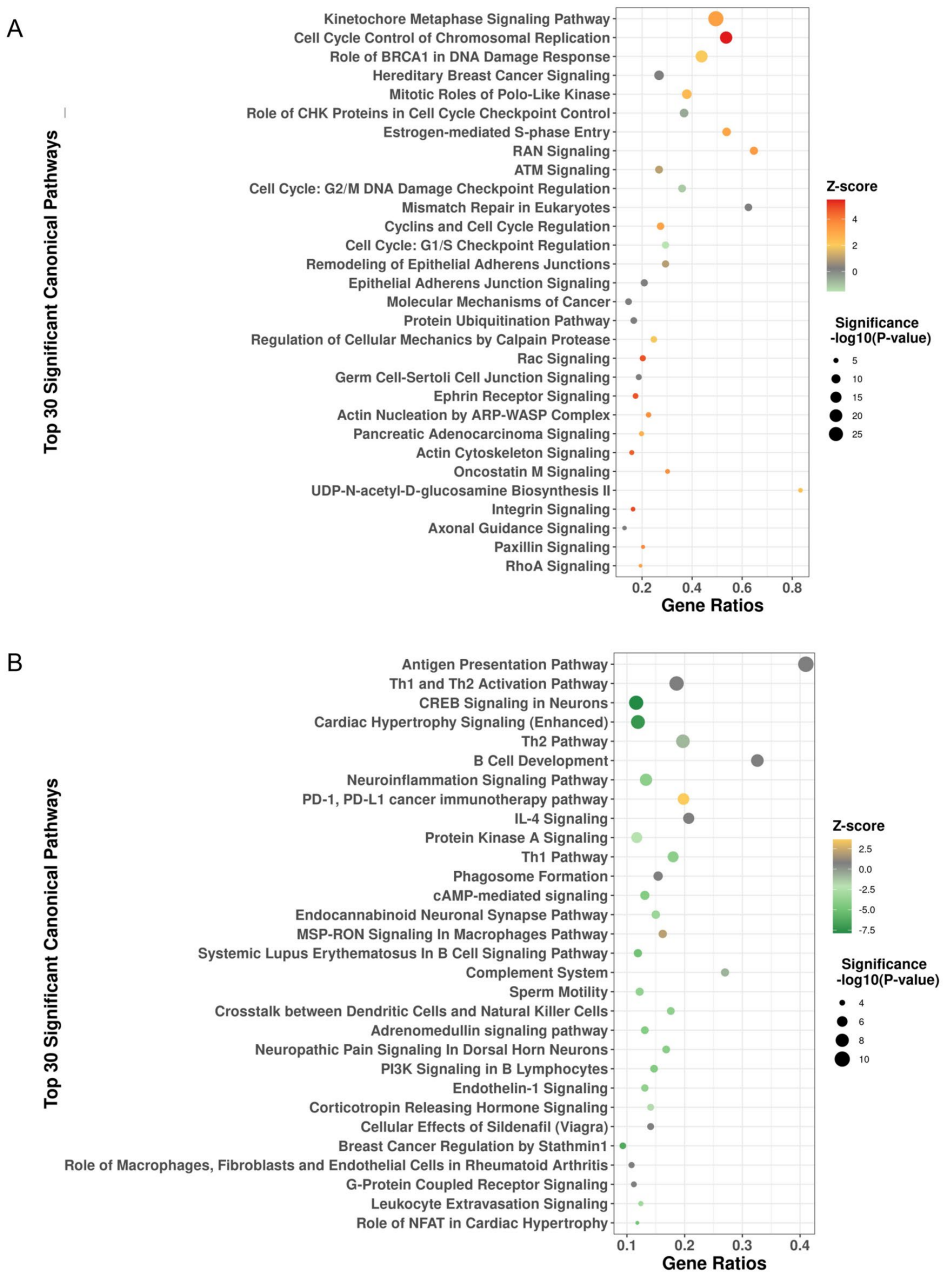
Pathway analysis for differential gene expression heatmap clusters 2 and 3. The top 30 pathways involved in the differential expression of genes in heatmap cluster 2 and 3 are shown in (A) and (B) respectively. Ratio on the x-axis refers to the number of differentially regulated genes in the dataset relative to the total number of pathway genes. The size of each dot represents significance (− log10(*P*-value)); the color of each dot represents the Z-score. A positive Z-score indicates that the observed gene activity positively correlates with predicted pathway member up/down regulation patterns; a negative Z-score indicates anti-correlation.

Recurrence high-risk tumors were associated with overexpression of genes in cluster 2 (Fig. 3A). Pathway analysis of genes in this cluster revealed activation of numerous cancer-related pathways including kinetochore metaphase signaling, cell cycle control of chromosomal replication, role of BRCA1 in DNA damage response, mitotic roles of polo-like kinase, hereditary breast cancer signaling, role of CHK proteins in cell cycle checkpoint control, and estrogen-mediated S-phase entry pathways (Fig. 4A, Supplementary Table S2).

Simultaneously, recurrence high-risk tumors were associated with downregulation of genes in cluster 3 (Fig. 3A). Genes in cluster 3 were significantly associated with immune response pathways including antigen presentation, Th1 and Th2 activation, B cell development, neuroinflammation signaling, PD-1 and PD-L1 cancer immunotherapy, and IL-4 signaling pathways (Fig. 4B, Supplementary Table S3). 16 out of 39 (41%) genes involved in antigen presentation were significantly downregulated in high-risk tumors (*P* < 0.0001) including *CIITA,* a master control regulator of class II MHC transcription (2.3 fold), numerous MHC class I and II genes including *HLA-E* (1.6 fold), *HLA-DMA* (2.7 fold), *HLA-DOA* (3.0 fold), *HLA-DPA1* (2.5 fold), *HLA-DQB2* (4.3 fold), *HLA-DRB5* (4.0 fold), and *CD74*, a class II MHC chaperone molecule (2.5 fold). Simultaneously, 32 out of 172 (18.6%) genes involved in Th1 and Th2 activation were significantly downregulated in high-risk tumors including the class II MHC genes above, *CCR4* (2.7 fold), *CD40LG* (3.7 fold), *IL24* (1.8 fold), *IL33* (3.1 fold), *IL12B* (2.8 fold), *IL6R* (2.4 fold), *TGF-β* receptors 2 and 3 (1.5 and 2.3 fold respectively), *PTDGR2* (3.4 fold), *NFATC1* (1.5 fold), *PIK3R1* (1.4 fold), and *PRKCQ* (2.4 fold).

Topology-based enrichment analysis is a third-generation pathway analysis technique that better relates gene expression patterns to biological pathways by taking the role, hierarchy, and expression direction of pathway genes into account^24^. Using topology-based enrichment analysis, 42 pathways are significantly perturbed in high- vs. low-risk tumors (pPERT < 0.05 and pGFdr < 0.05, Supplementary Table S4). Numerous cancer-related pathways are activated in recurrence high-risk tumors including central carbon metabolism in cancer, toll-like receptor signaling, and hedgehog signaling pathways. Simultaneously, cellular senescence, Ras signaling, and PPAR signaling pathways are inhibited in high-risk tumors. Multiple immune-related pathways are also perturbed in high-risk tumors. Human cytomegalovirus infection, bacterial invasion of epithelial cells, and complement and coagulation cascades pathways are activated. In contrast, cytokine-cytokine receptor interaction, human T-cell leukemia virus 1 infection, systemic lupus erythematosus, and intestinal immune network for IgA production pathways are inhibited in high-risk tumors (Supplementary Table S4).

### Epigenetic analysis

To investigate epigenetic features associated with recurrence, methylation differences between high- and low-risk tumors were analyzed. Increasing recurrence risk was associated with decreasing DNA methylation across risk categories (median β-value of 0.462, 0.461, 0.454, and 0.450 for low-, low-intermediate-, intermediate-high-, and high-risk categories respectively [*P* = 0.042 between low- and high-risk categories], Fig. 3B). A total of 1,514 genes were differentially methylated between high- and low-risk tumors (absolute log fold-change > 0.4 and adjusted *P* value < 0.05). Genes were more than three times as likely to be hypo- rather than hypermethylated in the high-risk cohort (1173 hypomethylated genes vs. 341 hypermethylated genes, Supplementary Fig. S2B). The top 10 differentially methylated genes were *FOLR1*, *LEAP2*, *CRTAM*, *MIR1287*, *KIAA0513*, *GZMB*, *HTRA1*, *TMEM11*, *FAM49A*, and *SORCS3* (Supplementary Fig. S2C). Heatmap clustering of differentially methylated genes resulted in clear separation between high- and low-risk tumors across two different gene clusters (*P* < 0.0001, Fig. 3C). Stage (*P* = 0.0004) and smoking history (*P* = 0.0068) also showed separation by heatmap clustering. In contrast, age and sex were not significantly correlated with these differentially methylated gene clusters (Fig. 3C). Pathway analysis of differentially methylated genes identified significant involvement of immune-related pathways including antigen binding, processing, and presentation pathways by Gene Ontology (GO) analysis and allograft rejection by Molecular Signatures Database analysis (Supplementary Table S5).

Differential methylation analysis was also performed on a chromosome level, revealing 3461 differentially methylated regions (adjusted *P* < 0.0001). As differential methylation pathway analysis identified antigen presentation pathways as significantly involved in high-risk tumors, chromosome 6 was examined for differentially methylated regions. High-risk tumors demonstrated heavy methylation in a specific region of chromosome 6 (bp 32,410,873 to 33,041,697) containing class II MHC genes (Supplementary Fig. S3A).

Integrated transcriptome and methylation analysis demonstrated a total of 295 genes that were both hypomethylated and upregulated in high-risk patients including *GPR115*, *KRT6A*, *KRT6B*, *KRT16*, *PPAPDC1A*, *PADI1*, *HMGA2*, *SLC2A1*, *CCL7* and *CCL11* (Supplementary Fig. S3B). 771 genes were both hypermethylated and downregulated in high-risk patients including *FOLR1*, *NR0B2*, *AGER*, *GPR116*, *CLDN18*, *CYP4B1*, *PGC*, *KIAA0408*, and *PIGR* (Supplementary Fig. S3B). Integrated analysis using enhancer linking also identified multiple differentially methylated transcription factor promoter binding sites. The transcription factors CENPA, MYBL2, FOXM1, ARNTL2, SHOX2, GBX2, E2F2, E2F7, HMGA1, and SP6 were found to be upregulated and bind hypomethylated promoter sites (Supplementary Fig. S4A), whereas IRX5, NKX2-1, HLF, ZBTB18, ATOH8, PRDM16, IRX3, IRX2, ZNF491, and NR3C2 were found to be downregulated and bind hypermethylated promoter sites in high- vs. low-risk tumors (Supplementary Fig. S4B).

### Tumor immune microenvironment

**Figure 5:**
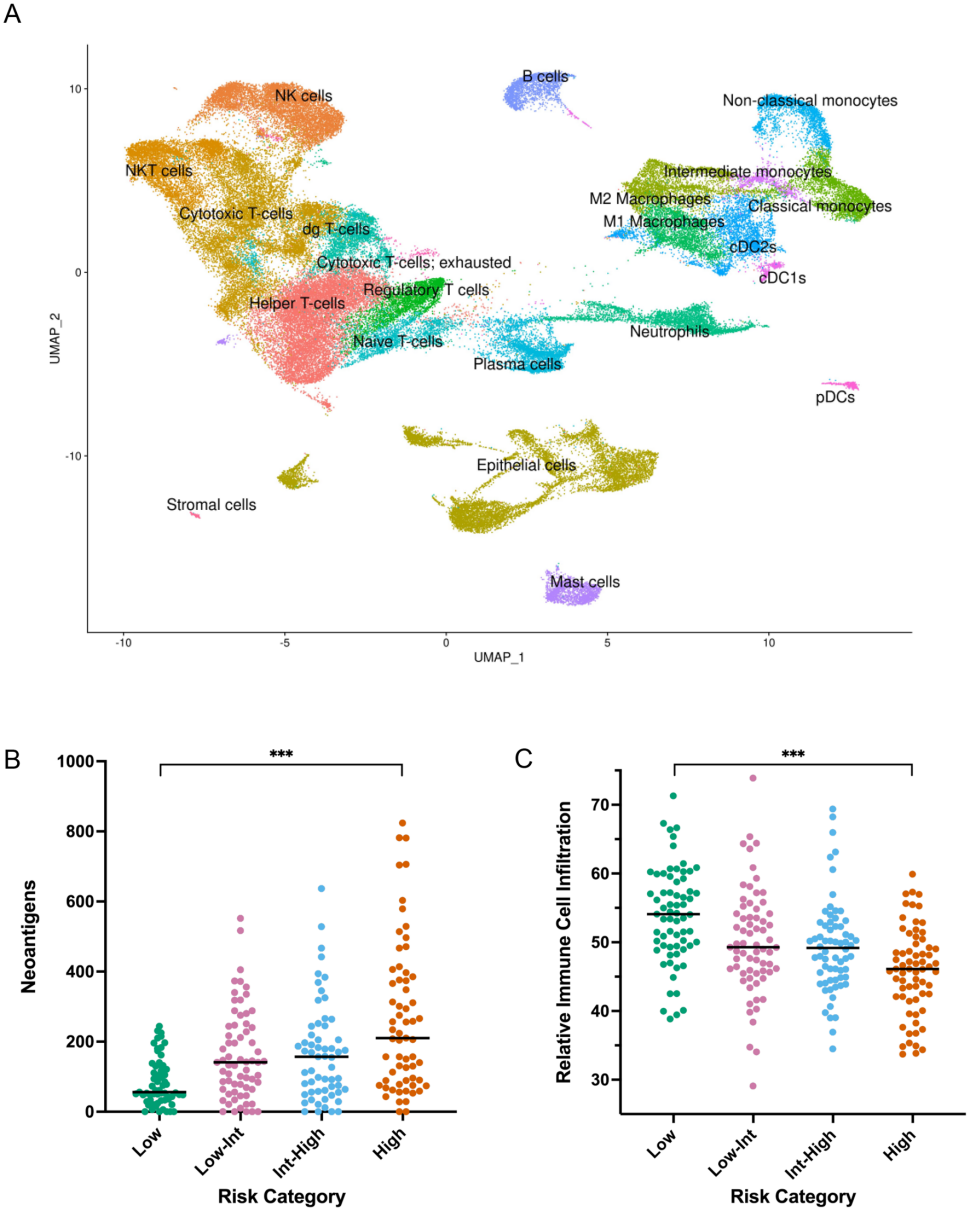
Recurrent stage I lung adenocarcinomas display immune desert phenotypes. scRNA-seq analysis reveals twenty distinct immune cell populations in stage I lung adenocarcinomas (**A**). Despite increasing antigenicity (**B**), tumors with increasing risk of recurrence demonstrate decreasing immune cell infiltration (**C**).

Other groups have used murine- and blood-based immune population gene profiles to characterize lung adenocarcinoma immune microenvironments with digital cytometry^21,22,25^. In order to develop human tumor infiltrating leukocyte profiles specific to stage I lung adenocarcinomas, we performed single-cell RNA-seq (scRNA-seq) analysis on freshly resected samples from six patients who underwent complete surgical resection of stage I tumors at UCSF. Twenty distinct immune cell populations were identified by scRNA-seq analysis including 10 adaptive immune populations (helper T-cells, cytotoxic T-cells, exhausted cytotoxic T-cells, δγ T-cells, naïve T-cells, regulatory T-cells, B cells, plasma cells, NKT cells, and NK cells) and 10 innate immune populations (M1 macrophages, M2 macrophages, classical monocytes, intermediate monocytes, non-classical monocytes, mast cells, conventional dendritic cells 1, conventional dendritic cells 2, plasmacytoid dendritic cells, and neutrophils) (Fig. 5A). These novel tumor infiltrating leukocyte profiles were used to calculate immune cell population densities from bulk RNA-seq data in the TCGA stage I lung adenocarcinoma population using digital cytometry.

**Figure 6:**
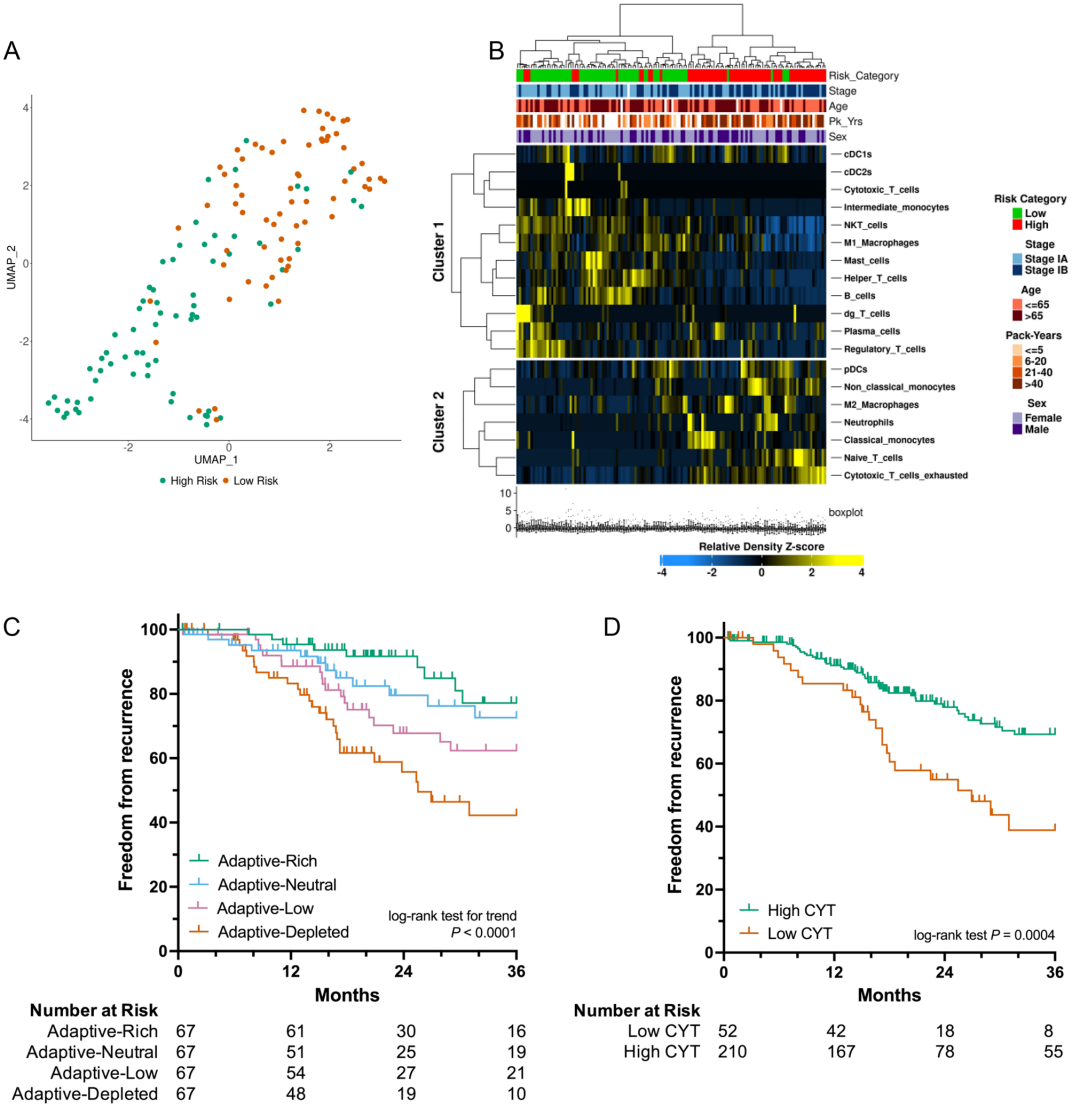
Recurrent stage I lung adenocarcinomas display adaptive immune cell depleted phenotypes. UMAP analysis reveals distinct tumor immune microenvironments between recurrence high- and low-risk tumors (**A**). Heatmap analysis demonstrates depletion of adaptive immune cell populations in recurrence high-risk tumors (**B**). Patients with adaptive-rich immune cell phenotypes have improved 24-month freedom from recurrence compared to tumors with adaptive-depleted phenotypes (**C**). High cytolytic activity score (CYT) is prognostic of decreased recurrence (**D**). ***P < 0.0001, **P < 0.001, *P < 0.05.

Despite a positive association between neoantigen burden and recurrence risk (73, 141, 173, and 234 predicted neoantigens in low-, low-intermediate, intermediate-high, and high-risk cohorts respectively [*P* < 0.0001 between low- and high-risk cohorts], Fig. 5B), a negative correlation between total immune cell infiltration and recurrence was observed (median relative immune cell densities of 54.1, 49.3, 49.0, and 46.1 in low-, low-intermediate, intermediate-high, and high-risk cohorts respectively [*P* < 0.0001 between low- and high-risk cohorts], Fig. 5C). In addition to differences in total immune cell infiltration, UMAP analysis demonstrated clear differences in the composition of the tumor immune microenvironment between recurrence high- and low-risk stage I tumors. When clustered by immune population densities only, separation of high- and low-risk tumors into distinct groups was observed (Fig. 6A). Heatmap clustering of immune populations also resulted in clear separation between recurrence high- and low-risk tumors across two distinct immune cell clusters (*P* < 0.0001, Fig. 6B). Stage (*P* = 0.0009) and smoking history (*P* = 0.0188) also showed separation by heatmap clustering. In contrast, age and sex were not significantly correlated with these immune cell clusters (Fig. 6B). Recurrence high-risk tumors were depleted in adaptive immune populations including cytotoxic T-cells, NKT cells, helper T-cells, B-cells, δγ T-cells, plasma cells, and T regulatory cells (Fig. 6B). In contrast, they were enriched for naïve T-cells and exhausted cytotoxic T-cells (Fig. 6B). Stromal FOXP3/CD3 ratio has been reported to positively correlate with increased recurrence^19^. In our cohort, however, regulatory T-cell/T-cell ratio was inversely correlated with risk of recurrence (Supplementary Fig. S5A and B).

Adaptive immune population density and activation was also prognostic of recurrence in our stage I lung adenocarcinoma cohort. To identify immune populations prognostic of recurrence, we utilized elastic net penalized regression analysis to determine the relative contribution of each immune population to patient prognosis. We observed that immune cells associated with humoral and cell-based adaptive immune responses including B-cells, mast cells, naïve T-cells, and exhausted cytotoxic T-cells were strongly associated with patient prognosis (Supplementary Table S6). Using the regression coefficients from this analysis, an immune profile classifier prognostic of recurrence was built. Patients with adaptive-rich, adaptive-neutral, adaptive-low, and adaptive-depleted phenotypes had 24-month FFR of 91.7%, 79.6%, 67.8%, and 55.7% respectively (*P* < 0.0001, Fig. 6C). Cytolytic activity associated with robust adaptive immune responses was also prognostic of recurrence in our cohort. Granzyme A and perforin expression has been validated as a reliable indicator of functional cytotoxic T-cell and NK cell activation^26^. High cytolytic activity was associated with a 24-month FFR of 77.9% versus 54.9% for patients with low cytolytic activity (*P* = 0.0004, Fig. 6D).

## Discussion

Although recurrence in early-stage adenocarcinoma is common, the genetic and immunologic features of recurrence in lung cancer are not well described. One approach to describing these features is to treat recurrence as a binary event by grouping patients into cohorts of “recurred” vs “did not recur”. Recurrence, however, is a time-to-event datapoint. Early recurrence is not the same biological event as late recurrence. In addition, patients who have not recurred may only be labeled as such as they have not been followed long enough to experience recurrence. Lastly, recurrence is notoriously difficult to track accurately in the real world as many patients are lost to follow-up after surgery and other treatments for early-stage disease^27^. Using recurrence as a binary endpoint to group patients, therefore, is suboptimal to understand the biological features of recurrence. To address these concerns and incorporate time-to-recurrence data as an endpoint in this study, a recurrence score was developed which relates the patient’s probability of developing recurrence to the biological features of the tumor. This strategy not only takes time-to-event data into account, but also lessens the impact of inaccurate clinical follow-up by simultaneously clustering the genomic profiles of these patients with their clinical endpoints. In the clinical setting, clustering patients by biological features related to time-to-event outcomes is a superior strategy that has been proven to add prognostic and predictive value in multiple studies^2,6–8,10,12,13,28^. The novel recurrence score described here is predictive of recurrence in the TCGA lung adenocarcinoma cohort and allows for a detailed investigation of the biological features of early-stage recurrence.

Tumor mutation burden (TMB) has been shown to be predictive of immunotherapy response in prior studies but has not been reported to be associated with recurrence^29^. In this study, early-stage lung adenocarcinomas with higher TMB had significantly higher rates of recurrence. Copy number alterations also occurred more frequently in recurrent tumors, signifying genomic instability as a hallmark of recurrence. *TP53* was the most frequently mutated gene in the stage I cohort and was more frequently mutated in recurrent tumors in our study. *MUC16* (*CA-125*) was also observed to be frequently mutated in recurrent tumors. MUC16 is overexpressed in several cancers including ovarian, pancreatic, breast, and lung, and has been shown to be associated with cancer progression and poor prognosis^30^. *SMARCA4*, also frequently mutated in recurrent tumors in our study, has been previously shown to be associated with recurrence in lung adenocarcinoma^16,17^. Mutations in *SMARCA4* can co-occur with *KRAS*, *STK11*, and *KEAP1* mutations^31^ and are prognostic of poor overall survival, especially in patients with *KRAS*-mutant tumors^31^. *MUC16* and *SMARCA4* are attractive therapeutic targets in patients at high-risk for recurrence after surgery for early-stage lung cancer and may warrant further investigation.

Other frequently mutated genes in our study included very large genes such as *TTN* (304,814 bases), *CSMD3* (1,214,572 bases), *RYR2* (791,805 bases), and *LRP1B* (1,901,041 bases). The presence of mutations in these large genes suggests that many mutations in recurrent tumors are random rather than targeted or selected events. With respect to canonical lung cancer driver mutations, previous studies have failed to demonstrate strong correlations between canonical lung cancer driver mutations and recurrence^16,17^. This was also observed in our study where no canonical driver mutations were found to occur more frequently in recurrent tumors, suggesting that the biological processes of tumor initiation and propagation are distinct. These distinct processes suggest that our current therapies targeting these canonical driver mutations, while beneficial in late-stage disease, may not be able to be widely applied to prevent recurrence in early-stage lung cancer.

Transcriptomic analysis revealed discrete genomic and immunologic features associated with early-stage lung adenocarcinoma recurrence. As expected, a number of canonical cancer-related pathways involving cell cycle control, metaphase signaling, DNA damage response, mitosis, checkpoint control, and mismatch repair are strongly activated in recurrent tumors. These cancer pathways have previously been described as hallmarks of cancer progression and metastasis^32–35^. Hypomethylation has also been shown in previous work to be associated with genome instability and cancer progression^36^. We also observed decreasing methylation in recurrent tumor phenotypes, which may partly explain the mutational instability and active cell cycle progression observed in the pathways above. Overall, the activation of these pathways and decreasing levels of methylation support the notion of an active, unstable genome in recurrent stage I lung adenocarcinomas. The overexpressed genes in recurrent lung cancers described here represent attractive small molecule inhibitor targets.

In contrast to the activation of numerous cancer-related pathways, a number of key immune response pathways are strongly downregulated in recurrent tumors. This suggests that the presence of a permissive tumor microenvironment is also an important cause of early-stage lung adenocarcinoma recurrence. In support of this, recurrent tumors display less overall immune cell infiltration despite having higher mutation and neoantigen burden than non-recurrent tumors, consistent with a “cold” tumor landscape. In addition, the top genes present in the downregulated transcriptome cluster of recurrent tumors play vital roles in antigen presentation and Th1 and Th2 activation. Antigen presentation and Th1 and Th2 activation pathways are intimately associated with each other as antigen presentation by MHC molecules stimulates Th1 and Th2 differentiation and activation^37^. Notably, epigenetic analysis of recurrent tumors demonstrates heavy methylation of antigen presentation pathway genes including a specific region of chromosome 6 containing numerous class II MHC genes, suggesting that hypermethylation is one mechanism of antigen presentation suppression. Epigenetic-targeted therapies have been effective in hematologic malignancies and solid tumor clinical trials^38^. Further study of epigenetic-targeted therapies that activate these antigen presentation genes in early-stage lung cancers at high-risk for recurrence may be warranted.

Other groups have used two main strategies to profile the tumor immune microenvironment in early-stage lung adenocarcinoma: 1. immunohistochemistry that allows for direct measurements of a limited number of specific immune cell populations and 2. computational techniques that allow for more comprehensive but indirect measurements of multiple immune cell populations. In the largest study of immune populations in stage I lung adenocarcinoma patients to date, Suzuki et al. investigated eight types of tumor-infiltrating leukocytes and five cytokines using immunohistochemistry^19^. This group observed that stromal FoxP3/CD3 ratio, tumor IL-12Rβ2, and tumor IL-7R were associated with recurrence^19^. Yan et al., in contrast, did not find a significant association between FoxP3-positive T regulatory cells and disease-free survival in their characterization of stage I-IV non-small cell lung cancer patients using immunohistochemistry^20^. Although our data also do not support this positive association between T regulatory cells and recurrence, this lack of concordance may be due to the fact that neither our group nor Yan et al. distinguished between tumor nest or stromal T regulatory cell populations. Varn et al. used computational techniques to characterize CD4+ T-cell, CD8+ T-cell, naïve B cell, memory B cell, NK cell, and myeloid cell populations in lung adenocarcinoma^22^. Murine immune cell gene expression profiles were used to develop consensus immune signatures for these six different immune populations. Consistent with our observations, they observed that low naïve B cell, low CD8+ T-cell populations, and high monocyte populations were associated with recurrence in stage I lung adenocarcinomas^22^. Other groups have used blood-based immune profiles to comprehensively characterize infiltrating immune populations in lung adenocarcinoma^21,25^, however, the association between these immune populations and recurrence was not reported. To our knowledge, this study is the first to develop novel tumor infiltrating leukocyte profiles in stage I lung adenocarcinoma using scRNA-seq and apply these profiles to comprehensively characterize immune cell populations in a large cohort of patients with recurrent stage I adenocarcinoma.

Using this approach, we observed that the lack of sufficient antigen presentation and T-helper cell response in recurrent stage I tumors by transcriptomic analysis is supported by our analysis of specific infiltrating immune populations in recurrent vs. non-recurrent tumors. Although dendritic cell and macrophage populations are similar between recurrent and non-recurrent tumors, non-recurrent tumors are heavily infiltrated by B-cells while recurrent tumors are relatively lacking in these antigen-presenting cells. In addition, recurrent tumors exhibit relatively sparse populations of helper T-cells while displaying a high proportion of exhausted cytotoxic T-cells. These data suggest that recurrent tumors may evade immunosurveillance by active downregulation of antigen presentation to T-helper cells, resulting in an insufficient adaptive immune response. In support of this, we observed that adaptive-depleted tumors had significantly worse prognosis that adaptive-rich tumors, recurring five-times more frequently within two years of diagnosis. In addition, cytolytic activity associated with functional T-cell and NK cell activation in the setting of a robust adaptive immune response was also predictive of recurrence. Stage I patients were twice as likely to recur if low cytolytic activity was measured in their tumors compared to patients with high cytolytic activity tumors. These data suggest that checkpoint inhibitors may not be maximally effective in preventing early-stage lung cancer recurrence as they are most effective in the presence of “hot” or inflamed tumor microenvironments^18^. Novel immunotherapies designed to attract and activate adaptive immune populations in early-stage tumors at high-risk of recurrence may be necessary and warrant further investigation.

There are several limitations to this study. First, recurrence is a time-to-event datapoint and the use of the TCGA lung adenocarcinoma cohort does not allow for independent verification of patient follow-up data such as recurrence and time-to-recurrence. Although the creation of a recurrence score helps mitigate inaccurate outcomes reporting and inadequate patient follow-up in this setting, the accuracy of the recurrence score used in this study could be improved with independent validation of clinical outcomes data. Second, although the TCGA cohort is the largest-ever assembled lung adenocarcinoma patient cohort with full genetic, genomic and epigenetic analysis, only 268 stage I patients were fully analyzed in this study. It is likely that stronger biological conclusions could be reached with a larger stage I lung adenocarcinoma patient cohort. Lastly, the use of computational techniques to quantify immune populations present in this large stage I lung adenocarcinoma cohort was necessary as direct measurements of immune cell densities were not available. Single-cell RNA-seq and spatial transcriptomics data will one day be available in large cohorts such as the one analyzed here and allow for direct measurements of immune cell infiltration, integrated immune cell transcriptome analysis, and differentiation between tumor and stromal immune cell populations.

In conclusion, recurrence in early-stage lung adenocarcinoma is common despite complete surgical resection as the mainstay of therapy. Recurrent stage I lung adenocarcinomas display features of genomic and genetic instability including increased tumor mutation burden, neoantigen load, activation of numerous mitotic and cell cycle genes, and decreased genome-wide methylation burden. In parallel, impaired antigen presentation and Th1/Th2 responses appear to be central features of permissive tumor immune microenvironments in recurrent tumors and may be responsible for worse prognosis associated with adaptive-depleted tumors.

## Methods

### Patient cohorts

Tumor and normal samples from 500 patients with complete follow-up in the TCGA lung adenocarcinoma cohort^39^ were included in this study (Supplementary Table S7). Indexed clinical data were downloaded from the Genomic Data Commons (GDC) portal (TCGAbiolinks v2.18.0^40^). Clinical outcomes including survival and recurrence endpoints were obtained from the TCGA Clinical Data Resource^41^.

To perform tumor immunoprofiling, additional tumor and adjacent normal tissue samples from six patients were characterized. Males and females between the ages of 18–75 who underwent complete surgical resection of pathologic stage I lung adenocarcinoma via lobectomy and mediastinal lymph node dissection at UCSF between 2019 and 2020 were included in this study. Patients who had higher stage disease, positive margins, no nodal dissection, or who underwent neoadjuvant therapy prior to resection were excluded from this study. All patients gave informed consent for sample collection. Samples were collected in accordance with UCSF ethical guidelines and regulations for the conduct of responsible research. The study was approved by the UCSF Institutional Review Board (IRB# 11-06107).

### Recurrence score

Tumor and normal RNA-seq TOIL recompute counts^42^ from the TCGA Pan-Cancer (PANCAN) cohort were downloaded from the UCSC Xena browser^43^. Gene counts-per-million (CPM) for each tumor and normal sample were generated using edgeR (v3.32.1). To create a risk score associated with recurrence, a combination of *L1*- and *L2*-penalized Cox proportional hazards modeling (R package glmnet v4.1.1) was used with progression-free interval (PFI.1 in the TCGA Clinical Data Resource^41^) as an endpoint. Using tenfold cross-validation, the amount of *L1*-penalization was increased until a parsimonious set of genes that maximized the concordance index was obtained (Supplementary Table S2). *L2*-penalization was simultaneously applied in order to reduce model overfitting as much as possible. A continuous recurrence score was generated for each tumor sample by summing the product of the CPM value and the cox proportional hazards model coefficient for each model gene. Resultant predicted risk scores were divided at the 25th, 50th, and 75th percentiles to generate low-, low-intermediate-, intermediate-high, and high-risk groups.

### Power calculation

Power calculations using one-way analysis of variance and a two-tailed test were performed. Dividing the entire patient cohort (n = 500) into quartiles yielded an 80% probability of detecting a difference of 14.8% and 90% probability of detecting a difference of 16.9% between recurrence risk groups. Dividing the stage I patient cohort (n = 268) into quartiles yielded an 80% probability of detecting a difference of 20.3% and 90% probability of detecting a difference of 23.2% between recurrence risk groups.

### Genomic alteration analysis

Mutation Annotation Format (MAF) files were downloaded from the Genomic Data Portal (R package TCGAbiolinks^40^ (v2.18.0). The tumor mutation burden for each sample was assessed using the R package maftools (v2.7.10). Differentially mutated genes were detected by performing Fisher exact tests on all genes between recurrence low- and recurrence high-risk cohorts. To analyze copy number alterations in each patient tumor sample, sample level copy number alteration data were downloaded from the Broad GDAC Firehose pipeline (R package RTCGAToolbox v2.20.0). Amplification data from this analysis and gene fusions identified using RNA-seq analysis from Hu et al.^44^ for each patient in our study cohort were added to the MAF files to generate combined oncoplots. To analyze genome-wide differences in copy number alterations across recurrence low- vs. high-risk cohorts, Affymetrix Genome-Wide Human SNP Array 6.0 data were downloaded from the GDC portal. Significant recurrent copy number alterations were identified using Genomic Analysis of Important Alterations (R package GAIA v2.34.0). Taking within-sample homogeneity into account, GAIA performs a conservative permutation test to extract the regions most likely involved in functional phenotype changes. Recurrent amplifications and deletions were identified for each recurrence risk cohort and annotated; genomic regions identified as significantly altered in copy number (adjusted *P-*value < 10^−4^) were represented on chromosome overview plots.

### Transcriptome and methylation analysis

Differential gene expression between recurrence low- versus high-risk cohorts was performed with TOIL recompute counts (described above) using edgeR (R package v3.32.1), limma (R package 3.46.0), and voom^45^. A heatmap of differential gene expression was produced using the ComplexHeatmap package in R (v2.6.2). Partitioning Around Medoids (PAM) (R package cluster v2.1.0) was used to partition genes into distinct genomic clusters. Clinical covariates including recurrence risk category, age, stage, gender, and smoking history were used to annotate the heatmap. Fisher exact tests were used to evaluate for statistical significance between heatmap clusters and clinical covariates. Conventional pathway analysis on the top differentially expressed genes in each cluster was performed using IPA (Qiagen, Germantown, PA). Topological pathway analysis was performed on the top differentially expressed genes using the R package SPIA (v2.42.0).

For methylation analysis, Illumina Human Methylation 450 array data aligned to homo sapiens (human) genome assembly GRCh37 (hg19) was downloaded from the Genomic Data Portal (R package TCGAbiolinks^40^ (v2.18.0). Methylation data was annotated and filtered to remove unmapped probes, probes mapped to multiple places in the genome^46^, and duplicate probes as previously described^47^. Differential gene methylation between recurrence low- vs. high-risk cohorts was performed with M-values using edgeR (R package v3.32.1) and limma (R package 3.46.0). A heatmap of differential gene methylation was produced using the ComplexHeatmap package in R (v2.6.2). Partitioning Around Medoids (PAM) (R package cluster v2.1.0) was used to partition methylated genes into distinct genomic clusters. Clinical covariates including recurrence risk category, age, stage, gender, and smoking history were used to annotate the heatmap. Differentially methylated regions were defined and mapped to the chromosome as previously described^47^. Conventional pathway analysis on the top differentially methylated genes was performed using GO, KEGG, and GSEA Molecular Signatures Database Hallmark Pathways as previously described^47^. Upstream regulation of gene enhancers by transcription factors was analyzed by integration of annotated methylation and gene expression data using the ELMER R package (v2.14.0).

### Tumor immunoprofiling

Freshly resected tumor or adjacent normal lung tissue was thoroughly chopped with surgical scissors and transferred to GentleMACS C Tubes (Miltenyi Biotec) containing 20 µL/mL Liberase TL (5 mg/ml, Roche) and 50 U/ml DNAse I (Roche) in Leibovitz’s L-15 per 0.3 g tissue. GentleMACS C Tubes were then installed onto the GentleMACS Octo Dissociator (Miltenyi Biotec) and incubated for 45 min according to the manufacturer’s instructions. Samples were quenched with 15 mL of sort buffer (PBS/2% FCS/2 mM EDTA), filtered through 100 um filters and spun down. Red blood cell lysis was performed with 175 mM ammonium chloride if needed.

Cells were subsequently incubated with with Zombie Aqua Fixable Viability Dye (Thermo), washed with sort buffer, and incubated with Human FcX (BioLegend) to prevent non-specific antibody binding. Following FcX incubation, cells were washed with sort buffer and incubated with cell surface antibodies mix diluted in the BV stain buffer (BD Biosciences) following manufacturer instructions for 30 min on ice in the dark. Live cells were sorted from these single cell suspensions on a BD FACSAria Fusion. After sorting, cells were pelleted and resuspended at 1 × 10^3^ cells/ml in 0.04% BSA/PBS and loaded onto the Chromium Controller (10x Genomics). Samples were processed for single-cell encapsulation and cDNA library generation using the Chromium Single Cell 3’ v3 Reagent Kits (10x Genomics). In order to duplex tumor and normal cells, cell hashing was performed using TotalSeq-A antibodies (BioLegend) according to manufacturer instructions. Libraries were subsequently sequenced on an Illumina HiSeq 4000 (Illumina).

Sequences from scRNA-seq were processed with Cellranger (10x Genomics) and read into the Seurat R package. The filtered count matrices were normalized and variance stabilized using negative binomial regression via the scTransform method^48^. Mitochondrial content, ribosomal content, and cell cycle state were regressed from the normalized data. Datasets from twelve samples (six tumor and six normal) were integrated using the anchoring method described by Stuart et al.^49^. The integrated matrix underwent dimensionality reduction using Principal Component Analyses (PCA); the first 40 principal coordinates were subjected to non-linear dimensionality reduction using Uniform Manifold Approximation and Projection (UMAP). Clusters of cells sharing similar transcriptomic profiles were identified using the Louvain algorithm with a clustering resolution of 0.8. Differentially expressed genes for each cluster were identified using the Wilcox test in Seurat and top cluster genes (log fold change > 0.4) were used to identify cell clusters.

Measurements of immune cell population densities was performed using the digital cytometry method described by Newman et al.^50^. This method utilizes single-cell reference profiles to calculate cell type frequencies from bulk transcriptome data. 10,000 labeled cells from the scRNA-seq experiments above were used to create a single cell reference matrix. Cell fractions were imputed from RNA-seq data from the entire TCGA lung adenocarcinoma cohort in absolute mode with quantile normalization disabled. To cluster tumors by their immune profiles, a combination of *L1*- and *L2*-penalized Cox proportional hazards modeling (R package glmnet v4.1.1) was used as described above with immune cell population densities as predictors and recurrence as an endpoint. Using tenfold cross-validation, unbiased increase in the amount of *L1*-penalization resulted in a parsimonious set of immune cell population densities predictive of recurrence. Neutrophils were excluded from this analysis due to their overwhelming abundance relative to other immune populations. An immune profile score was generated for each tumor sample by summing the product of density values for each immune cell population in the model by cox proportional hazards model coefficients. This immune profile score was enriched for immune populations associated with humoral and cellular adaptive immune responses (Supplementary Table S7). Patients were clustered into adaptive-depleted, adaptive-low, adaptive-neutral, and adaptive-rich cohorts by separating immune profile scores at the 25th, 50th, and 75th percentiles. Transcript levels of granzyme A (*GZMA*) and perforin (*PRF1*) have previously been reported to be reliable measures of local immune cytolytic activity in TCGA tumors^26^. Following previously validated methods^26^, cytolytic activity (CYT) in this study was calculated as the geometric mean of the CPM values of *GZMA* and *PRF1* for each sample in our cohort. Patients were grouped into low- and high-cytolytic activity groups using a survival cutpoint determined by the R package survminer (v0.4.9).

### Statistical analysis

The primary endpoint in this study was recurrence (PFI.1 in the TCGA Clinical Data Resource^41^). The primary predictors evaluated were recurrence risk score, recurrence risk category, age, sex, smoking history, and stage. These predictors were correlated with recurrence using univariate and multivariate Cox proportional hazards modeling. Wald tests were performed to evaluate for statistical significance in Cox proportional hazards modeling. Stratified Kaplan–Meier analysis using a right-censored dataset and the log-rank test for trend were used to evaluate the correlation between primary predictors and the primary endpoint. Time-dependent Area Under the Receiver Operating Characteristic curve (AUROC) was calculated using the survcomp (v1.4.0) package in R. Differences in AUROCs were tested by multivariate Cox proportional hazards modeling, integrated AUROCs were compared by Wilcoxon rank sum test. For all statistical tests, a pre-specified two-sided α of 0.05 was considered significant. Adjusted *P*-values are reported for all analyses involving multiple comparisons. Analyses were conducted using the programming languages R (v4.0.3 for Macintosh), STATA/SE (v15.1, StataCorp, College Station, TX) and Prism (v9.1.0, GraphPad Software, San Diego, CA).

## Supplementary Information

Supplementary Information.

## Data Availability

The Cancer Genome Atlas (TCGA) datasets analyzed during the current study are publicly available for download from the National Cancer Institute Genomic Data Commons Data Portal (https://portal.gdc.cancer.gov). The single-cell RNA-seq datasets generated and analyzed during the current study are available from the corresponding author on reasonable request.

## References

[1] Herbst RS, Morgensztern D, Boshoff C (2018). The biology and management of non-small cell lung cancer. Nature.

[2] Woodard, G. A. et al. Adjuvant chemotherapy guided by molecular profiling and improved outcomes in early stage, non-small-cell lung cancer. *Clin. Lung Cancer* (2017). 10.1016/j.cllc.2017.05.015 28645632

[3] Burdett, S. et al. Adjuvant chemotherapy for resected early-stage non-small cell lung cancer. *Cochrane Database Syst Rev* CD011430 (2015). 10.1002/14651858.CD011430 PMC10542092 25730344

[4] Chansky K (2017). The IASLC lung cancer staging project: external validation of the revision of the TNM stage groupings in the eighth edition of the TNM classification of lung cancer. J. Thorac. Oncol..

[5] Tang H (2017). Comprehensive evaluation of published gene expression prognostic signatures for biomarker-based lung cancer clinical studies. Ann. Oncol..

[6] Kratz JR, Jablons DM (2009). Genomic prognostic models in early-stage lung cancer. Clin. Lung Cancer.

[7] Kratz JR (2012). A practical molecular assay to predict survival in resected non-squamous, non-small-cell lung cancer: development and international validation studies. Lancet.

[8] Kratz JR, Van den Eeden SK, He J, Jablons DM, Mann MJ (2012). A prognostic assay to identify patients at high risk of mortality despite small, node-negative lung tumors. JAMA J. Am. Med. Assoc..

[9] Kratz JR (2013). Analytical validation of a practical molecular assay prognostic of survival in nonsquamous non-small cell lung cancer. Diagn. Mol. Pathol..

[10] Kratz JR, Mann MJ, Jablons DM (2013). International trial of adjuvant therapy in high risk stage I non-squamous cell carcinoma identified by a 14-gene prognostic signature. Transl. Lung Cancer Res..

[11] Woodard GA (2018). Adjuvant chemotherapy guided by molecular profiling and improved outcomes in early stage non-small-cell lung cancer. Clin. Lung Cancer.

[12] Kratz JR (2019). Incorporation of a molecular prognostic classifier improves conventional non-small cell lung cancer staging. J. Thorac. Oncol..

[13] Haro GJ (2019). Comparison of conventional TNM and novel TNMB staging systems for non-small cell lung cancer. JAMA Netw. Open.

[14] Campbell JD (2016). Distinct patterns of somatic genome alterations in lung adenocarcinomas and squamous cell carcinomas. Nat. Genet..

[15] Cancer GARN (2014). Comprehensive molecular profiling of lung adenocarcinoma. Nature.

[16] Jones GD (2021). A genomic-pathologic annotated risk model to predict recurrence in early-stage lung adenocarcinoma. JAMA Surg..

[17] Cho WCS (2018). Targeted next-generation sequencing reveals recurrence-associated genomic alterations in early-stage non-small cell lung cancer. Oncotarget.

[18] Chen DS, Mellman I (2017). Elements of cancer immunity and the cancer-immune set point. Nature.

[19] Suzuki K (2013). Clinical impact of immune microenvironment in stage I lung adenocarcinoma: tumor interleukin-12 receptor β2 (IL-12Rβ2), IL-7R, and stromal FoxP3/CD3 ratio are independent predictors of recurrence. J. Clin. Oncol..

[20] Yan X, Jiao SC, Zhang GQ, Guan Y, Wang JL (2017). Tumor-associated immune factors are associated with recurrence and metastasis in non-small cell lung cancer. Cancer Gene Ther..

[21] Liu X (2017). The prognostic landscape of tumor-infiltrating immune cell and immunomodulators in lung cancer. Biomed. Pharmacother..

[22] Varn FS, Tafe LJ, Amos CI, Cheng C (2018). Computational immune profiling in lung adenocarcinoma reveals reproducible prognostic associations with implications for immunotherapy. Oncoimmunology.

[23] Skoulidis F, Heymach JV (2019). Co-occurring genomic alterations in non-small-cell lung cancer biology and therapy. Nat. Rev. Cancer.

[24] Ma J, Shojaie A, Michailidis G (2019). A comparative study of topology-based pathway enrichment analysis methods. BMC Bioinform..

[25] Yu X, Wang X (2018). Tumor immunity landscape in non-small cell lung cancer. PeerJ.

[26] Rooney MS, Shukla SA, Wu CJ, Getz G, Hacohen N (2015). Molecular and genetic properties of tumors associated with local immune cytolytic activity. Cell.

[27] Black WC, Haggstrom DA, Welch HG (2002). All-cause mortality in randomized trials of cancer screening. J. Natl. Cancer Inst..

[28] Pardo JA (2021). The role of oncotype DX^®^ recurrence score in predicting axillary response after neoadjuvant chemotherapy in breast cancer. Ann Surg Oncol.

[29] Maleki Vareki S, Garrigós C, Duran I (2017). Biomarkers of response to PD-1/PD-L1 inhibition. Crit. Rev. Oncol. Hematol..

[30] Aithal A (2018). MUC16 as a novel target for cancer therapy. Expert Opin. Ther. Targets.

[31] Schoenfeld AJ (2020). The genomic landscape of SMARCA4 alterations and associations with outcomes in patients with lung cancer. Clin. Cancer Res..

[32] Clara JA, Monge C, Yang Y, Takebe N (2020). Targeting signalling pathways and the immune microenvironment of cancer stem cells: a clinical update. Nat. Rev. Clin. Oncol..

[33] Johnson, J. A. Targeting mutations that drive lung cancer. *Nature* (2020).

[34] Sanchez-Vega F (2018). Oncogenic signaling pathways in the cancer genome atlas. Cell.

[35] Sever R, Brugge JS (2015). Signal transduction in cancer. Cold Spring Harb. Perspect. Med..

[36] Sheaffer KL, Elliott EN, Kaestner KH (2016). DNA hypomethylation contributes to genomic instability and intestinal cancer initiation. Cancer Prev. Res. (Phila).

[37] Kaiko GE, Horvat JC, Beagley KW, Hansbro PM (2008). Immunological decision-making: how does the immune system decide to mount a helper T-cell response. Immunology.

[38] Cheng Y (2019). Targeting epigenetic regulators for cancer therapy: mechanisms and advances in clinical trials. Signal Transduct. Target Ther..

[39] Grossman RL (2016). Toward a shared vision for cancer genomic data. N. Engl. J. Med..

[40] Colaprico A (2016). TCGAbiolinks: an R/Bioconductor package for integrative analysis of TCGA data. Nucleic Acids Res.

[41] Liu J (2018). An integrated TCGA pan-cancer clinical data resource to drive high-quality survival outcome analytics. Cell.

[42] Vivian J (2017). Toil enables reproducible, open source, big biomedical data analyses. Nat. Biotechnol..

[43] Goldman MJ (2020). Visualizing and interpreting cancer genomics data via the Xena platform. Nat. Biotechnol..

[44] Hu X (2018). TumorFusions: an integrative resource for cancer-associated transcript fusions. Nucleic Acids Res..

[45] Law CW, Chen Y, Shi W, Smyth GK (2014). voom: Precision weights unlock linear model analysis tools for RNA-seq read counts. Genome Biol..

[46] Chen YA (2013). Discovery of cross-reactive probes and polymorphic CpGs in the Illumina Infinium HumanMethylation450 microarray. Epigenetics.

[47] Maksimovic J, Phipson B, Oshlack A (2016). A cross-package Bioconductor workflow for analysing methylation array data. F1000Res.

[48] Hafemeister C, Satija R (2019). Normalization and variance stabilization of single-cell RNA-seq data using regularized negative binomial regression. Genome Biol..

[49] Stuart T (2019). Comprehensive integration of single-cell data. Cell.

[50] Newman AM (2019). Determining cell type abundance and expression from bulk tissues with digital cytometry. Nat. Biotechnol..

