# Supplementary material for: Genetic and immunologic features of recurrent stage I lung adenocarcinoma
Source: Sci Rep. 2021 Dec 8;11:23690. doi: 10.1038/s41598-021-02946-0 (PMC8654957; doi:10.1038/s41598-021-02946-0)
Supplement: Supplementary file 1 — Supplementary Information. [file 41598_2021_2946_MOESM1_ESM.pdf]

Supplemental Figure 1

A

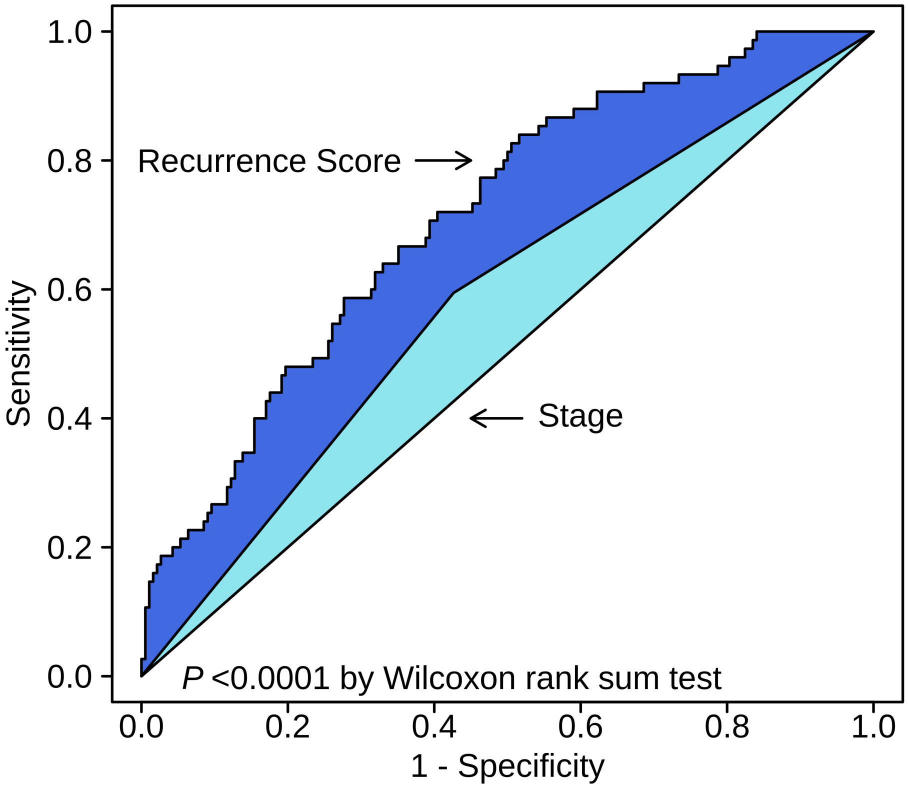

Supplemental Figure 1. Recurrence Score improves recurrence predictions vs. stage alone. The recurrence score increases the time-dependent AUROC to 0.714 from 0.584 vs. stage alone (P < 0.001).

Supplemental Figure 2

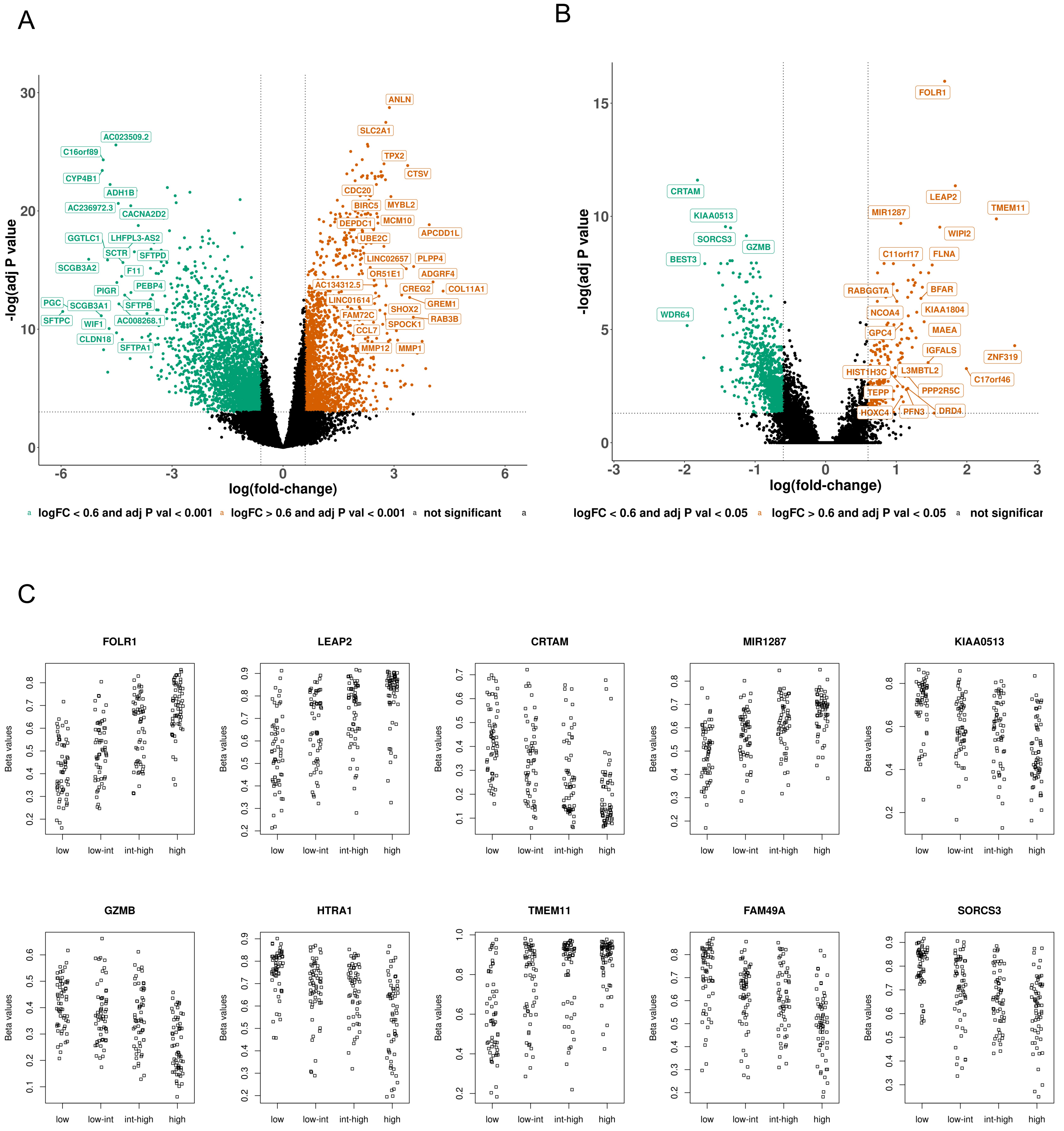

Supplemental Figure 2. Genomic and epigenetic alterations in recurrent high- vs. low-risk stage I lung adenocarcinomas. Volcano plots of differential gene expression (A) and differential methylation (B) in recurrent high- vs. low-risk tumors. The top 10 differentially methylated genes by recurrent risk category are shown in (C).

Supplemental Figure 3

A

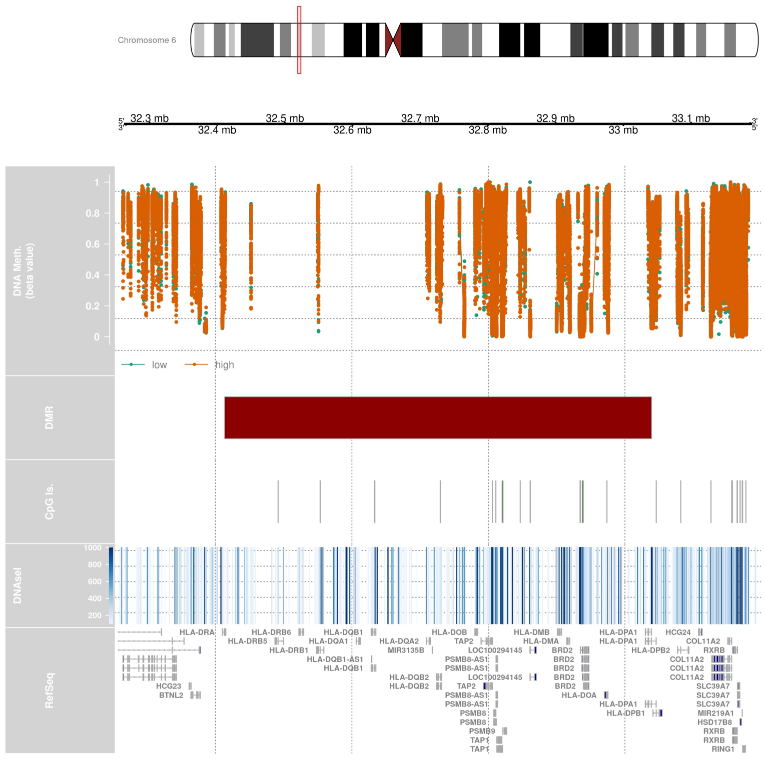

B

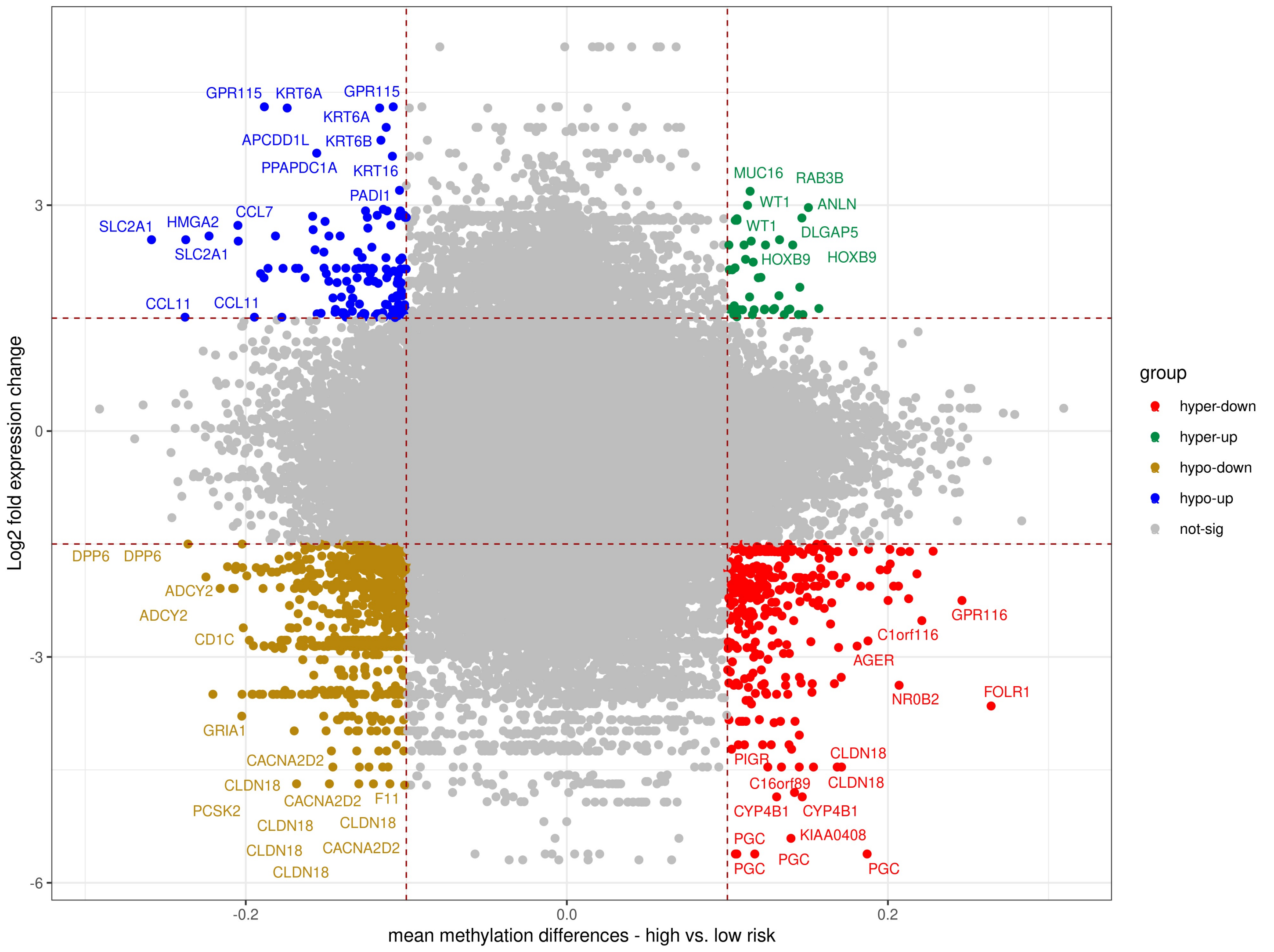

Supplemental Figure 3. Regional and integrated transcriptome methylation analysis reveals distinct features of recurrent high-risk stage I lung adenocarcinomas. Analysis of differentially methylated regions (DMRs) demonstrates heavy methylation of the chromosome 6 region containing class II MHC genes (A). Integrated transcriptome and methylation analysis demonstrates a total of 295 genes that are both hypomethylated and upregulated (blue quadrant) and 771 genes that are both hypermethylated and downregulated (red quadrant) in high- vs. low-risk patients (B).

Supplemental Figure 4

A

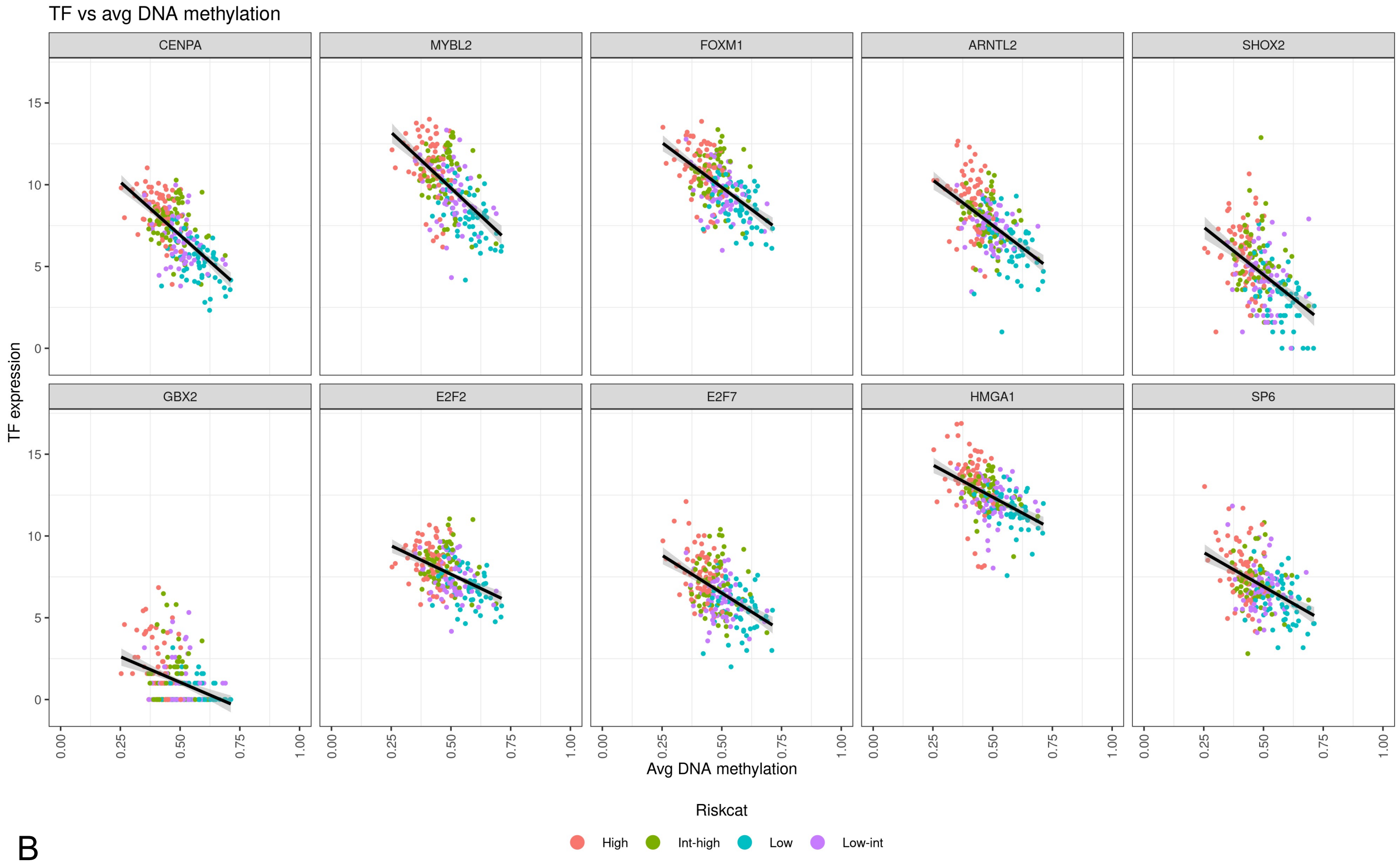

B

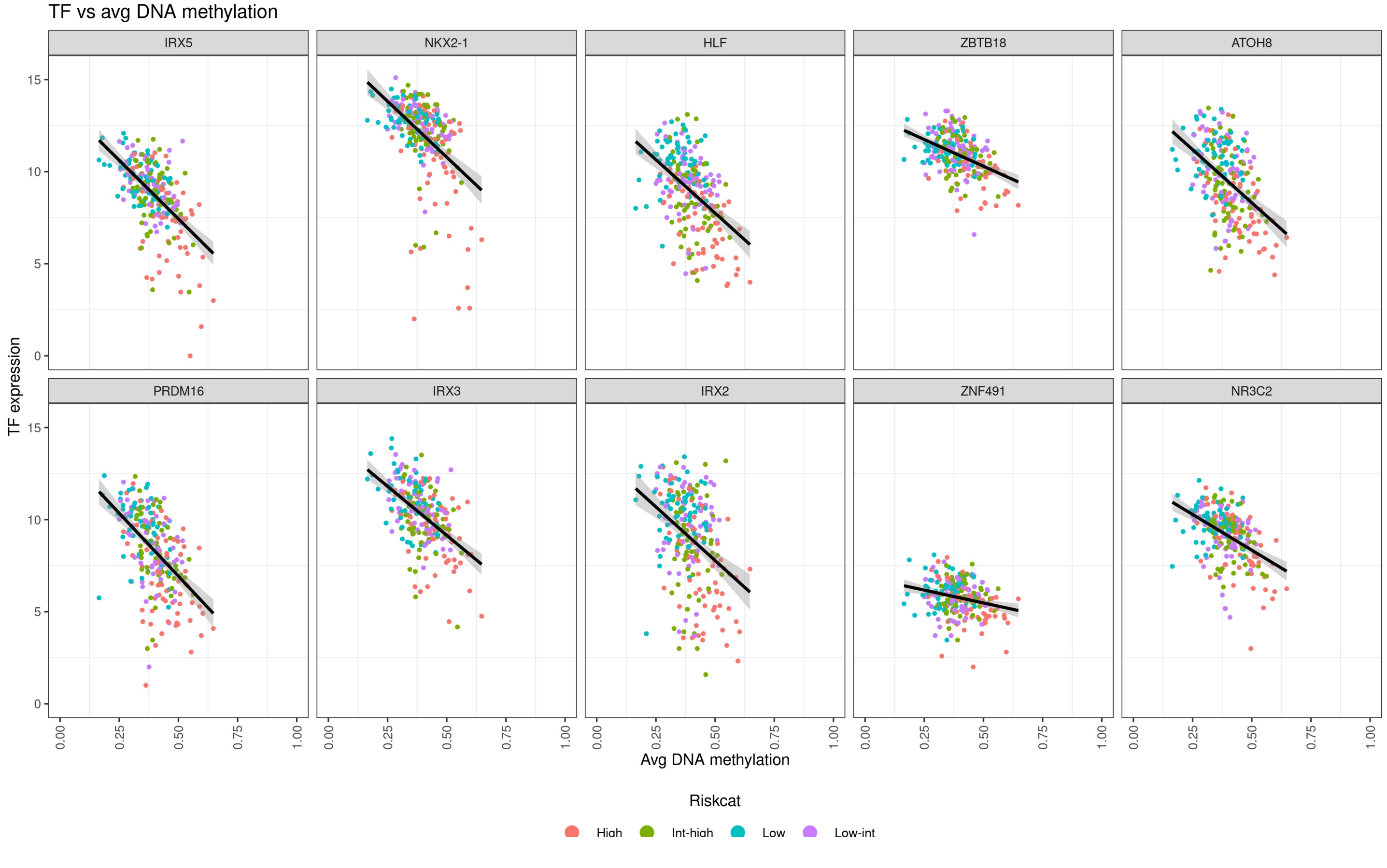

Supplemental Figure 4. Integrated analysis using enhancer linking identifies multiple differentially methylated transcription factor promoter binding sites. The top 10 upregulated transcription factors with hypomethylated promoter binding sites (A) and downregulated transcription factors with hypermethylated promoter binding sites (B) are shown; recurrence risk categories are labeled by color.

Supplemental Figure 5

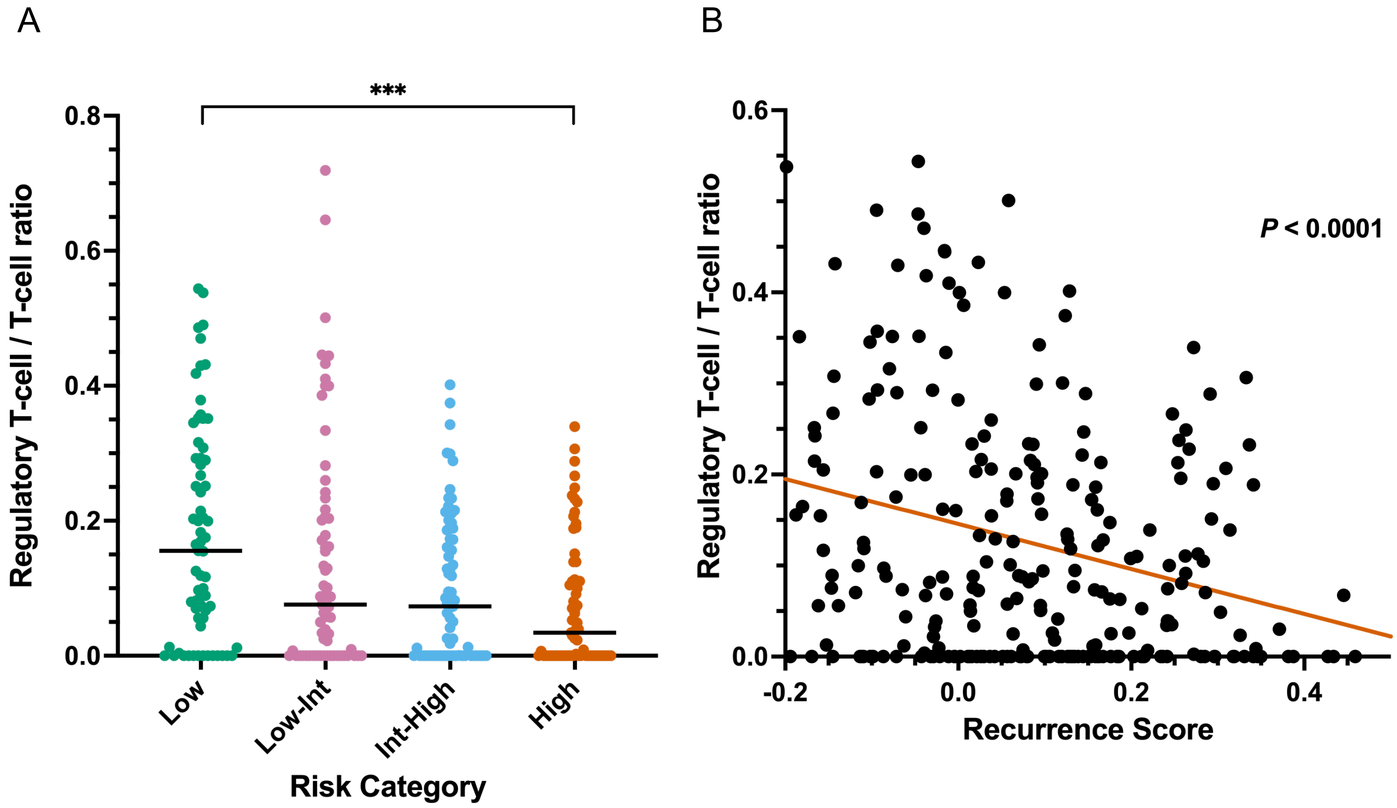

Supplemental Figure 5. Regulatory T-cell / T-cell ratio and cytolytic activity are prognostic of recurrence in stage I lung adenocarcinoma. Regulatory T-cell / T-cell ratio decreases in low- vs. higher recurrence risk categories (A) and is inversely correlated with recurrence risk score (B). ***P < 0.0001.

Supplemental Tables

Table S1. Genes and Coefficients in Recurrence Risk Score Model	
**Gene**	**Coefficient**
LDHA	0.011638925
PAX5	-0.00068286
AC046143.1	0.004911234
TANGO2	-0.00607408
ZNF341-AS1	0.009427997
CASP12	-0.005758507
ZNF763	-0.002287844
H3C13	0.004328153
FAM117A	-0.034132088
XRCC5	0.001544703
CIDEC	0.001305843
INPP5J	-0.000471615
ANLN	0.005611476
AL596223.1	0.002101452
AC079601.2	-0.001371535
BEST3	0.000201193
CCR6	-0.002009526
SKIL	0.010454531
KLRG2	-0.000898403
MPP6	0.003017565
AC004947.2	-0.004915431
AP000695.2	0.002646879
CREG2	0.002145222
SEC14L4	-0.001118089
LDLRAD3	0.020860561
KIR2DL3	-0.002720483
YWHABP2	0.022408439
AKAP12	0.002065057
LINC01117	0.009931264
BTBD7P1	-0.001467806
RHOV	0.001595022
OPN3	0.008829789
DKK1	0.004503076
CHRNA6	-0.012090302
FAM76A	-0.01635178
CYP4B1	-5.48E-05
CRHR2	-0.003313881
TLE1	0.022100011
PLEKHB1	-0.007319084
B4GALT1	0.008964576
TEX15	0.002233072
GNG7	-0.007211389
KIAA0408	-0.009124867
SYNPR-AS1	-0.011220243
LINC00667	-0.004310673
AC068228.2	0.009142372
AC127070.2	-0.003982105
AP000695.1	0.013513884
TMEM213	-0.005681923
SLC47A1	-0.001912309
VIM-AS1	-0.004906454
EPHX1	-0.003544239
AC145676.1	0.002301641
LINC01634	-0.008772685
NKILA	0.004137425
GOLM1	0.009448779
LINC00862	0.008308114
SLF1	0.004226585
ZC3H12D	-0.012195406
ZNF563	-0.000248725
ZCWPW1	-0.000592242
TRIM6	0.003242132
AC105942.1	-0.002783341
LINC01806	-0.00351529

Supplemental Table 1. Genes and Coefficients in Recurrence Risk Score Model. Elastic net penalized regression analysis was used to assign coefficients to each gene based on that gene’s contribution to patient prognosis. All genes with non-zero coefficients were included in the final model. A continuous recurrence score was generated for each tumor sample by summing the product of the CPM value and the cox proportional hazards model coefficient for each model gene.

Table S2. Pathway Analysis for Differential Gene Expression Heatmap Cluster 2

Ingenuity Canonical Pathways	-log(p-value)	Ratio	z-score	Molecules
Kinetochore Metaphase Signaling Pathway	2.95E+01	4.95E-01	3.202	ANAPC1,ANAPC7,AURKB,BIRC5,BUB1,BUB1B,BUB3,CCNB1,CDC20,CDC27,CDCA8,CDK1,CENPA,CENPE,CENPH,CENPK,CENPL,CENPN,CENPO,CENPP,CENPU,CENPW,DSN1,ESPL1,H2AX,H2AZ1,INCENP,KIF2C,KNL1,KNTC1,MAD2L1,MASTL,NDC80,NEK2,NUF2,PLK1,PPP1CC,PPP1R12A,PTTG1,RAD21,SKA1,SKA2,SKA3,SMC1B,SMC3,SPC24,SPC25,SPDL1,TTK,ZW10,ZWILCH,ZWINT
Cell Cycle Control of Chromosomal Replication	1.85E+01	5.36E-01	5.477	CDC45,CDC6,CDC7,CDK1,CDK12,CDK16,CDK2,CDK6,CDK8,CDT1,CHEK2,DBF4,DNA2,LIG1,MCM2,MCM3,MCM4,MCM5,MCM6,MCM7,MCM8,ORC1,ORC6,PCNA,POLA2,POLD1,POLE,PRIM1,PRIM2,TOP2A
Role of BRCA1 in DNA Damage Response	1.78E+01	4.38E-01	1.961	ACTB,BARD1,BLM,BRCA1,BRCA2,BRIP1,CHEK1,CHEK2,DPF1,E2F1,E2F2,E2F3,E2F7,E2F8,FANCA,FANCB,FANCC,FANCD2,FANCG,FANCL,FANCM,HLTF,MSH2,MSH6,NBN,PLK1,RAD51,RBBP8,RBL1,RFC2,RFC3,RFC4,RFC5,STAT1,TOPBP1
Mitotic Roles of Polo-Like Kinase	1.12E+01	3.79E-01	2.5	ANAPC1,ANAPC7,CCNB1,CCNB2,CDC20,CDC25A,CDC25C,CDC27,CDC7,CDK1,CHEK2,ESPL1,FBXO5,HSP90AA1,HSP90B1,KIF11,KIF23,PKMYT1,PLK1,PLK4,PPP2R1B,PRC1,PTTG1,RAD21,SMC3
Hereditary Breast Cancer Signaling	1.12E+01	2.68E-01	NaN	ACTB,BARD1,BLM,BRCA1,BRCA2,CCNB1,CDC25C,CDK1,CDK6,CHEK1,CHEK2,DPF1,E2F1,FANCA,FANCB,FANCC,FANCD2,FANCG,FANCL,FANCM,H2AX,HDAC2,HLTF,KRAS,MSH2,MSH6,NBN,NRAS,PALB2,RAD51,RALA,RAP2A,RAP2B,RFC2,RFC3,RFC4,RFC5,TUBG1
Role of CHK Proteins in Cell Cycle Checkpoint Control	9.28E+00	3.68E-01	-0.535	BRCA1,CDC25A,CDC25C,CDK1,CDK2,CHEK1,CHEK2,CLSPN,E2F1,E2F2,E2F3,E2F7,E2F8,NBN,PCNA,PLK1,PPP2R1B,RFC2,RFC3,RFC4,RFC5
Estrogen-mediated S-phase Entry	9.01E+00	5.38E-01	3.051	CCNA2,CCNE1,CCNE2,CDC25A,CDK1,CDK2,E2F1,E2F2,E2F3,E2F7,E2F8,RBL1,SKP2,TFDP1
RAN Signaling	8.36E+00	6.47E-01	3.317	CSE1L,IPO5,KPNA1,KPNA2,KPNA3,KPNA4,KPNB1,RANBP1,RANGAP1,RCC1,XPO1
ATM Signaling	7.92E+00	2.68E-01	1.043	BLM,BRCA1,CBX1,CBX3,CCNB1,CCNB2,CDC25A,CDC25C,CDK1,CDK2,CHEK1,CHEK2,FANCD2,H2AX,NBN,PPP1CC,PPP2R1B,RAD51,RBBP8,SMC1B,SMC2,SMC3,SUV39H1,TDP1,TOPBP1,TRIM28
Cell Cycle: G2/M DNA Damage Checkpoint Regulation	7.86E+00	3.60E-01	-1.069	AURKA,BORA,BRCA1,CCNB1,CCNB2,CDC25C,CDC34,CDK1,CHEK1,CHEK2,PKMYT1,PLK1,PRKDC,SKP2,TOP2A,YWHAG,YWHAH,YWHAZ
Mismatch Repair in Eukaryotes	7.43E+00	6.25E-01	NaN	EXO1,FEN1,MSH2,MSH6,PCNA,POLD1,RFC2,RFC3,RFC4,RFC5
Cyclins and Cell Cycle Regulation	7.27E+00	2.74E-01	3.153	CCNA2,CCNB1,CCNB2,CCNE1,CCNE2,CDC25A,CDC34,CDK1,CDK2,CDK6,CDKN2D,E2F1,E2F2,E2F3,E2F7,E2F8,HDAC2,PA2G4,PPP2R1B,RBL1,SKP2,SUV39H1,TFDP1
Remodeling of Epithelial Adherens Junctions	6.97E+00	2.94E-01	1	ACTB,ACTG1,ACTN1,ACTN4,ACTR2,ACTR3,ARF6,CBLL1,CLIP1,DNM1L,MAPRE1,RALA,TUBA1B,TUBA1C,TUBB,TUBB3,TUBB6,TUBG1,VCL,ZYX
Cell Cycle: G1/S Checkpoint Regulation	6.97E+00	2.94E-01	-1.5	CCNE1,CCNE2,CDC25A,CDC34,CDK2,CDK6,CDKN2D,E2F1,E2F2,E2F3,E2F7,E2F8,GNL3,HDAC2,PA2G4,PAK1IP1,RBL1,SKP2,SUV39H1,TFDP1
Epithelial Adherens Junction Signaling	6.95E+00	2.09E-01	NaN	ACTB,ACTG1,ACTN1,ACTN4,ACTR2,ACTR3,CDH2,CLIP1,JUP,KRAS,NECTIN2,NOTCH3,NRAS,PARD3,RAC1,RALA,RAP2A,RAP2B,SNAI1,SNAI2,SSX2IP,TCF3,TUBA1B,TUBA1C,TUBB,TUBB3,TUBB6,TUBG1,VAV2,VCL,WASF1,YES1,ZYX
Molecular Mechanisms of Cancer	6.37E+00	1.46E-01	NaN	ARHGEF4,ARHGEF5,AURKA,BCL2L11,BMP1,BMP8A,BRCA1,CCNE1,CCNE2,CDC25A,CDC25C,CDK1,CDK12,CDK16,CDK2,CDK6,CDK8,CDKN2D,CHEK1,CHEK2,E2F1,E2F2,E2F3,E2F7,E2F8,ELK1,FADD,FANCD2,GNA12,GNA13,GNAI3,GNG4,GSK3A,HAT1,HDAC2,HIF1A,IRS1,ITGA11,ITGA5,ITGAV,ITGB1,ITGB5,ITGB8,KRAS,NBN,NRAS,PA2G4,PAK1,PAK2,PLCB3,PRKDC,PTPN11,RAC1,RALA,RALBP1,RAP2A,RAP2B,RBL1,RHOF,RHOV,RND3,SHC1,SUV39H1,TCF3,TFDP1
Protein Ubiquitination Pathway	6.32E+00	1.67E-01	NaN	AMFR,ANAPC1,BRCA1,CDC20,CDC34,CUL2,DNAJA1,DNAJB11,DNAJB6,DNAJC10,DNAJC13,DNAJC2,DNAJC22,DNAJC9,HSP90AA1,HSP90B1,HSPA13,HSPA1A/HSPA1B,HSPD1,HSPH1,PSMB2,PSMC1,PSMD1,PSMD11,PSMD12,PSMD2,PSMD3,PSMD7,SASS6,SKP2,THOP1,UBE2C,UBE2E3,UBE2H,UBE2K,UBE2R2,UBE2S,UBE2T,UBE2V1,UBE2V2,UCHL3,USP1,USP14,USP31,USP32,USP42
Regulation of Cellular Mechanics by Calpain Protease	6.14E+00	2.47E-01	1.897	ACTN1,ACTN4,CCNA2,CCNE1,CCNE2,CDK1,CDK2,CDK6,CNGB1,ITGA11,ITGA5,ITGAV,ITGB1,ITGB5,ITGB8,KRAS,NRAS,PXN,RALA,RAP2A,RAP2B,VCL
Rac Signaling	5.73E+00	2.03E-01	4.796	ACTR2,ACTR3,CDK5R1,CFL1,ELK1,IQGAP3,ITGA11,ITGA5,ITGAV,ITGB1,ITGB5,ITGB8,KRAS,LIMK1,NCKAP1,NRAS,PAK1,PAK2,PARD3,PIP4K2A,PIP4K2C,PLD1,RAC1,RALA,RAP2A,RAP2B,RPS6KB1,WASF1
Germ Cell-Sertoli Cell Junction Signaling	5.65E+00	1.87E-01	NaN	ACTB,ACTG1,ACTN1,ACTN4,CDH2,CFL1,ITGB1,JUP,KRAS,LIMK1,MAP3K10,MTMR2,NECTIN2,NRAS,PAK1,PAK2,PXN,RAC1,RALA,RAP2A,RAP2B,RHOF,RHOV,RND3,TUBA1B,TUBA1C,TUBB,TUBB3,TUBB6,TUBG1,VCL,ZYX
Ephrin Receptor Signaling	5.37E+00	1.74E-01	4.796	ACTR2,ACTR3,ADAM10,CFL1,EFNA3,EPHA1,EPHB2,EPHB4,GNA12,GNA13,GNAI3,GNG4,GRIN2D,ITGA11,ITGA5,ITGAV,ITGB1,ITGB5,ITGB8,KRAS,LIMK1,MAP4K4,NGEF,NRAS,PAK1,PAK2,PGF,PTPN11,PXN,RAC1,RALA,RAP2A,RAP2B,SHC1,VEGFC
Actin Nucleation by ARP-WASP Complex	5.21E+00	2.26E-01	3.638	ACTR2,ACTR3,GNA12,ITGA11,ITGA5,ITGAV,ITGB1,ITGB5,ITGB8,KRAS,NRAS,PPP1R12A,RAC1,RALA,RAP2A,RAP2B,RHOF,RHOV,RND3,VASP,WASF1
Pancreatic Adenocarcinoma Signaling	5.01E+00	1.98E-01	2.714	BIRC5,BRCA2,CCNE1,CCNE2,CDK2,E2F1,E2F2,E2F3,E2F7,E2F8,ELK1,HDAC2,KRAS,PA2G4,PGF,PLD1,RAC1,RAD51,RALA,RALBP1,RBL1,STAT1,SUV39H1,TFDP1,VEGFC
Actin Cytoskeleton Signaling	4.94E+00	1.59E-01	4.596	ACTB,ACTG1,ACTN1,ACTN4,ACTR2,ACTR3,ARHGEF4,CFL1,DIAPH3,FGF11,FN1,GNA12,GNA13,IQGAP3,ITGA11,ITGA5,ITGAV,ITGB1,ITGB5,ITGB8,KRAS,LIMK1,MYLK2,NCKAP1,NRAS,PAK1,PAK2,PFN2,PPP1R12A,PXN,RAC1,RALA,RAP2A,RAP2B,SHC1,TRIO,VAV2,VCL,WASF1
Oncostatin M Signaling	4.89E+00	3.02E-01	3.606	ELK1,KRAS,MMP1,MMP3,MT2A,NRAS,OSMR,PLAU,RALA,RAP2A,RAP2B,SHC1,STAT1
UDP-N-acetyl-D-glucosamine Biosynthesis II	4.82E+00	8.33E-01	2.236	GFPT1,GFPT2,GNPNAT1,PGM3,UAP1
Integrin Signaling	4.80E+00	1.64E-01	4.95	ACTB,ACTG1,ACTN1,ACTN4,ACTR2,ACTR3,ARF6,ASAP1,BCAR3,ITGA11,ITGA5,ITGAV,ITGB1,ITGB5,ITGB8,KRAS,MYLK2,NRAS,PAK1,PAK2,PFN2,PPP1R12A,PXN,RAC1,RALA,RAP2A,RAP2B,RHOF,RHOV,RND3,SHC1,TSPAN5,VASP,VCL,ZYX

Axonal Guidance Signaling	4.76E+00	1.30E-01	NaN	ACTR2,ACTR3,ADAM10,ADAM12,ADAM17,ADAM32,ADAM9,ADAMTS12,ADAMTS2,ADAMTS4,ADAMTS5,ADAMTS6,BMP1,BMP8A,CFL1,EFNA3,EPHA1,EPHB2,EPHB4,GNA12,GNA13,GNAI3,GNG4,ITGA11,ITGA5,ITGAV,ITGB1,ITGB5,ITGB8,KRAS,LIMK1,MMP1,MMP11,MMP12,MMP14,MMP3,NGEF,NRAS,PAK1,PAK2,PFN2,PGF,PLCB3,PLCD1,PLCD3,PLXNA1,PTPN11,PXN,RAC1,RALA,RAP2A,RAP2B,SEMA3A,SEMA3C,SEMA4B,SEMA7A,SHC1,SRGAP1,TUBA1B,TUBA1C,TUBB,TUBB3,TUBB6,TUBG1,VASP,VEGFC
Paxillin Signaling	4.67E+00	2.04E-01	3.771	ACTB,ACTG1,ACTN1,ACTN4,ARF6,ITGA11,ITGA5,ITGAV,ITGB1,ITGB5,ITGB8,KRAS,NRAS,PAK1,PAK2,PTPN12,PXN,RAC1,RALA,RAP2A,RAP2B,VCL
RhoA Signaling	4.65E+00	1.94E-01	3.13	ACTB,ACTG1,ACTR2,ACTR3,ANLN,CDC42EP2,CFL1,EPHA1,GNA12,GNA13,LIMK1,MYLK2,NGEF,PFN2,PIP4K2A,PIP4K2C,PLD1,PLXNA1,PPP1R12A,RHPN2,RND3,RTKN,SEPTIN11,WASF1
Ferroptosis Signaling Pathway	4.53E+00	1.90E-01	0.408	ANGPTL4,ARF6,CARS1,FANCD2,GCH1,GCLC,GSS,H2AX,H2AZ1,H2BC8,KRAS,NRAS,PRKAA2,PRKAB2,RALA,RAP2A,RAP2B,RBL1,SLC38A1,SLC39A14,SLC3A2,SLC7A11,TFRC,TXNRD1
NER (Nucleotide Excision Repair, Enhanced Pathway)	4.49E+00	2.04E-01	3	CHAF1A,CHAF1B,DNA2,GTF2H3,H4C8,H4C9,LIG1,PCNA,POLA2,POLD1,POLD2,POLE,POLE2,PRIM1,PRIM2,RAD23B,RFC2,RFC3,RFC4,RFC5,TOP2A
Signaling by Rho Family GTPases	4.38E+00	1.49E-01	4.642	ACTB,ACTG1,ACTR2,ACTR3,ARHGEF4,ARHGEF5,CDC42EP2,CDH2,CDH24,CDH3,CFL1,CLIP1,DIAPH3,ELK1,GNA12,GNA13,GNAI3,GNG4,ITGA11,ITGA5,ITGAV,ITGB1,ITGB5,ITGB8,LIMK1,MAP3K10,PAK1,PAK2,PARD3,PIP4K2A,PIP4K2C,PLD1,PPP1R12A,RAC1,RHOF,RHOV,RND3,SEPTIN11,STMN1,WASF1
Regulation of Actin-based Motility by Rho	4.17E+00	1.90E-01	3.638	ACTB,ACTR2,ACTR3,CFL1,ITGA11,ITGA5,ITGAV,ITGB1,ITGB5,ITGB8,LIMK1,PAK1,PAK2,PFN2,PIP4K2A,PIP4K2C,PPP1R12A,RAC1,RHOF,RHOV,RND3,WASF1
Cell Cycle Regulation by BTG Family Proteins	4.15E+00	2.97E-01	3	CCNE1,CCNE2,CDK2,E2F1,E2F2,E2F3,E2F7,E2F8,HOXB9,NOCT,PPP2R1B
Sumoylation Pathway	4.00E+00	1.94E-01	-1.807	DNMT3A,HDAC2,KDM1A,PCNA,RAC1,RANGAP1,RCC1,RFC2,RFC3,RFC4,RFC5,RHOF,RHOV,RND3,SAE1,SENP1,SENP2,SENP5,SERBP1,TDG
Sertoli Cell-Sertoli Cell Junction Signaling	3.96E+00	1.55E-01	NaN	ACTB,ACTG1,ACTN1,ACTN4,CLDN14,ELK1,GSK3A,ITGA11,ITGA5,ITGAV,ITGB1,ITGB5,ITGB8,JUP,KRAS,MAP3K10,MTMR2,NECTIN2,NOS1,NRAS,RAC1,RALA,RAP2A,RAP2B,SPTBN2,TUBA1B,TUBA1C,TUBB,TUBB3,TUBB6,TUBG1,VCL
Osteoarthritis Pathway	3.94E+00	1.50E-01	2.785	ACAN,ADAMTS4,ADAMTS5,CASP2,CCN4,DKK1,FADD,FN1,GREM1,H19,HIF1A,IL1R2,IL1RAP,ITGA11,ITGA5,ITGAV,ITGB1,ITGB5,ITGB8,MMP1,MMP12,MMP3,NAMPT,PGF,PPARD,PRKAA2,PRKAB2,PTHLH,RAC1,RUNX2,S100A8,S100A9,SPHK1,TCF3,VEGFC
tRNA Charging	3.92E+00	2.82E-01	3.317	CARS1,DARS2,FARSB,GARS1,IARS1,MARS1,NARS1,RARS1,TARS1,VARS1,YARS2
Tetrahydrofolate Salvage from 5,10-methenyltetrahydrofolate	3.79E+00	8.00E-01	2	GART,MTHFD1,MTHFD1L,MTHFD2
Bladder Cancer Signaling	3.72E+00	1.81E-01	2.449	E2F1,E2F2,E2F3,FGF11,HDAC2,KRAS,MMP1,MMP11,MMP12,MMP14,MMP3,NRAS,PA2G4,PGF,RALA,RAP2A,RAP2B,RBL1,SUV39H1,TFDP1,VEGFC
Neuregulin Signaling	3.66E+00	1.79E-01	3.051	ADAM17,CDK5R1,ELK1,EREG,ERRFI1,HSP90AA1,HSP90B1,ITGA11,ITGA5,ITGAV,ITGB1,ITGB5,ITGB8,KRAS,NRAS,PTPN11,RALA,RAP2A,RAP2B,RPS6KB1,SHC1
DNA damage-induced 14-3-3œÉ Signaling	3.46E+00	3.68E-01	NaN	BRCA1,CCNB1,CCNB2,CCNE1,CCNE2,CDK1,CDK2
DNA Double-Strand Break Repair by Homologous Recombination	3.44E+00	4.29E-01	NaN	BRCA1,BRCA2,GEN1,LIG1,NBN,RAD51
Glutathione Biosynthesis	3.34E+00	1.00E+00	NaN	GCLC,GCLM,GSS
Coronavirus Replication Pathway	3.32E+00	2.44E-01	3.317	ARCN1,COPB2,COPG1,COPG2,CTSL,GSK3A,TUBA1B,TUBA1C,TUBB,TUBB3,TUBB6
GADD45 Signaling	3.30E+00	3.50E-01	NaN	BRCA1,CCNB1,CCNE1,CCNE2,CDK1,CDK2,PCNA
VEGF Signaling	3.29E+00	1.82E-01	3	ACTB,ACTG1,ACTN1,ACTN4,EIF2S1,EIF2S2,HIF1A,KRAS,NRAS,PGF,PTPN11,PXN,RALA,RAP2A,RAP2B,SHC1,VCL,VEGFC
Role of Tissue Factor in Cancer	3.28E+00	1.72E-01	NaN	CFL1,GNA12,GNA13,ITGAV,ITGB1,KRAS,LIMK1,MMP1,NRAS,PAK1,PLAUR,PTPN11,RAC1,RALA,RAP2A,RAP2B,RPS6KA4,RPS6KB1,VEGFC,YES1
RhoGDI Signaling	3.28E+00	1.44E-01	-3.962	ACTB,ACTG1,ACTR2,ACTR3,ARHGEF4,ARHGEF5,CDH2,CDH24,CDH3,CFL1,GNA12,GNA13,GNAI3,GNG4,ITGA11,ITGA5,ITGAV,ITGB1,ITGB5,ITGB8,LIMK1,PAK1,PAK2,PIP4K2A,PIP4K2C,PPP1R12A,RAC1,RHOF,RHOV,RND3,WASF1
FAK Signaling	3.23E+00	1.71E-01	NaN	ACTB,ACTG1,ASAP1,HMMR,ITGA11,ITGA5,ITGAV,ITGB1,ITGB5,ITGB8,KRAS,NRAS,PAK1,PAK2,PXN,RAC1,RALA,RAP2A,RAP2B,VCL
ILK Signaling	3.22E+00	1.46E-01	3.674	ACTB,ACTG1,ACTN1,ACTN4,CFL1,DSP,FBLIM1,FLNC,FN1,GSK3A,HIF1A,IRS1,ITGB1,ITGB5,ITGB8,KRT18,PGF,PPP1R12A,PPP2R1B,PXN,RAC1,RHOF,RHOV,RND3,RPS6KA4,SNAI1,SNAI2,VCL,VEGFC
Senescence Pathway	3.13E+00	1.31E-01	0	ANAPC1,ANAPC7,CCNB1,CCNB2,CCNE1,CCNE2,CDC25A,CDC25C,CDC27,CDK1,CDK2,CDK6,CGAS,CHEK1,CHEK2,DLD,E2F1,E2F2,E2F3,E2F7,E2F8,EED,EIF4EBP1,EZH2,ING1,KRAS,MAPK6,NBN,NRAS,PCGF6,PDK3,PHF19,PPP2R1B,RALA,RAP2A,RAP2B,RBL1,RPS6KA4,SERPINE1
Polyamine Regulation in Colon Cancer	3.13E+00	2.10E-01	NaN	AZIN1,KRAS,MXD1,PSMB2,PSMC1,PSMD1,PSMD11,PSMD12,PSMD2,PSMD3,PSMD7,PSME3,PSME4
Agrin Interactions at Neuromuscular Junction	3.11E+00	2.00E-01	3.464	ACTB,ACTG1,ITGB1,KRAS,LAMB1,LAMC1,NRAS,PAK1,PAK2,PXN,RAC1,RALA,RAP2A,RAP2B
Purine Nucleotides De Novo Biosynthesis II	3.08E+00	4.55E-01	2.236	ATIC,GART,GMPS,PAICS,PPAT
Prostate Cancer Signaling	3.06E+00	1.70E-01	NaN	CCNE1,CCNE2,CDK2,E2F1,E2F2,E2F3,HDAC2,HSP90AA1,HSP90B1,KRAS,NRAS,PA2G4,RALA,RAP2A,RAP2B,RBL1,SRD5A1,SUV39H1,TFDP1
ERK5 Signaling	2.98E+00	1.94E-01	3.742	FOSL1,GNA12,GNA13,KRAS,NRAS,PTPN11,RALA,RAP2A,RAP2B,RPS6KA4,RPS6KB1,YWHAG,YWHAH,YWHAZ
p53 Signaling	2.91E+00	1.73E-01	0	BIRC5,BRCA1,CCNK,CDK2,CHEK1,CHEK2,E2F1,GNL3,HIF1A,PCNA,PERP,PRKDC,SERPINB5,SNAI2,TIGAR,TOPBP1,WT1
HIF1Œ± Signaling	2.87E+00	1.39E-01	3.78	ADM,CCNG2,CUL2,EGLN3,EIF4EBP1,GPI,HIF1A,HK2,HSP90AA1,HSPA1A/HSPA1B,KDM1A,KRAS,MMP1,MMP11,MMP12,MMP14,MMP3,NRAS,PGF,PKM,RAC1,RALA,RAP2A,RAP2B,RPS6KB1,SERPINE1,SLC2A1,SLC2A5,VEGFC
Pyridoxal 5'-phosphate Salvage Pathway	2.86E+00	1.97E-01	3.606	CDK1,CDK2,CDK6,CDK8,IRAK1,LIMK1,MAPK6,NEK2,PAK1,PAK2,PLK1,PRKAA2,TTK
PAK Signaling	2.78E+00	1.61E-01	3.207	CFL1,ITGA11,ITGA5,ITGAV,ITGB1,ITGB5,ITGB8,KRAS,LIMK1,NRAS,PAK1,PAK1IP1,PAK2,PXN,RAC1,RALA,RAP2A,RAP2B,SHC1
14-3-3-mediated Signaling	2.78E+00	1.57E-01	2.496	ELK1,GSK3A,KRAS,NRAS,PLCB3,PLCD1,PLCD3,RALA,RAP2A,RAP2B,SRPK2,TUBA1B,TUBA1C,TUBB,TUBB3,TUBB6,TUBG1,YWHAG,YWHAH,YWHAZ

Clathrin-mediated Endocytosis Signaling	2.74E+00	1.40E-01	NaN	ACTB,ACTG1,ACTR2,ACTR3,AP2A1,AP2M1,ARF6,CD2AP,CLTCL1,CSNK2A1,DNM1L,EPHB2,FGF11,ITGA5,ITGB1,ITGB5,ITGB8,MYO1E,PGF,PICALM,RAC1,S100A8,SH3GL1,SNX9,STAM,TFRC,VEGFC
UDP-N-acetyl-D-galactosamine Biosynthesis II	2.69E+00	3.85E-01	2.236	GNPNAT1,GPI,HK2,PGM3,UAP1
ERK/MAPK Signaling	2.68E+00	1.36E-01	2.132	DUSP4,EIF4EBP1,ELK1,H3C14,ITGA11,ITGA5,ITGAV,ITGB1,ITGB5,ITGB8,KRAS,NRAS,PAK1,PAK2,PPP1CC,PPP1R12A,PPP2R1B,PXN,RAC1,RALA,RAP2A,RAP2B,RPS6KA4,SHC1,STAT1,VRK2,YWHAG,YWHAH,YWHAZ
Inhibition of Matrix Metalloproteases	2.63E+00	2.31E-01	-2.333	ADAM10,ADAM12,ADAM17,MMP1,MMP11,MMP12,MMP14,MMP3,THBS2
Virus Entry via Endocytic Pathways	2.62E+00	1.63E-01	NaN	ACTB,ACTG1,AP2A1,AP2M1,CLTCL1,FLNC,ITGA5,ITGB1,ITGB5,ITGB8,KRAS,NRAS,RAC1,RALA,RAP2A,RAP2B,TFRC
IGF-1 Signaling	2.62E+00	1.63E-01	3.051	CSNK2A1,ELK1,IGFBP3,IRS1,KRAS,NRAS,PTPN11,PXN,RALA,RAP2A,RAP2B,RPS6KB1,SHC1,SOCS4,YWHAG,YWHAH,YWHAZ
Semaphorin Signaling in Neurons	2.61E+00	1.94E-01	NaN	CFL1,ITGB1,LIMK1,PAK1,PAK2,PLXNA1,RAC1,RHOF,RHOV,RND3,SEMA3A,SEMA7A
Ephrin A Signaling	2.58E+00	2.13E-01	NaN	ADAM10,CFL1,EFNA3,EPHA1,LIMK1,NGEF,PAK1,PTPN11,RAC1,VAV2
Glycolysis I	2.55E+00	2.69E-01	2.646	ALDOA,ENO1,ENO2,GAPDH,GPI,PFKP,PKM
Glioblastoma Multiforme Signaling	2.52E+00	1.40E-01	3	CCNE1,CCNE2,CDK2,CDK6,E2F1,E2F2,E2F3,E2F7,E2F8,KRAS,NRAS,PLCB3,PLCD1,PLCD3,RAC1,RALA,RAP2A,RAP2B,RHOF,RHOV,RND3,RPS6KB1,SHC1,TCF3
Folate Transformations I	2.49E+00	4.44E-01	2	MTHFD1,MTHFD1L,MTHFD2,SHMT2
FAT10 Signaling Pathway	2.49E+00	1.96E-01	NaN	PSMB2,PSMC1,PSMD1,PSMD11,PSMD12,PSMD2,PSMD3,PSMD7,PSME3,PSME4,UBA6
Salvage Pathways of Pyrimidine Ribonucleotides	2.49E+00	1.63E-01	4	AK4,APOBEC3B,CDK1,CDK2,CDK6,CDK8,IRAK1,LIMK1,MAPK6,NEK2,PAK1,PAK2,PLK1,PRKAA2,TTK,UCK2
Chronic Myeloid Leukemia Signaling	2.49E+00	1.59E-01	NaN	CDK6,E2F1,E2F2,E2F3,E2F7,E2F8,HDAC2,KRAS,NRAS,PA2G4,PTPN11,RALA,RAP2A,RAP2B,RBL1,SUV39H1,TFDP1
Glioma Invasiveness Signaling	2.45E+00	1.78E-01	3.464	HMMR,ITGAV,KRAS,NRAS,PLAU,PLAUR,RAC1,RALA,RAP2A,RAP2B,RHOF,RHOV,RND3
Coronavirus Pathogenesis Pathway	2.42E+00	1.33E-01	-2.294	ADAM17,ADAM9,BCL2L11,CCNE1,CCNE2,CDK2,CTSL,E2F1,E2F2,E2F3,E2F7,E2F8,ELK1,HDAC2,HIF1A,KPNB1,OAS1,OAS3,PA2G4,PTGES2,RBL1,SERPINE1,SIGMAR1,STAT1,SUV39H1,TFDP1,TOMM70
Hypoxia Signaling in the Cardiovascular System	2.40E+00	1.76E-01	NaN	CDC34,HIF1A,HSP90AA1,HSP90B1,UBE2C,UBE2E3,UBE2H,UBE2K,UBE2R2,UBE2S,UBE2T,UBE2V1,UBE2V2
dTMP De Novo Biosynthesis	2.39E+00	6.00E-01	NaN	DHFR,SHMT2,TYMS
Folate Polyglutamylation	2.39E+00	6.00E-01	NaN	MTHFD1,MTHFD1L,SHMT2
DNA Methylation and Transcriptional Repression Signaling	2.36E+00	2.29E-01	NaN	DNMT1,DNMT3A,DNMT3B,H4C8,H4C9,HDAC2,MTA2,SAP30
Caveolar-mediated Endocytosis Signaling	2.35E+00	1.73E-01	NaN	ACTB,ACTG1,ARCN1,COPB2,COPG1,COPG2,FLNC,ITGA11,ITGA5,ITGAV,ITGB1,ITGB5,ITGB8
Ovarian Cancer Signaling	2.31E+00	1.39E-01	2.828	BRCA1,BRCA2,E2F1,E2F2,E2F3,HDAC2,KRAS,MSH2,MSH6,NRAS,PA2G4,PGF,RAD51,RALA,RAP2A,RAP2B,RBL1,RPS6KB1,SUV39H1,TCF3,TFDP1,VEGFC
Macropinocytosis Signaling	2.30E+00	1.71E-01	3	ACTN4,ARF6,ITGA5,ITGB1,ITGB5,ITGB8,KRAS,NRAS,PAK1,RAC1,RALA,RAP2A,RAP2B
Regulation of eIF4 and p70S6K Signaling	2.26E+00	1.34E-01	2.714	AGO2,EIF2S1,EIF2S2,EIF3B,EIF3J,EIF4A3,EIF4EBP1,EIF4G1,IRS1,ITGA11,ITGA5,ITGAV,ITGB1,ITGB5,ITGB8,KRAS,NRAS,PAIP1,PPP2R1B,RALA,RAP2A,RAP2B,RPS6KB1,SHC1
Tumor Microenvironment Pathway	2.26E+00	1.34E-01	4.796	COL1A1,COL1A2,COL3A1,FGF11,FN1,HIF1A,ITGA5,KRAS,MMP1,MMP11,MMP12,MMP14,MMP3,NRAS,PGF,PLAU,RAC1,RALA,RAP2A,RAP2B,SLC16A1,SLC2A1,TNC,VEGFC
PI3K/AKT Signaling	2.25E+00	1.31E-01	2.668	EIF4EBP1,GSK3A,HSP90AA1,HSP90B1,IL12RB2,IL17RD,IL1R2,IL20RB,ITGA11,ITGA5,ITGAV,ITGB1,ITGB5,ITGB8,KRAS,NRAS,PPP2R1B,RALA,RAP2A,RAP2B,RPS6KB1,SHC1,SYNJ2,YWHAG,YWHAH,YWHAZ
Uridine-5'-phosphate Biosynthesis	2.23E+00	1.00E+00	NaN	CAD,UMPS
Glioma Signaling	2.17E+00	1.45E-01	2.449	CDK6,CDKN2D,E2F1,E2F2,E2F3,E2F7,E2F8,HDAC2,KRAS,NRAS,PA2G4,RALA,RAP2A,RAP2B,RBL1,SHC1,SUV39H1,TFDP1
HOTAIR Regulatory Pathway	2.15E+00	1.35E-01	4.025	AGO2,COL1A1,COL1A2,COL3A1,DNMT3B,EED,EZH2,FOXM1,H3C14,HDAC2,JARID2,KDM1A,MMP1,MMP11,MMP12,MMP14,MMP3,MTF2,SNAI2,SUZ12,TCF3,TWIST1
D-myo-inositol-5-phosphate Metabolism	2.14E+00	1.30E-01	4.264	CDC25A,CDC25C,DUSP14,DUSP4,HACD2,MTMR2,NOCT,NUDT1,NUDT15,PAWR,PGAM5,PIP4K2A,PIP4K2C,PLCB3,PLCD1,PLCD3,PPP1CC,PPP1R12A,PPP2R1B,PTPN11,PTPN12,PTPRH,PTPRN,SET,WBP11
Aldosterone Signaling in Epithelial Cells	2.12E+00	1.34E-01	1.633	DNAJA1,DNAJB11,DNAJB6,DNAJC10,DNAJC13,DNAJC2,DNAJC22,DNAJC9,HSP90AA1,HSP90B1,HSPA13,HSPA1A/HSPA1B,HSPD1,HSPH1,KCNMB4,KRAS,PIP4K2A,PIP4K2C,PLCB3,PLCD1,PLCD3,SASS6
Renal Cell Carcinoma Signaling	2.11E+00	1.63E-01	3	CUL2,EGLN3,HIF1A,KRAS,NRAS,PAK1,PAK2,PTPN11,RAC1,RALA,RAP2A,RAP2B,SLC2A1
Reelin Signaling in Neurons	2.10E+00	1.43E-01	3	ACTR2,ACTR3,ARHGEF4,ARHGEF5,CDH2,CDK5R1,CFL1,GRIN2D,ITGA5,ITGB1,LIMK1,MAP3K10,PAFAH1B2,PAFAH1B3,PDK3,RAC1,WASF1,YES1
Ephrin B Signaling	2.07E+00	1.67E-01	3.162	CFL1,EPHB2,EPHB4,GNA12,GNA13,GNAI3,GNG4,LIMK1,PAK1,PXN,RAC1,VAV2
Natural Killer Cell Signaling	1.98E+00	1.26E-01	2.2	CFL1,COL1A1,COL1A2,COL3A1,COL5A3,HSPA1A/HSPA1B,IL12RB2,ITGB1,KIR2DL4,KRAS,LIMK1,MAP3K10,NECTIN2,NRAS,PAK1,PAK2,PTPN11,PVR,PXN,RAC1,RALA,RAP2A,RAP2B,ULBP2,VAV2
Cleavage and Polyadenylation of Pre-mRNA	1.98E+00	3.33E-01	NaN	CPSF2,CPSF3,CSTF2,CSTF3
BER pathway	1.98E+00	3.33E-01	NaN	FEN1,LIG1,PCNA,POLE
PTEN Signaling	1.95E+00	1.33E-01	-2.5	BCL2L11,CSNK2A1,GSK3A,ITGA11,ITGA5,ITGAV,ITGB1,ITGB5,ITGB8,KRAS,MAST2,NRAS,RAC1,RALA,RAP2A,RAP2B,RPS6KB1,SHC1,SYNJ2,YWHAH
Spliceosomal Cycle	1.94E+00	1.84E-01	3	CTNNBL1,DHX15,EFTUD2,EIF4A3,PLRG1,PRPF19,SF3B4,U2AF1/U2AF1L5,U2AF2
IL-8 Signaling	1.93E+00	1.23E-01	4.899	ANGPT2,EIF4EBP1,GNA12,GNA13,GNAI3,GNG4,IRAK1,ITGAV,KRAS,LASP1,LIMK1,MAP4K4,NRAS,PAK2,PGF,PLD1,RAC1,RALA,RAP2A,RAP2B,RHOF,RHOV,RND3,RPS6KB1,VASP,VEGFC
Mechanisms of Viral Exit from Host Cells	1.93E+00	1.95E-01	NaN	ACTB,ACTG1,LMNB1,LMNB2,SH3GL1,SNF8,VPS37C,XPO1
Apelin Liver Signaling Pathway	1.91E+00	2.31E-01	2.449	COL1A1,COL1A2,COL3A1,COL5A3,GSK3A,IRS1
HGF Signaling	1.90E+00	1.36E-01	3.742	CDK2,ELK1,ITGA11,ITGA5,ITGAV,ITGB1,ITGB5,ITGB8,KRAS,MAP3K10,NRAS,PAK1,PTPN11,PXN,RAC1,RALA,RAP2A,RAP2B

Non-Small Cell Lung Cancer Signaling	1.90E+00	1.49E-01	2.449	CDK6,E2F1,E2F2,E2F3,HDAC2,KRAS,NRAS,PA2G4,RALA,RAP2A,RAP2B,RBL1,SUV39H1,TFDP1
Superpathway of Serine and Glycine Biosynthesis I	1.90E+00	4.29E-01	NaN	PSAT1,PSPH,SHMT2
Superpathway of Inositol Phosphate Compounds	1.90E+00	1.20E-01	4.6	CDC25A,CDC25C,DUSP14,DUSP4,HACD2,IPMK,IPPK,MTMR2,NOCT,NUDT1,NUDT15,PAWR,PGAM5,PIP4K2A,PIP4K2C,PLCB3,PLCD1,PLCD3,PPP1CC,PPP1R12A,PPP2R1B,PTPN11,PTPN12,PTPRH,PTPRN,SET,SYNJ2,WBP11
HIPPO signaling	1.89E+00	1.53E-01	-2.121	CSNK1E,FRMD6,ITCH,LLGL1,PARD3,PPP1CC,PPP1R12A,PPP2R1B,SKP2,TEAD4,YWHAG,YWHAH,YWHAZ
Role of JAK2 in Hormone-like Cytokine Signaling	1.87E+00	2.06E-01	NaN	HLTF,IRS1,PTPN11,SH2B2,SHC1,SOCS4,STAT1
Small Cell Lung Cancer Signaling	1.82E+00	1.46E-01	1	CCNE1,CCNE2,CDK2,CDK6,E2F1,E2F2,E2F3,HDAC2,NOS1,PA2G4,RBL1,SKP2,SUV39H1,TFDP1
Synaptogenesis Signaling Pathway	1.80E+00	1.12E-01	4.564	ACTR2,ACTR3,AP2A1,AP2M1,CDH2,CDH24,CDH3,CFL1,EFNA3,EIF4EBP1,EPHA1,EPHB2,EPHB4,GRIN2D,GRM8,KRAS,LIMK1,MARCKS,NLGN2,NLGN4X,NRAS,PAK1,RAC1,RALA,RAP2A,RAP2B,RPS6KB1,SHC1,STX1A,STXBP5,SYT5,THBS2,WASF1,YES1,YKT6
5-aminoimidazole Ribonucleotide Biosynthesis I	1.77E+00	6.67E-01	NaN	GART,PPAT
Inosine-5'-phosphate Biosynthesis II	1.77E+00	6.67E-01	NaN	ATIC,PAICS
1D-myo-inositol Hexakisphosphate Biosynthesis V (from Ins(1,3,4)P3)	1.77E+00	6.67E-01	NaN	IPMK,IPPK
UVA-Induced MAPK Signaling	1.75E+00	1.43E-01	2.53	KRAS,NRAS,PARP12,PARP2,PLCB3,PLCD1,PLCD3,RALA,RAP2A,RAP2B,RPS6KA4,RPS6KB1,SMPD4,STAT1
GP6 Signaling Pathway	1.75E+00	1.34E-01	3	ADAM10,COL11A1,COL12A1,COL15A1,COL1A1,COL1A2,COL3A1,COL5A1,COL5A2,COL5A3,COL7A1,GSK3A,LAMB1,LAMC1,LAMC2,RAC1,VAV2
Pyrimidine Ribonucleotides De Novo Biosynthesis	1.74E+00	1.94E-01	2.646	AK4,CAD,CANT1,CTPS1,ENTPD7,NUDT15,UMPS
FLT3 Signaling in Hematopoietic Progenitor Cells	1.72E+00	1.50E-01	2.887	EIF4EBP1,ELK1,KRAS,NRAS,PTPN11,RALA,RAP2A,RAP2B,RPS6KA4,RPS6KB1,SHC1,STAT1
Histidine Degradation III	1.72E+00	3.75E-01	NaN	MTHFD1,MTHFD1L,MTHFD2
Purine Ribonucleosides Degradation to Ribose-1-phosphate	1.72E+00	3.75E-01	NaN	ADA,PGM2,PNP
p70S6K Signaling	1.60E+00	1.29E-01	2.673	GNAI3,IRS1,KRAS,NRAS,PLCB3,PLCD1,PLCD3,PLD1,PPP2R1B,RALA,RAP2A,RAP2B,RPS6KB1,SHC1,YWHAG,YWHAH,YWHAZ
NRF2-mediated Oxidative Stress Response	1.58E+00	1.14E-01	3	ACTB,ACTG1,BACH1,CDC34,DNAJA1,DNAJB11,DNAJB6,DNAJC10,DNAJC13,DNAJC9,FOSL1,GCLC,GCLM,HSP90AA1,HSP90B1,KRAS,NRAS,RALA,RAP2A,RAP2B,SCARB1,SLC35A2,STIP1,TXNRD1,UBE2E3,UBE2K,USP14
BAG2 Signaling Pathway	1.57E+00	1.43E-01	0	HSP90AA1,HSPA1A/HSPA1B,PSMB2,PSMC1,PSMD1,PSMD11,PSMD12,PSMD2,PSMD3,PSMD7,PSME3,PSME4
FcŒ≥ Receptor-mediated Phagocytosis in Macrophages and Monocytes	1.56E+00	1.38E-01	3.606	ACTB,ACTG1,ACTR2,ACTR3,ARF6,PAK1,PLD1,PXN,RAC1,RPS6KB1,VASP,VAV2,YES1
Apelin Cardiac Fibroblast Signaling Pathway	1.55E+00	2.17E-01	-0.447	ANGPT2,PRKAA2,PRKAB2,SERPINE1,SPHK1
Estrogen Receptor Signaling	1.54E+00	1.04E-01	3.244	ATP5F1B,CARM1,CDK8,CFL1,EIF4EBP1,FBXO32,GNA12,GNA13,GNAI3,GNG4,GSK3A,HIF1A,HSP90AA1,HSP90B1,KRAS,LIMK1,MMP1,MMP11,MMP12,MMP14,MMP3,NRAS,NRIP1,PAK1,PCNA,PGF,PLCB3,PLCD1,PLCD3,PPP1R12A,PRKAA2,PRKAB2,PRKDC,RALA,RAP2A,RAP2B,RPS6KB1,RUNX2,SHC1,SNAI1,TBL1XR1,VEGFC
CNTF Signaling	1.54E+00	1.58E-01	3	KRAS,NRAS,PTPN11,RALA,RAP2A,RAP2B,RPS6KA4,RPS6KB1,STAT1
Granzyme B Signaling	1.52E+00	2.50E-01	2	GZMB,LMNB1,LMNB2,PRKDC
CCR3 Signaling in Eosinophils	1.52E+00	1.26E-01	3.162	CCL11,CFL1,GNA12,GNA13,GNAI3,GNG4,KRAS,LIMK1,NRAS,PAK1,PAK2,PLCB3,PPP1R12A,RAC1,RALA,RAP2A,RAP2B
D-myo-inositol (1,4,5,6)-Tetrakisphosphate Biosynthesis	1.50E+00	1.19E-01	4.243	CDC25A,CDC25C,DUSP14,DUSP4,HACD2,IPMK,MTMR2,NOCT,NUDT1,NUDT15,PAWR,PGAM5,PPP1CC,PPP1R12A,PPP2R1B,PTPN11,PTPN12,PTPRH,PTPRN,SET,WBP11
D-myo-inositol (3,4,5,6)-tetrakisphosphate Biosynthesis	1.50E+00	1.19E-01	4.243	CDC25A,CDC25C,DUSP14,DUSP4,HACD2,IPMK,MTMR2,NOCT,NUDT1,NUDT15,PAWR,PGAM5,PPP1CC,PPP1R12A,PPP2R1B,PTPN11,PTPN12,PTPRH,PTPRN,SET,WBP11
Proline Biosynthesis I	1.49E+00	5.00E-01	NaN	ALDH18A1,PYCR1
Oxidized GTP and dGTP Detoxification	1.49E+00	5.00E-01	NaN	NUDT1,RUVBL2
Antiproliferative Role of Somatostatin Receptor 2	1.48E+00	1.43E-01	2.333	ELK1,GAD1,GNG4,KRAS,NOS1,NRAS,PTPN11,RALA,RAP2A,RAP2B,SSTR2
3-phosphoinositide Degradation	1.47E+00	1.16E-01	4.359	CDC25A,CDC25C,DUSP14,DUSP4,HACD2,INPP4B,MTMR2,NOCT,NUDT1,NUDT15,PAWR,PGAM5,PPP1CC,PPP1R12A,PPP2R1B,PTPN11,PTPN12,PTPRH,PTPRN,SET,SYNJ2,WBP11
Breast Cancer Regulation by Stathmin1	1.46E+00	9.80E-02	4.371	ADGRE2,ADGRF4,ADGRG3,ARHGEF4,ARHGEF5,AURKA,AURKB,AVPR1A,BDKRB1,BDKRB2,CDK1,CDK2,CDK6,CELSR3,E2F1,E2F2,E2F3,E2F7,E2F8,FOXM1,GNA13,GNG4,GPR1,GPR157,GPR158,GPR176,GPR180,GPR19,GPR37,GPR37L1,GPR89A/GPR89B,GRM8,HIF1A,HTR1D,KRAS,NRAS,PAK1,PGF,PLCB3,PPP1CC,PPP1R12A,PPP2R1B,RAC1,RALA,RAP2A,RAP2B,RPS6KB1,SHC1,SLC52A2,SSTR2,STMN1,TUBA1B,TUBA1C,TUBB,TUBB3,TUBB6,TUBG1,VEGFC
Calcium Transport I	1.44E+00	3.00E-01	NaN	ATP2A2,ATP2B1,ATP2C1
Aryl Hydrocarbon Receptor Signaling	1.43E+00	1.19E-01	2.5	ALDH18A1,ALDH1L2,ALDH3B2,CCNA2,CCNE1,CCNE2,CDK2,CDK6,CHEK1,CHEK2,DHFR,E2F1,HSP90AA1,HSP90B1,MCM7,NRIP1,RBL1,SLC35A2,TFDP1
mTOR Signaling	1.43E+00	1.13E-01	2.828	EIF3B,EIF3J,EIF4A3,EIF4EBP1,EIF4G1,HIF1A,IRS1,KRAS,NRAS,PGF,PLD1,PPP2R1B,PRKAA2,PRKAB2,RAC1,RALA,RAP2A,RAP2B,RHOF,RHOV,RND3,RPS6KA4,RPS6KB1,VEGFC
Cholecystokinin/Gastrin-mediated Signaling	1.40E+00	1.26E-01	3.742	ELK1,GNA12,GNA13,KRAS,NRAS,PLCB3,PXN,RAC1,RALA,RAP2A,RAP2B,RHOF,RHOV,RND3,SHC1
3-phosphoinositide Biosynthesis	1.40E+00	1.13E-01	4.359	CDC25A,CDC25C,DUSP14,DUSP4,HACD2,IPMK,MTMR2,NOCT,NUDT1,NUDT15,PAWR,PGAM5,PIP4K2A,PIP4K2C,PPP1CC,PPP1R12A,PPP2R1B,PTPN11,PTPN12,PTPRH,PTPRN,SET,WBP11
Inhibition of ARE-Mediated mRNA Degradation Pathway	1.39E+00	1.18E-01	0.905	AGO2,CNOT9,EXOSC2,MAPK6,PPP2R1B,PSMB2,PSMC1,PSMD1,PSMD11,PSMD12,PSMD2,PSMD3,PSMD7,PSME3,PSME4,TNFSF11,YWHAG,YWHAH,YWHAZ
PPARŒ±/RXRŒ± Activation	1.38E+00	1.14E-01	-2.668	ADIPOR2,CAND1,CKAP5,GPD2,HSP90AA1,HSP90B1,IL1R2,IL1RAP,IRS1,ITGB5,KRAS,MAP4K4,NRAS,PLCB3,PLCD1,PLCD3,PRKAA2,PRKAB2,RALA,RAP2A,RAP2B,SHC1
Chemokine Signaling	1.37E+00	1.38E-01	2.111	CCL11,CFL1,GNAI3,KRAS,LIMK1,NRAS,PLCB3,PPP1R12A,RALA,RAP2A,RAP2B
IL-2 Signaling	1.37E+00	1.48E-01	2.828	CSNK2A1,ELK1,KRAS,NRAS,PTPN11,RALA,RAP2A,RAP2B,SHC1
Œ≥-linolenate Biosynthesis II (Animals)	1.35E+00	2.22E-01	2	ACSL3,FADS1,FADS2,SLC27A4
D-myo-inositol (1,4,5)-Trisphosphate Biosynthesis	1.34E+00	1.92E-01	1.342	PIP4K2A,PIP4K2C,PLCB3,PLCD1,PLCD3

Gluconeogenesis I	1.34E+00	1.92E-01	2.236	ALDOA,ENO1,ENO2,GAPDH,GPI
Œ≥-glutamyl Cycle	1.33E+00	2.73E-01	NaN	GCLC,GCLM,GSS
GDP-glucose Biosynthesis	1.33E+00	2.73E-01	NaN	HK2,PGM2,PGM3
HER-2 Signaling in Breast Cancer	1.32E+00	1.12E-01	2.558	ARF6,BCL2L11,BIRC5,CCNE1,CCNE2,COX6B2,ELK1,GSK3A,ITGB1,ITGB5,ITGB8,KPNB1,KRAS,NRAS,PARD3,PARD6G,RALA,RAP2A,RAP2B,RBL1,RPS6KB1,YES1
Endocannabinoid Cancer Inhibition Pathway	1.32E+00	1.19E-01	-0.243	CASP2,CCNE1,CCNE2,GNA13,GNAI3,HIF1A,NOS1,PGF,PRKAA2,PRKAB2,SMPD4,SNAI2,SPTLC1,TCF3,TRIB3,TWIST1,VEGFC
CDK5 Signaling	1.31E+00	1.25E-01	1.732	CDK5R1,ITGB1,KRAS,LAMB1,LAMC1,LAMC2,MAPK6,NRAS,PPP1CC,PPP1R12A,PPP2R1B,RALA,RAP2A,RAP2B

Supplemental Table 2. Pathway Analysis for Differential Gene Expression Heatmap Cluster 2. Pathways involved in the differential expression of genes in heatmap cluster 2 are shown. Ratio refers to the number of differentially regulated genes in the dataset relative to the total number of pathway genes. Z-score refers to the relationship between observed vs. predicted pathway member regulation. A positive Z-score indicates that the observed gene activity positively correlates with predicted pathway member up/down regulation patterns; a negative Z-score indicates anti-correlation.

Table S3. Pathway Analysis for Differential Gene Expression Heatmap Cluster 3

Ingenuity Canonical Pathways	-log(p-value)	Ratio	z-score	Molecules
Antigen Presentation Pathway	1.03E+01	4.10E-01	NaN	CD74,CIITA,HLA-DMA,HLA-DMB,HLA-DOA,HLA-DOB,HLA-DPA1,HLA-DPB1,HLA-DQA1,HLA-DQB1,HLA-DQB2,HLA-DRA,HLA-DRB1,HLA-DRB5,HLA-E,MR1
Th1 and Th2 Activation Pathway	9.15E+00	1.86E-01	NaN	CCR4,CD40LG,HLA-DMA,HLA-DMB,HLA-DOA,HLA-DOB,HLA-DPA1,HLA-DPB1,HLA-DQA1,HLA-DQB1,HLA-DQB2,HLA-DRA,HLA-DRB1,HLA-DRB5,IKZF1,IL12B,IL24,IL33,IL6R,JUN,LTA,NFATC1,NFATC2,NFATC3,PIK3R1,PIK3R6,PRKCQ,PTGDR2,S1PR1,TGFBR2,TGFBR3,TSLP
CREB Signaling in Neurons	8.94E+00	1.16E-01	-7.878	ACKR1,ADCY2,ADCY9,ADGRB3,ADGRF5,ADGRG2,ADRA2A,ADRB2,CACNA1D,CACNA2D2,CACNA2D3,CACNB4,CALCRL,CAMK2D,CAMK4,CCR2,CCR4,CCR6,CCR7,CNR2,CX3CR1,CYSLTR1,EDNRB,FGFR2,GHR,GNAL,GNAO1,GNG7,GPR146,GPR162,GPR20,GPR25,GPR55,GRIA1,GRID1,GRIK3,GRIK4,HGF,ITPR1,ITPR2,MCHR1,NGFR,NMUR1,NPY1R,NTRK2,NTRK3,P2RY12,P2RY13,P2RY14,PIK3R1,PIK3R6,PLCB2,PLCE1,PLCG2,PLCL1,PRKCB,PRKCE,PRKCQ,PTGDR2,PTGER4,PTGIR,PTH1R,RXFP1,S1PR1,S1PR4,SHC3,SSTR1,TGFBR2,TGFBR3,XCR1
Cardiac Hypertrophy Signaling (Enhanced)	8.61E+00	1.19E-01	-7.211	ADCY2,ADCY9,ADRA2A,ADRB2,AKAP13,CACNA1D,CACNA2D2,CACNA2D3,CACNB4,CAMK2D,CAMK4,CD40LG,EDNRB,ENPP6,FGF10,FGF14,FGFR2,GHR,GNG7,HSPB7,IL12B,IL13RA2,IL33,IL5RA,IL6R,IL6ST,ITGA10,ITGA8,ITGA9,ITGAL,ITPR1,ITPR2,JUN,LTA,LTB,MAP3K3,MAPK10,MEF2C,MYOCD,NFATC1,NFATC2,NFATC3,NGFR,PDE1B,PDE1C,PDE7B,PDE8B,PIK3R1,PIK3R6,PLCB2,PLCE1,PLCG2,PLCL1,PRKCB,PRKCE,PRKCQ,RPS6KA5,RYR2,TGFBR2,TGFBR3,TNFSF12,TNFSF13,WNT11,WNT2B
Th2 Pathway	8.39E+00	1.97E-01	-0.905	CCR4,HLA-DMA,HLA-DMB,HLA-DOA,HLA-DOB,HLA-DPA1,HLA-DPB1,HLA-DQA1,HLA-DQB1,HLA-DQB2,HLA-DRA,HLA-DRB1,HLA-DRB5,IKZF1,IL12B,IL24,IL33,JUN,NFATC2,PIK3R1,PIK3R6,PRKCQ,PTGDR2,S1PR1,TGFBR2,TGFBR3,TSLP
B Cell Development	7.53E+00	3.26E-01	NaN	CD79B,HLA-DMA,HLA-DMB,HLA-DOA,HLA-DOB,HLA-DPA1,HLA-DPB1,HLA-DQA1,HLA-DQB1,HLA-DQB2,HLA-DRA,HLA-DRB1,HLA-DRB5,SPN
Neuroinflammation Signaling Pathway	7.23E+00	1.33E-01	-4	AGER,BCL2,CD200R1,CX3CL1,CX3CR1,FOS,GRIA1,HLA-DMA,HLA-DMB,HLA-DOA,HLA-DOB,HLA-DPA1,HLA-DPB1,HLA-DQA1,HLA-DQB1,HLA-DQB2,HLA-DRA,HLA-DRB1,HLA-DRB5,HLA-E,IL12B,IL6R,JUN,KCNJ5,MAPK10,MMP9,MR1,NFATC1,NFATC2,NFATC3,PIK3R1,PIK3R6,PLCG2,S100B,SLC1A2,TGFBR2,TGFBR3,TLR10,TLR2,TLR3,TLR5,TLR7
PD-1, PD-L1 cancer immunotherapy pathway	6.71E+00	1.98E-01	3.638	FBXO38,HLA-DMA,HLA-DMB,HLA-DOA,HLA-DOB,HLA-DPA1,HLA-DPB1,HLA-DQA1,HLA-DQB1,HLA-DQB2,HLA-DRA,HLA-DRB1,HLA-DRB5,HLA-E,IL12B,MR1,NGFR,PIK3R1,PIK3R6,PRKCQ,ZAP70
IL-4 Signaling	6.43E+00	2.07E-01	NaN	FCER2,HLA-DMA,HLA-DMB,HLA-DOA,HLA-DOB,HLA-DPA1,HLA-DPB1,HLA-DQA1,HLA-DQB1,HLA-DQB2,HLA-DRA,HLA-DRB1,HLA-DRB5,NFATC1,NFATC2,NFATC3,NR3C2,PIK3R1,PIK3R6
Protein Kinase A Signaling	6.27E+00	1.17E-01	-2.214	ADCY2,ADCY9,AKAP13,AKAP14,AKAP6,AKAP7,CAMK2D,CAMK4,CNGA4,DUSP1,DUSP16,DUSP26,ENPP6,EPM2A,GNG7,H1-4,ITPR1,ITPR2,MYL3,NFATC1,NFATC2,NFATC3,NGFR,PDE1B,PDE1C,PDE7B,PDE8B,PHKB,PLCB2,PLCE1,PLCG2,PLCL1,PPP1R3C,PRKCB,PRKCE,PRKCQ,PTCH1,PTK2B,PTPN13,PTPN4,PTPRB,PTPRE,PTPRM,PTPRQ,PTPRT,RYR2,TGFBR2
Th1 Pathway	6.26E+00	1.80E-01	-4	CD40LG,HLA-DMA,HLA-DMB,HLA-DOA,HLA-DOB,HLA-DPA1,HLA-DPB1,HLA-DQA1,HLA-DQB1,HLA-DQB2,HLA-DRA,HLA-DRB1,HLA-DRB5,IL12B,IL6R,LTA,NFATC1,NFATC2,NFATC3,PIK3R1,PIK3R6,PRKCQ
Phagosome Formation	5.30E+00	1.54E-01	NaN	CR2,FCAMR,FCER1A,FCER2,ITGA10,ITGA8,ITGA9,ITGAL,PIK3R1,PIK3R6,PLCB2,PLCE1,PLCG2,PLCL1,PRKCB,PRKCE,PRKCQ,SCARA3,TLR10,TLR2,TLR3,TLR5,TLR7
cAMP-mediated signaling	5.19E+00	1.31E-01	-4.315	ADCY2,ADCY9,ADRA2A,ADRB2,AKAP13,AKAP14,AKAP6,AKAP7,CAMK2D,CAMK4,CCR4,CNGA4,CNR2,DUSP1,ENPP6,GNAL,GNAO1,NPY1R,P2RY12,P2RY13,P2RY14,PDE1B,PDE1C,PDE7B,PDE8B,PTGER4,PTGIR,PTH1R,S1PR1,XCR1
Endocannabinoid Neuronal Synapse Pathway	4.88E+00	1.50E-01	-3.3	ADCY2,ADCY9,CACNA1D,CACNA2D2,CACNA2D3,CACNB4,CNR2,DNAH10,DNALI1,GNAL,GNAO1,GNG7,GRIA1,ITPR1,ITPR2,KCNJ5,MAPK10,MGLL,PLCB2,PLCE1,PLCG2,PLCL1
MSP-RON Signaling In Macrophages Pathway	4.81E+00	1.62E-01	2.065	CIITA,F11,FOS,HLA-DMA,HLA-DMB,HLA-DOA,HLA-DOB,HLA-DPA1,HLA-DPB1,HLA-DQA1,HLA-DQB1,HLA-DQB2,HLA-DRA,HLA-DRB1,HLA-DRB5,IL12B,JUN,PIK3R1,PIK3R6
Systemic Lupus Erythematosus In B Cell Signaling Pathway	4.76E+00	1.19E-01	-5.048	BCL2,BLNK,BTK,CAMK4,CD22,CD40LG,CD5,CD79B,FOS,IL12B,IL33,IL6R,IL6ST,JUN,LTA,LTB,NFATC1,NFATC2,NFATC3,PIK3R1,PIK3R6,PLCG2,PRKCB,PRKCE,PRKCQ,RASGRP2,SHC3,SHE,STING1,TLR3,TLR7,TNFSF12,TNFSF13
Complement System	4.74E+00	2.70E-01	-0.816	C3,C4A/C4B,C4BPA,C6,C7,C8B,CFD,CFI,CR2,MASP1
Sperm Motility	4.72E+00	1.22E-01	-3.742	BLK,BMX,BTK,CAMK4,CNGA4,EPHA3,ERBB4,FGFR2,FLT3LG,GNAL,GNAO1,GNG7,GUCY1A2,ITPR1,ITPR2,MUSK,NTRK2,NTRK3,PDE1B,PDE1C,PLCB2,PLCE1,PLCG2,PLCL1,PRKCB,PRKCE,PRKCQ,PTK2B,ROS1,TEK,ZAP70
Crosstalk between Dendritic Cells and Natural Killer Cells	4.58E+00	1.76E-01	-3.873	CAMK2D,CCR7,CD40LG,CD69,CD83,HLA-DRA,HLA-DRB1,HLA-DRB5,HLA-E,IL12B,ITGAL,LTA,LTB,NCR3,TLR3,TLR7
Neuropathic Pain Signaling In Dorsal Horn Neurons	4.57E+00	1.68E-01	-4.123	CAMK2D,CAMK4,FOS,GRIA1,ITPR1,ITPR2,KCNN3,NTRK2,PIK3R1,PIK3R6,PLCB2,PLCE1,PLCG2,PLCL1,PRKCB,PRKCE,PRKCQ
Adrenomedullin signaling pathway	4.57E+00	1.31E-01	-4.315	ADCY2,ADCY9,BCL2,C3,CALCRL,CAMK4,FOS,GUCY1A2,IL33,ITPR1,ITPR2,KCNN3,LTA,MAPK10,PIK3R1,PIK3R6,PLCB2,PLCE1,PLCG2,PLCL1,PTK2B,RAMP2,RAMP3,RXRG,SHC3,SHE
PI3K Signaling in B Lymphocytes	4.56E+00	1.47E-01	-4.359	BLK,BLNK,BTK,C3,CAMK2D,CAMK4,CD79B,CR2,FOS,ITPR1,ITPR2,JUN,NFATC1,NFATC2,NFATC3,PIK3R1,PLCB2,PLCE1,PLCG2,PLCL1,PRKCB
Endothelin-1 Signaling	4.43E+00	1.31E-01	-3.8	ADCY2,ADCY9,CASQ1,EDNRB,FOS,GNAL,GNAO1,GPLD1,GUCY1A2,ITPR1,ITPR2,JUN,MAPK10,PIK3R1,PIK3R6,PLCB2,PLCE1,PLCG2,PLCL1,PLD4,PRKCB,PRKCE,PRKCQ,SHC3,SHE
Cellular Effects of Sildenafil (Viagra)	4.29E+00	1.41E-01	NaN	ADCY2,ADCY9,CACNA1D,CACNA2D2,CACNA2D3,CACNB4,CAMK4,GUCY1A2,ITPR1,ITPR2,KCNN3,MYH11,MYL3,PDE1B,PDE1C,PLCB2,PLCE1,PLCG2,PLCL1,PPP1R12B,SLC4A5

Corticotropin Releasing Hormone Signaling	4.29E+00	1.41E-01	-1.886	ADCY2,ADCY9,CACNA1D,CACNA2D2,CACNA2D3,CACNB4,CAMK4,FOS,GNAO1,GUCY1A2,ITPR1,ITPR2,JUN,KRT1,MEF2C,PLCG2,PRKCB,PRKCE,PRKCQ,PTCH1,UCN3
Breast Cancer Regulation by Stathmin1	4.20E+00	9.29E-02	-6.26	ACKR1,ADGRB3,ADGRF5,ADGRG2,ADRA2A,ADRB2,ANKHD1/ANKHD1-EIF4EBP3,ARHGEF15,ARHGEF18,ARHGEF6,ARHGEF9,CALCRL,CAMK2D,CAMK4,CCR2,CCR4,CCR6,CCR7,CNR2,CX3CR1,CYSLTR1,EDNRB,GNG7,GPR146,GPR162,GPR20,GPR25,GPR55,HGF,JUN,MCHR1,MMP9,NMUR1,NPY1R,P2RY12,P2RY13,P2RY14,PIK3R1,PIK3R6,PLCB2,PPP1R3C,PRKCB,PRKCE,PRKCQ,PTGDR2,PTGER4,PTGIR,PTH1R,RXFP1,S1PR1,S1PR4,SHC3,SSTR1,VEGFD,XCR1
Role of Macrophages, Fibroblasts and Endothelial Cells in Rheumatoid Art	4.09E+00	1.08E-01	NaN	CAMK2D,CAMK4,CEBPA,FOS,FRZB,GNAO1,IL16,IL33,IL6R,IL6ST,JUN,LRP6,LTA,LTB,NFATC1,NFATC2,NFATC3,NGFR,PIK3R1,PIK3R6,PLCB2,PLCE1,PLCG2,PLCL1,PRKCB,PRKCE,PRKCQ,TLR10,TLR2,TLR3,TLR5,TLR7,VEGFD,WNT11,WNT2B
G-Protein Coupled Receptor Signaling	4.03E+00	1.12E-01	NaN	ADCY2,ADCY9,ADRA2A,ADRB2,CAMK2D,CAMK4,CCR4,CNR2,DUSP1,ENPP6,GNAL,GNAO1,NPY1R,P2RY12,P2RY13,P2RY14,PDE1B,PDE1C,PDE7B,PDE8B,PIK3R1,PIK3R6,PLCB2,PRKCB,PRKCE,PTGER4,PTGIR,PTH1R,PTK2B,S1PR1,XCR1
Leukocyte Extravasation Signaling	3.92E+00	1.24E-01	-3.13	ACTN2,ARHGAP6,BMX,BTK,CLDN18,CLDN2,DLC1,ITGAL,JAM2,MAPK10,MMP24,MMP28,MMP9,NCF4,PECAM1,PIK3R1,PIK3R6,PLCG2,PRKCB,PRKCE,PRKCQ,PTK2B,RASSF5,SPN
Role of NFAT in Cardiac Hypertrophy	3.83E+00	1.18E-01	-5	ADCY2,ADCY9,CACNA1D,CACNA2D2,CACNA2D3,CACNB4,CAMK2D,CAMK4,GNG7,IL6ST,ITPR1,ITPR2,MAPK10,MEF2C,PIK3R1,PIK3R6,PLCB2,PLCE1,PLCG2,PLCL1,PRKCB,PRKCE,PRKCQ,SHC3,SLC8A3,TGFBR2
Apelin Endothelial Signaling Pathway	3.76E+00	1.37E-01	-3.153	ADCY2,ADCY9,ANGPT1,CAMK4,FOS,GNAL,GNAO1,GNG7,JUN,KLF2,MAPK10,MEF2C,PIK3R1,PIK3R6,PLCB2,PRKCB,PRKCE,PRKCQ,TEK
Œ±-Adrenergic Signaling	3.76E+00	1.51E-01	-3.162	ADCY2,ADCY9,ADRA2A,CAMK4,EPM2A,GNAL,GNAO1,GNG7,ITPR1,ITPR2,PHKB,PLCG2,PRKCB,PRKCE,PRKCQ,SLC8A3
Thrombin Signaling	3.76E+00	1.19E-01	-3.578	ADCY2,ADCY9,ARHGEF15,ARHGEF6,ARHGEF9,CAMK2D,CAMK4,GATA5,GATA6,GNAL,GNAO1,GNG7,ITPR1,ITPR2,MYL3,PIK3R1,PIK3R6,PLCB2,PLCE1,PLCG2,PLCL1,PPP1R12B,PRKCB,PRKCE,PRKCQ
Opioid Signaling Pathway	3.67E+00	1.09E-01	-3.657	ADCY2,ADCY9,ARRB2,BLK,CACNA1D,CACNA2D2,CACNA2D3,CACNB4,CAMK2D,CAMK4,FOS,FOSB,GNAL,GNAO1,GNG7,ITPR1,ITPR2,KCNJ5,PDE1B,PDE1C,PENK,PRKCB,PRKCE,PRKCQ,RGS13,RGS5,RGS9,RPS6KA5,RYR2,SCN7A
Nitric Oxide Signaling in the Cardiovascular System	3.66E+00	1.43E-01	-3.606	CACNA1D,CACNA2D2,CACNA2D3,CACNB4,CAMK4,GUCY1A2,ITPR1,ITPR2,PDE1B,PDE1C,PIK3R1,PIK3R6,PRKCB,PRKCE,PRKCQ,RYR2,VEGFD
Granulocyte Adhesion and Diapedesis	3.65E+00	1.22E-01	NaN	CCL14,CCL17,CCL19,CCL22,CCL23,CCR2,CCR4,CCR6,CCR7,CLDN18,CLDN2,CSF3R,CX3CL1,CXCL14,CXCL16,IL33,MMP24,MMP28,MMP9,NGFR,PECAM1,SELL,SELP
Agranulocyte Adhesion and Diapedesis	3.63E+00	1.17E-01	NaN	AOC3,CCL14,CCL17,CCL19,CCL22,CCL23,CCR2,CCR4,CCR6,CCR7,CD34,CLDN18,CLDN2,CX3CL1,CXCL14,CXCL16,IL33,MMP24,MMP28,MMP9,MYH11,MYL3,PECAM1,SELL,SELP
Role of Pattern Recognition Receptors in Recognition of Bacteria and Viru	3.55E+00	1.28E-01	-3.464	C3,CD40LG,IL12B,IL33,LTA,LTB,MAPK10,PIK3R1,PIK3R6,PLCG2,PRKCB,PRKCE,PRKCQ,RNASEL,TLR2,TLR3,TLR5,TLR7,TNFSF12,TNFSF13
GŒ±q Signaling	3.47E+00	1.24E-01	-4.243	BTK,CAMK4,EPM2A,GNAL,GNAO1,GNG7,GPLD1,ITPR1,ITPR2,NFATC1,NFATC2,NFATC3,PIK3R1,PIK3R6,PLCB2,PLCG2,PLD4,PRKCB,PRKCE,PRKCQ,PTK2B
Melatonin Signaling	3.36E+00	1.67E-01	-2.887	CAMK2D,CAMK4,GNAO1,PLCB2,PLCE1,PLCG2,PLCL1,PRKCB,PRKCE,PRKCQ,RORA,RORB
Cardiac Œ≤-adrenergic Signaling	3.33E+00	1.21E-01	-2.333	ADCY2,ADCY9,AKAP13,AKAP14,AKAP6,AKAP7,CACNA1D,CACNA2D2,CACNA2D3,CACNB4,ENPP6,GNAL,GNAO1,GNG7,PDE1B,PDE1C,PDE7B,PDE8B,PPP1R3C,RYR2,SLC8A3
GPCR-Mediated Nutrient Sensing in Enteroendocrine Cells	3.31E+00	1.38E-01	-4	ADCY2,ADCY9,CACNA1D,CACNA2D2,CACNA2D3,CACNB4,GNG7,ITPR1,ITPR2,PLCB2,PLCE1,PLCG2,PLCL1,PRKCB,PRKCE,PRKCQ
Factors Promoting Cardiogenesis in Vertebrates	3.30E+00	1.26E-01	-4.243	BMP3,BMP5,CAMK2D,LRP6,MAPK10,MEF2C,MYOCD,PLCB2,PLCE1,PLCG2,PLCL1,PRKCB,PRKCE,PRKCQ,TBX5,TGFBR2,TGFBR3,WNT11,WNT2B
G Beta Gamma Signaling	3.24E+00	1.32E-01	-3.873	ADCY2,ARHGEF6,BTK,CACNA1D,CACNA2D2,CACNA2D3,CACNB4,GNAL,GNAO1,GNG7,ITPR1,ITPR2,KCNJ5,PLCG2,PRKCB,PRKCE,PRKCQ
FcŒ≥RIIB Signaling in B Lymphocytes	3.22E+00	1.53E-01	-2.646	BLNK,BTK,CACNA1D,CACNA2D2,CACNA2D3,CACNB4,CD79B,ITPR1,ITPR2,MAPK10,PIK3R1,PIK3R6,PLCG2
Gustation Pathway	3.16E+00	1.20E-01	NaN	ADCY2,ADCY9,CACNA1D,CACNA2D2,CACNA2D3,CACNB4,ENPP6,GNG7,ITPR1,ITPR2,P2RX1,P2RY12,P2RY13,P2RY14,P2RY8,PDE1B,PDE1C,PDE7B,PDE8B,PLCB2
Renin-Angiotensin Signaling	3.14E+00	1.33E-01	-3.742	ADCY2,ADCY9,FOS,ITPR1,ITPR2,JUN,MAPK10,PIK3R1,PIK3R6,PLCG2,PRKCB,PRKCE,PRKCQ,PTK2B,SHC3,SHE
Dopamine-DARPP32 Feedback in cAMP Signaling	3.11E+00	1.16E-01	-3.357	ADCY2,ADCY9,CACNA1D,CACNA2D2,CACNA2D3,CACNB4,CAMK4,GUCY1A2,ITPR1,ITPR2,KCNJ15,KCNJ16,KCNJ5,PLCB2,PLCE1,PLCG2,PLCL1,PPP1R3C,PRKCB,PRKCE,PRKCQ
Apelin Cardiomyocyte Signaling Pathway	3.07E+00	1.41E-01	-3.742	CAT,ITPR1,MAPK10,MYL3,PIK3R1,PIK3R6,PLCB2,PLCE1,PLCG2,PLCL1,PRKCB,PRKCE,PRKCQ,SLC8A3
Dilated Cardiomyopathy Signaling Pathway	3.05E+00	1.23E-01	-1.155	ADCY2,ADCY9,BCL2,CACNA1D,CACNA2D2,CACNA2D3,CACNB4,CAMK2D,CAMK4,DES,DMD,ITPR1,ITPR2,MAP3K3,MYH11,MYL3,PRKCE,RYR2
nNOS Signaling in Skeletal Muscle Cells	3.03E+00	1.88E-01	NaN	CACNA1D,CACNA2D2,CACNA2D3,CACNB4,CAMK4,DMD,ITPR1,ITPR2,RYR2
IL-8 Signaling	2.97E+00	1.09E-01	-4.025	ANGPT1,ARRB2,BCL2,CR2,FOS,GNAL,GNAO1,GNG7,GPLD1,JUN,MAPK10,MMP9,PIK3R1,PIK3R6,PLCB2,PLD4,PRKCB,PRKCE,PRKCQ,PTK2B,RAB11FIP2,TEK,VEGFD
Axonal Guidance Signaling	2.89E+00	8.70E-02	NaN	ADAMTS8,ARHGEF15,ARHGEF6,BMP3,BMP5,DPYSL2,EPHA3,GNAL,GNAO1,GNG7,HHIP,ITGA10,ITGA8,ITGA9,ITGAL,MMP24,MMP28,MMP9,MYL3,NFATC1,NFATC2,NFATC3,NGFR,NTNG1,NTRK2,NTRK3,PIK3R1,PIK3R6,PLCB2,PLCE1,PLCG2,PLCL1,PLXNA2,PRKCB,PRKCE,PRKCQ,PTCH1,RASSF5,ROBO2,SLIT2,SLIT3,VEGFD,WNT11,WNT2B
Amyotrophic Lateral Sclerosis Signaling	2.88E+00	1.30E-01	-1.508	BCL2,CACNA1D,CACNA2D2,CACNA2D3,CACNB4,CAPN6,CAT,GRIA1,GRID1,GRIK3,GRIK4,PIK3R1,PIK3R6,SLC1A2,VEGFD
Aldosterone Signaling in Epithelial Cells	2.86E+00	1.16E-01	-3.606	DNAJC27,DUSP1,HSPB6,HSPB7,HSPB8,ITPR1,ITPR2,KCNMB2,NR3C2,PIK3R1,PIK3R6,PIP5K1B,PLCB2,PLCE1,PLCG2,PLCL1,PRKCB,PRKCE,PRKCQ
Growth Hormone Signaling	2.86E+00	1.55E-01	-3.317	A2M,CEBPA,FOS,GHR,PIK3R1,PIK3R6,PLCG2,PRKCB,PRKCE,PRKCQ,RPS6KA5
April Mediated Signaling	2.80E+00	1.90E-01	-2.828	FOS,JUN,MAPK10,NFATC1,NFATC2,NFATC3,TNFRSF13B,TNFSF13

UVB-Induced MAPK Signaling	2.77E+00	1.73E-01	-3	FOS,JUN,MAPK10,PIK3R1,PIK3R6,PRKCB,PRKCE,PRKCQ,RPS6KA5
Coagulation System	2.63E+00	2.00E-01	-0.378	A2M,F10,F11,F8,PLG,SERPIND1,VWF
eNOS Signaling	2.63E+00	1.13E-01	-3.464	ADCY2,ADCY9,AQP1,AQP4,AQP5,CAMK4,CHRNA6,CNGA4,GUCY1A2,ITPR1,ITPR2,PIK3R1,PIK3R6,PLCG2,PRKCB,PRKCE,PRKCQ,VEGFD
GDNF Family Ligand-Receptor Interactions	2.61E+00	1.45E-01	-3	DOK6,FOS,GFRA1,GFRA2,ITPR1,ITPR2,JUN,MAPK10,PIK3R1,PIK3R6,PLCG2
Signaling by Rho Family GTPases	2.55E+00	9.70E-02	-3.9	ARHGEF15,ARHGEF18,ARHGEF6,ARHGEF9,CDH19,CDH23,DES,FOS,GNAL,GNAO1,GNG7,ITGA10,ITGA8,ITGA9,ITGAL,JUN,MAPK10,MYL3,PIK3R1,PIK3R6,PIP5K1B,PPP1R12B,PTK2B,SEPTIN1,SEPTIN4,WASF3
GNRH Signaling	2.53E+00	1.06E-01	-3.873	ADCY2,ADCY9,CACNA1D,CACNA2D2,CACNA2D3,CACNB4,CAMK2D,CAMK4,FOS,GNG7,ITPR1,ITPR2,JUN,MAP3K3,MAPK10,PLCB2,PRKCB,PRKCE,PRKCQ,PTK2B
Colorectal Cancer Metastasis Signaling	2.53E+00	9.67E-02	-4.264	ADCY2,ADCY9,FOS,GNAL,GNAO1,GNG7,IL6R,IL6ST,JUN,LRP6,MAPK10,MMP24,MMP28,MMP9,PIK3R1,PIK3R6,PTGER4,TGFBR2,TLR10,TLR2,TLR3,TLR5,TLR7,VEGFD,WNT11,WNT2B
Cardiac Hypertrophy Signaling	2.47E+00	9.69E-02	-4.472	ADCY2,ADCY9,ADRA2A,ADRB2,CACNA1D,CACNA2D2,CACNA2D3,CACNB4,CAMK4,GNAL,GNAO1,GNG7,IL6R,JUN,MAP3K3,MAPK10,MEF2C,MYL3,PIK3R1,PIK3R6,PLCB2,PLCE1,PLCG2,PLCL1,TGFBR2
Type II Diabetes Mellitus Signaling	2.46E+00	1.12E-01	-2.828	ACSBG1,ACSL5,ACSL6,CACNA1D,CACNA2D2,CACNA2D3,CACNB4,CD36,ITPR1,ITPR2,MAPK10,NGFR,PIK3R1,PIK3R6,PRKCB,PRKCE,PRKCQ
Relaxin Signaling	2.43E+00	1.11E-01	-2.53	ADCY2,ADCY9,ENPP6,FOS,GNAL,GNAO1,GNG7,GUCY1A2,JUN,MMP9,PDE1B,PDE1C,PDE7B,PDE8B,PIK3R1,PIK3R6,RXFP1
Glucocorticoid Receptor Signaling	2.42E+00	8.09E-02	NaN	A2M,ADRB2,AR,BCL2,CEBPA,DNAH10,DNALI1,DUSP1,FBP1,FOS,GHR,HLA-DMA,HLA-DMB,HLA-DOA,HLA-DOB,HLA-DPA1,HLA-DPB1,HLA-DQA1,HLA-DQB1,HLA-DQB2,HLA-DRA,HLA-DRB1,HLA-DRB5,HLA-E,HP,IL13RA2,IL5RA,IL6R,IL6ST,JUN,KAT2B,KRT1,MAPK10,MMP9,NFATC1,NFATC2,NFATC3,NR3C2,PGR,PIK3R1,PIK3R6,RPS6KA5,RXRG,SCGB1A1,SMARCA2,TGFBR2,TLR2
CXCR4 Signaling	2.40E+00	1.08E-01	-2.673	ADCY2,ADCY9,ELMO1,FOS,GNAL,GNAO1,GNG7,ITPR1,ITPR2,JUN,MAPK10,MYL3,PIK3R1,PIK3R6,PLCB2,PRKCB,PRKCE,PRKCQ
P2Y Purigenic Receptor Signaling Pathway	2.39E+00	1.16E-01	-3.357	ADCY2,ADCY9,FOS,GNG7,JUN,P2RY12,PIK3R1,PIK3R6,PLCB2,PLCE1,PLCG2,PLCL1,PRKCB,PRKCE,PRKCQ
ErbB Signaling	2.34E+00	1.28E-01	-3.464	ERBB4,FOS,JUN,MAPK10,NRG1,NRG2,PIK3R1,PIK3R6,PLCG2,PRKCB,PRKCE,PRKCQ
fMLP Signaling in Neutrophils	2.33E+00	1.15E-01	-3.606	CAMK4,GNAL,GNAO1,GNG7,ITPR1,ITPR2,NFATC1,NFATC2,NFATC3,PIK3R1,PIK3R6,PLCB2,PRKCB,PRKCE,PRKCQ
HGF Signaling	2.30E+00	1.14E-01	-3.317	FOS,HGF,ITGA10,ITGA8,ITGA9,ITGAL,JUN,MAP3K3,MAPK10,PIK3R1,PIK3R6,PLCG2,PRKCB,PRKCE,PRKCQ
Netrin Signaling	2.29E+00	1.39E-01	-2.828	CACNA1D,CACNA2D2,CACNA2D3,CACNB4,ITPR1,ITPR2,NFATC1,NFATC2,NFATC3,RYR2
UVA-Induced MAPK Signaling	2.19E+00	1.22E-01	-2.646	FOS,JUN,MAPK10,PARP11,PARP15,PIK3R1,PIK3R6,PLCB2,PLCE1,PLCG2,PLCL1,RPS6KA5
IL-15 Production	2.19E+00	1.14E-01	-3.742	BLK,BMX,BTK,EPHA3,ERBB4,FGFR2,FLT3LG,MUSK,NTRK2,NTRK3,PTK2B,ROS1,TEK,ZAP70
Calcium Signaling	2.18E+00	9.72E-02	-3.3	CACNA1D,CACNA2D2,CACNA2D3,CACNB4,CAMK2D,CAMK4,CASQ1,CASQ2,CHRNA6,GRIA1,ITPR1,ITPR2,MEF2C,MYH11,MYL3,NFATC1,NFATC2,NFATC3,RYR2,SLC8A3,TRPC6
GŒ±i Signaling	2.12E+00	1.09E-01	-2.309	ADCY2,ADCY9,ADRA2A,CCR4,CNR2,GNAL,GNAO1,GNG7,NPY1R,P2RY12,P2RY13,P2RY14,S1PR1,SHC3,XCR1
B Cell Activating Factor Signaling	2.12E+00	1.63E-01	-2.449	FOS,JUN,MAPK10,NFATC1,NFATC2,NFATC3,TNFRSF13B
Regulation Of The Epithelial Mesenchymal Transition By Growth Factors P	2.10E+00	9.90E-02	-3.5	CD40LG,FGF10,FGF14,FGFR2,FOS,HGF,IL6R,JUN,LTA,LTB,MAPK10,MMP9,NGFR,PIK3R1,PIK3R6,SHC3,TGFBR2,TNFSF12,TNFSF13
Human Embryonic Stem Cell Pluripotency	2.08E+00	1.02E-01	NaN	BMP3,BMP5,FGFR2,GNAL,GNAO1,GNG7,LEFTY2,NTRK2,NTRK3,PIK3R1,PIK3R6,S1PR1,S1PR4,SMAD6,TGFBR2,WNT11,WNT2B
4-aminobutyrate Degradation I	2.08E+00	6.67E-01	NaN	ABAT,ALDH5A1
GP6 Signaling Pathway	2.07E+00	1.10E-01	-3.606	BTK,CAMK4,COL6A5,COL6A6,GRAP2,ITPR1,LAMA2,PIK3R1,PIK3R6,PLCG2,PRKCB,PRKCE,PRKCQ,RASGRP2
Synaptic Long Term Depression	2.05E+00	9.79E-02	-4.359	CACNA1D,CACNA2D2,CACNA2D3,CACNB4,GNAL,GNAO1,GRIA1,GRID1,GUCY1A2,ITPR1,ITPR2,PLCB2,PLCE1,PLCG2,PLCL1,PRKCB,PRKCE,PRKCQ,RYR2
B Cell Receptor Signaling	2.05E+00	9.79E-02	-4.359	BLNK,BTK,CAMK2D,CAMK4,CD22,CD79B,JUN,MAP3K3,MEF2C,NFATC1,NFATC2,NFATC3,PIK3R1,PIK3R6,PLCG2,PRKCB,PRKCQ,PTK2B,RASSF5
Inhibition of Angiogenesis by TSP1	2.05E+00	1.76E-01	-2.236	CD36,GUCY1A2,JUN,MAPK10,MMP9,TGFBR2
Role of NFAT in Regulation of the Immune Response	2.00E+00	7.76E-02	-5.385	BLNK,BTK,CAMK4,CD79B,FCER1A,FOS,GNAL,GNAO1,GNG7,HLA-DMA,HLA-DMB,HLA-DOA,HLA-DOB,HLA-DPA1,HLA-DPB1,HLA-DQA1,HLA-DQB1,HLA-DQB2,HLA-DRA,HLA-DRB1,HLA-DRB5,ITPR1,ITPR2,JUN,KPNA5,MEF2C,MS4A2,NFATC1,NFATC2,NFATC3,PIK3R1,PIK3R6,PLCB2,PLCG2,PRKCQ,TRAV8-2,TRAV8-4,TRAV8-6,TRAV9-2,TRBV19,TRBV29-1,TRBV5-1,TRBV6-1,ZAP70
ErbB4 Signaling	1.98E+00	1.32E-01	-3	ERBB4,NRG1,NRG2,PIK3R1,PIK3R6,PLCG2,PRKCB,PRKCE,PRKCQ
Airway Pathology in Chronic Obstructive Pulmonary Disease	1.95E+00	1.10E-01	NaN	CD40LG,CTSG,FGF10,FGF14,IL12B,IL33,LTA,LTB,MMP9,PTGDS,TNFSF12,TNFSF13,TSLP
GABA Receptor Signaling	1.95E+00	1.07E-01	NaN	ABAT,ADCY2,ADCY9,ALDH5A1,CACNA1D,CACNA2D2,CACNA2D3,CACNB4,GNAL,GNAO1,GNG7,ITPR1,ITPR2,KCNN3
Acute Phase Response Signaling	1.95E+00	9.73E-02	-2.887	A2M,C3,C4A/C4B,C4BPA,F8,FOS,HP,IL33,IL6R,IL6ST,ITIH3,JUN,NGFR,PIK3R1,PLG,RBP5,SERPIND1,VWF
Agrin Interactions at Neuromuscular Junction	1.90E+00	1.29E-01	-2.646	ARHGEF6,ERBB4,JUN,LAMA2,MAPK10,MUSK,NRG1,NRG2,UTRN
RhoGDI Signaling	1.90E+00	9.30E-02	2.309	ARHGAP6,ARHGDIB,ARHGEF15,ARHGEF18,ARHGEF6,ARHGEF9,CDH19,CDH23,DLC1,GNAL,GNAO1,GNG7,ITGA10,ITGA8,ITGA9,ITGAL,MYH11,MYL3,PIP5K1B,PPP1R12B
Hepatic Cholestasis	1.86E+00	9.52E-02	NaN	ADCY2,ADCY9,CD40LG,CYP27A1,IL12B,IL33,JUN,LTA,LTB,MAPK10,NGFR,NR0B2,PRKCB,PRKCE,PRKCQ,SLCO3A1,TNFSF12,TNFSF13
STAT3 Pathway	1.84E+00	1.04E-01	-3.162	BCL2,FGFR2,GHR,HGF,IL13RA2,IL5RA,IL6R,IL6ST,MAPK10,NGFR,NTRK2,NTRK3,TGFBR2,TGFBR3
White Adipose Tissue Browning Pathway	1.82E+00	1.03E-01	-2.887	ADCY2,ADCY9,CACNA1D,CACNA2D2,CACNA2D3,CACNB4,FCER1A,FGFR2,GUCY1A2,ITPR1,ITPR2,MS4A2,NDN,RXRG
Erythropoietin Signaling Pathway	1.82E+00	9.60E-02	-0.5	CD40LG,FOS,IL12B,IL33,ITPR1,ITPR2,JUN,LTA,LTB,PIK3R1,PIK3R6,PRKCB,PRKCE,PRKCQ,SHC3,TNFSF12,TNFSF13

Dendritic Cell Maturation	1.81E+00	7.58E-02	-5.916	CCR7,CD1A,CD1B,CD1C,CD40LG,CD83,HLA-DMA,HLA-DMB,HLA-DOA,HLA-DOB,HLA-DPA1,HLA-DPB1,HLA-DQA1,HLA-DQB1,HLA-DQB2,HLA-DRA,HLA-DRB1,HLA-DRB5,HLA-E,IL12B,IL33,IRF8,LTA,LTB,MAPK10,MR1,NGFR,PIK3R1,PIK3R6,PLCB2,PLCE1,PLCG2,PLCL1,TLR2,TLR3,TRAV8-2,TRAV8-4,TRAV8-6,TRAV9-2,TRBV19,TRBV29-1,TRBV5-1,TRBV6-1
PTEN Signaling	1.81E+00	1.00E-01	3.162	BCL2,FGFR2,GHR,ITGA10,ITGA8,ITGA9,ITGAL,MAGI3,NGFR,NTRK2,NTRK3,PIK3R1,PREX2,TGFBR2,TGFBR3
GPCR-Mediated Integration of Enteroendocrine Signaling Exemplified by a	1.79E+00	1.23E-01	-0.333	ADCY2,ADCY9,ADRB2,ITPR1,ITPR2,PLCB2,PLCE1,PLCG2,PLCL1
Molecular Mechanisms of Cancer	1.78E+00	7.87E-02	NaN	ADCY2,ADCY9,ARHGEF15,ARHGEF18,ARHGEF6,ARHGEF9,BCL2,BMP3,BMP5,CAMK2D,FOS,GNAL,GNAO1,GNG7,ITGA10,ITGA8,ITGA9,ITGAL,JUN,LRP6,MAPK10,PIK3R1,PIK3R6,PLCB2,PRKCB,PRKCE,PRKCQ,PTCH1,RASGRF1,RBL2,SHC3,SMAD6,TGFBR2,WNT11,WNT2B
Leptin Signaling in Obesity	1.75E+00	1.22E-01	NaN	ADCY2,ADCY9,GHRL,PIK3R1,PIK3R6,PLCB2,PLCE1,PLCG2,PLCL1
Role of Osteoblasts, Osteoclasts and Chondrocytes in Rheumatoid Arthritis	1.72E+00	8.93E-02	NaN	BCL2,BMP3,BMP5,CAMK4,FOS,FRZB,IL33,JUN,LRP6,MAPK10,NFATC1,NFATC2,NFATC3,NGFR,PIK3R1,PIK3R6,PTK2B,SMAD6,WNT11,WNT2B
Thrombopoietin Signaling	1.71E+00	1.27E-01	-2.828	FOS,JUN,PIK3R1,PIK3R6,PLCG2,PRKCB,PRKCE,PRKCQ
Androgen Signaling	1.69E+00	9.47E-02	-3.317	AR,CACNA1D,CACNA2D2,CACNA2D3,CACNB4,CAMK4,GNAL,GNAO1,GNG7,ITPR1,ITPR2,JUN,KAT2B,PRKCB,PRKCE,PRKCQ
Synaptic Long Term Potentiation	1.66E+00	1.01E-01	-2.887	CAMK2D,CAMK4,GRIA1,ITPR1,ITPR2,PLCB2,PLCE1,PLCG2,PLCL1,PPP1R3C,PRKCB,PRKCE,PRKCQ
TREM1 Signaling	1.65E+00	1.17E-01	-3	CD83,CIITA,NLRC3,PLCG2,TLR10,TLR2,TLR3,TLR5,TLR7
Neuregulin Signaling	1.62E+00	1.03E-01	-2.828	ERBB4,ITGA10,ITGA8,ITGA9,ITGAL,NRG1,NRG2,PIK3R1,PLCG2,PRKCB,PRKCE,PRKCQ
Toll-like Receptor Signaling	1.62E+00	1.15E-01	-2.449	FOS,IL12B,IL33,JUN,TLR10,TLR2,TLR3,TLR5,TLR7
RANK Signaling in Osteoclasts	1.61E+00	1.10E-01	-3	CAMK4,FOS,JUN,MAP3K3,MAPK10,NFATC1,NFATC2,PIK3R1,PIK3R6,PTK2B
IL-17 Signaling	1.61E+00	9.09E-02	-3.638	CCL17,CCL22,CD40LG,FOS,IL12B,IL33,JUN,LTA,LTB,MAPK10,MMP9,PIK3R1,PIK3R6,RGS13,TNFSF12,TNFSF13,VEGFD
Nicotine Degradation II	1.60E+00	1.21E-01	-2.828	B4GAT1,CYP2U1,CYP4X1,FMO2,FMO3,FMO4,FMO5,INMT
Sphingosine-1-phosphate Signaling	1.59E+00	1.02E-01	-1.732	ADCY2,ADCY9,CASQ1,PIK3R1,PIK3R6,PLCB2,PLCE1,PLCG2,PLCL1,PTK2B,S1PR1,S1PR4
Glutamate Degradation III (via 4-aminobutyrate)	1.59E+00	4.00E-01	NaN	ABAT,ALDH5A1
Cholecystokinin/Gastrin-mediated Signaling	1.57E+00	1.01E-01	-3.464	FOS,IL33,ITPR1,ITPR2,JUN,MAPK10,MEF2C,PLCB2,PRKCB,PRKCE,PRKCQ,PTK2B
CD40 Signaling	1.57E+00	1.19E-01	-2.646	CD40LG,FCER2,FOS,JUN,LTA,MAPK10,PIK3R1,PIK3R6
Chemokine Signaling	1.55E+00	1.13E-01	-2.333	CAMK2D,CAMK4,FOS,JUN,PLCB2,PLCG2,PPP1R12B,PRKCB,PTK2B
G Protein Signaling Mediated by Tubby	1.53E+00	1.36E-01	NaN	GNAL,GNAO1,GNG7,PLCB2,PLCG2,TUB
CCR3 Signaling in Eosinophils	1.51E+00	9.63E-02	-2.449	CAMK4,GNAL,GNAO1,GNG7,ITPR1,ITPR2,PIK3R1,PIK3R6,PLCB2,PPP1R12B,PRKCB,PRKCE,PRKCQ
Estrogen Receptor Signaling	1.51E+00	7.67E-02	-4.536	ADCY2,ADCY9,BCL2,CACNA1D,CACNA2D2,CACNA2D3,CACNB4,FOS,GNAL,GNAO1,GNG7,JUN,MMP24,MMP28,MMP9,MYL3,NR0B2,PGR,PIK3R1,PIK3R6,PLCB2,PLCE1,PLCG2,PLCL1,PPP1R12B,PRKCB,PRKCE,PRKCQ,SHC3,SHE,VEGFD
Senescence Pathway	1.50E+00	8.08E-02	-3.71	CACNA1D,CACNA2D2,CACNA2D3,CACNB4,CAMK4,CAPN6,CAT,CBX7,ITPR2,JUN,KAT2B,NFATC1,NFATC2,NFATC3,PIK3R1,PIK3R6,RASSF5,RBL2,RPS6KA5,SMAD6,STING1,TGFBR2,TGFBR3,TLR2
LPS/IL-1 Mediated Inhibition of RXR Function	1.49E+00	8.33E-02	-1.342	ACSBG1,ACSL5,ACSL6,ALDH5A1,CAT,CES2,CHST7,CYP2U1,FABP4,FMO2,FMO3,FMO4,FMO5,GSTM5,IL33,JUN,MAOB,NGFR,NR0B2,SULT1C4,UST
IL-12 Signaling and Production in Macrophages	1.49E+00	9.56E-02	NaN	ALOX15,CD40LG,FOS,IL12B,IRF8,JUN,MAPK10,PIK3R1,PIK3R6,PRKCB,PRKCE,PRKCQ,TLR2
Insulin Secretion Signaling Pathway	1.48E+00	8.21E-02	-3.9	ADCY2,ADCY9,CACNA1D,CACNA2D2,CACNA2D3,CACNB4,CAMK2D,GHR,ITPR1,ITPR2,KCNB1,PIK3R1,PIK3R6,PLCB2,PLCE1,PLCG2,PLCL1,PRKCB,PRKCE,PRKCQ,RPS6KA5,RYR2
PKCŒ∏ Signaling in T Lymphocytes	1.44E+00	7.28E-02	-4.69	CACNA1D,CACNA2D2,CACNA2D3,CACNB4,CAMK2D,FOS,GRAP2,HLA-DMA,HLA-DMB,HLA-DOA,HLA-DOB,HLA-DPA1,HLA-DPB1,HLA-DQA1,HLA-DQB1,HLA-DQB2,HLA-DRA,HLA-DRB1,HLA-DRB5,ITPR1,ITPR2,JUN,MAP3K3,NFATC1,NFATC2,NFATC3,PIK3R1,PIK3R6,PLCG2,PRKCQ,TRAV8-2,TRAV8-4,TRAV8-6,TRAV9-2,TRBV19,TRBV29-1,TRBV5-1,TRBV6-1,ZAP70
Fatty Acid Activation	1.44E+00	2.14E-01	NaN	ACSBG1,ACSL5,ACSL6
RAR Activation	1.44E+00	8.67E-02	NaN	ADCY2,ADCY9,ADH1B,DUSP1,FOS,JUN,KAT2B,MAPK10,NRIP2,PIK3R1,PRKCB,PRKCE,PRKCQ,RBP5,RXRG,SMAD6,SMARCA2
HMGB1 Signaling	1.44E+00	8.98E-02	-3.162	AGER,CD40LG,FOS,IL12B,IL33,JUN,KAT2B,LTA,LTB,MAPK10,NGFR,PIK3R1,PIK3R6,TNFSF12,TNFSF13
14-3-3-mediated Signaling	1.38E+00	9.45E-02	-3.464	FOS,JUN,MAPK10,PIK3R1,PIK3R6,PLCB2,PLCE1,PLCG2,PLCL1,PRKCB,PRKCE,PRKCQ
Prolactin Signaling	1.38E+00	1.05E-01	-2.828	FOS,JUN,KCNMB2,PIK3R1,PIK3R6,PLCG2,PRKCB,PRKCE,PRKCQ
D-myo-inositol (1,4,5)-Trisphosphate Biosynthesis	1.31E+00	1.54E-01	-2	PIP5K1B,PLCB2,PLCE1,PLCG2

Supplemental Table 3. Pathway Analysis for Differential Gene Expression Heatmap Cluster 3. Pathways involved in the differential expression of genes in heatmap cluster 3 are shown. Ratio refers to the number of differentially regulated genes in the dataset relative to the total number of pathway genes. Z-score refers to the relationship between observed vs. predicted pathway member regulation. A positive Z-score indicates that the observed gene activity positively correlates with predicted pathway member up/down regulation patterns; a negative Z-score indicates anti-correlation.

Table S4 - Perturbed Biological Pathways in Recurrence High- vs. Low-Risk Stage I Lung Adenocarcinomas by Topology-Based Enrichment Analysis

Pathway	pSize	NDE	pNDE	tA	pPERT	pG	pGFdr	pGFWER	Status
Vascular smooth muscle contraction	126	111	1.24E-31	-58.38827702	5.00E-06	5.23E-35	1.94E-34	1.07E-32	Inhibited
MicroRNAs in cancer	230	146	3.25E-15	-45.29966781	5.00E-06	7.58E-19	1.24E-18	1.55E-16	Inhibited
Pathways in cancer	470	422	2.14E-123	79.11631369	0.001	6.21E-124	1.27E-121	1.27E-121	Activated
Human cytomegalovirus infection	217	194	1.25E-56	88.78023538	0.001	1.71E-57	2.91E-56	3.49E-55	Activated
Tight junction	169	149	4.36E-42	-42.60929075	0.001	4.50E-43	3.06E-42	9.18E-41	Inhibited
Central carbon metabolism in cancer	64	58	1.57E-18	43.23785372	0.001	7.66E-20	1.28E-19	1.56E-17	Activated
Dilated cardiomyopathy (DCM)	78	66	2.31E-17	-28.23172681	0.001	1.07E-18	1.73E-18	2.18E-16	Inhibited
Bile secretion	30	22	8.64E-05	-28.10858297	0.001	1.49E-06	1.65E-06	0.000304426	Inhibited
Regulation of actin cytoskeleton	214	188	3.36E-52	62.77732596	0.002	8.45E-53	1.08E-51	1.72E-50	Activated
Cellular senescence	147	139	2.02E-48	-51.5404267	0.002	4.73E-49	4.82E-48	9.65E-47	Inhibited
Cytokine-cytokine receptor interaction	283	218	3.42E-41	-21.53624554	0.002	6.88E-42	4.13E-41	1.40E-39	Inhibited
Shigellosis	223	206	3.39E-66	43.45722655	0.003	1.60E-66	3.64E-65	3.27E-64	Activated
Ras signaling pathway	228	205	8.99E-61	-87.87430295	0.003	3.91E-61	7.26E-60	7.98E-59	Inhibited
Ovarian steroidogenesis	41	32	1.85E-07	-29.36515716	0.003	1.24E-08	1.45E-08	2.53E-06	Inhibited
Bacterial invasion of epithelial cells	52	50	4.25E-19	39.19924354	0.004	8.30E-20	1.38E-19	1.69E-17	Activated
GABAergic synapse	66	51	7.96E-11	18.4672492	0.004	9.48E-12	1.28E-11	1.93E-09	Activated
Small cell lung cancer	91	91	3.89E-39	24.7215063	0.005	1.84E-39	9.89E-39	3.76E-37	Activated
Amphetamine addiction	63	53	5.44E-14	-18.0023855	0.006	1.20E-14	1.73E-14	2.44E-12	Inhibited
Toll-like receptor signaling pathway	102	85	4.55E-21	59.34678208	0.007	1.68E-21	3.09E-21	3.43E-19	Activated
Alzheimer disease	240	220	5.57E-69	-53.46304293	0.008	7.27E-69	2.12E-67	1.48E-66	Inhibited
Estrogen signaling pathway	126	100	1.99E-21	114.0503422	0.008	8.53E-22	1.60E-21	1.74E-19	Activated
Kaposi sarcoma-associated herpesvirus infection	162	141	1.83E-38	56.48316212	0.009	1.53E-38	7.61E-38	3.12E-36	Activated
Neuroactive ligand-receptor interaction	189	91	0.002513082	-13.55541212	0.01	0.000291302	0.000306317	0.059425561	Inhibited
Human T-cell leukemia virus 1 infection	182	176	1.15E-65	-33.6308446	0.014	2.49E-65	5.08E-64	5.08E-63	Inhibited
Fanconi anemia pathway	40	38	2.92E-14	6.930176609	0.017	1.80E-14	2.59E-14	3.67E-12	Activated
Ferroptosis	13	12	7.38E-05	-5.831261782	0.017	1.83E-05	2.01E-05	0.003733879	Inhibited
Legionellosis	41	41	5.10E-18	17.86505779	0.02	4.56E-18	7.21E-18	9.30E-16	Activated
Hepatitis C	128	112	2.66E-31	36.65526896	0.022	4.40E-31	1.23E-30	8.97E-29	Activated
Tuberculosis	170	151	1.49E-43	41.14496513	0.024	3.69E-43	2.69E-42	7.54E-41	Activated
Systemic lupus erythematosus	18	18	2.57E-08	-10.49341308	0.025	1.43E-08	1.66E-08	2.91E-06	Inhibited
PPAR signaling pathway	69	57	3.01E-14	-12.59719624	0.026	2.80E-14	4.00E-14	5.72E-12	Inhibited
FoxO signaling pathway	123	114	1.42E-37	30.54527262	0.032	4.06E-37	1.88E-36	8.27E-35	Activated
Sphingolipid signaling pathway	96	94	3.75E-37	30.69862354	0.032	1.06E-36	4.70E-36	2.16E-34	Activated
Hedgehog signaling pathway	49	47	6.97E-18	4.954323919	0.032	9.81E-18	1.52E-17	2.00E-15	Activated
Complement and coagulation cascades	55	48	4.51E-14	7.661853258	0.035	5.53E-14	7.79E-14	1.13E-11	Activated
Epstein-Barr virus infection	162	145	8.22E-43	92.81298127	0.038	3.16E-42	1.95E-41	6.45E-40	Activated
Intestinal immune network for IgA production	27	25	4.08E-09	-4.4799927	0.038	3.66E-09	4.36E-09	7.46E-07	Inhibited
Cytosolic DNA-sensing pathway	43	29	8.16E-05	11.48007474	0.04	4.45E-05	4.83E-05	0.009073866	Activated
Salivary secretion	48	41	1.49E-11	-11.42482948	0.041	1.78E-11	2.35E-11	3.64E-09	Inhibited
Salmonella infection	201	193	3.46E-70	64.34851789	0.047	2.67E-69	9.07E-68	5.44E-67	Activated
Adrenergic signaling in cardiomyocytes	149	130	8.13E-36	-23.48307348	0.048	3.31E-35	1.30E-34	6.75E-33	Inhibited

Supplemental Table 4. Perturbed Biological Pathways in Recurrence High- vs. Low-Risk Stage I Lung Adenocarcinomas by Topology-Based Enrichment Analysis. Perturbed pathways identified by topology-based enrichment analysis of differentially expressed genes between recurrence high- and low-risk tumors are shown. pSize refers to the number of genes on the pathway; NDE refers to the number of differentially expressed genes per pathway; tA is the observed pathway total perturbation accumulation; pNDE refers to the probability that the number of differentially expressed genes in each pathway is observed by chance using a hypergeometric model; pPERT is the probability that total accumulation more extreme than tA is observed by chance; pG refers to the p-value obtained by combining pNDE and pPERT; pGFdr and pGFWER are the false discovery rate and Bonferroni adjusted global p-values respectively; Status describes the direction in which the pathway is perturbed (activated or inhibited).

Table S5. Pathways Represented by Differentially Methylated Genes in Recurrence High- vs. Low-Risk Stage I Lung Adenocarcinomas

GO	ONTOLOGY	Description	N	DE	P.DE	FDR
GO:0030883	MF	endogenous lipid antigen binding	5	4	2.30E-06	1.74E-02
GO:0030884	MF	exogenous lipid antigen binding	5	4	2.30E-06	1.74E-02
GO:0048006	BP	antigen processing and presentation, endogenous lipid antigen via MHC class Ib	5	4	2.30E-06	1.74E-02
GO:0030882	MF	lipid antigen binding	6	4	7.61E-06	4.32E-02
GO:0030029	BP	actin filament-based process	795	87	4.12E-05	1.87E-01
GO:0071723	MF	lipopeptide binding	10	4	7.61E-05	2.88E-01
GO:0007154	BP	cell communication	6312	427	1.41E-04	3.59E-01
GO:0050832	BP	defense response to fungus	36	7	1.51E-04	3.59E-01
GO:0005886	CC	plasma membrane	5112	349	1.81E-04	3.59E-01
GO:0098590	CC	plasma membrane region	1224	113	2.09E-04	3.59E-01

KEGG	Description	N	DE	P.DE	FDR
path:hsa05146	Amoebiasis	100	18	2.56E-05	0.008719027
path:hsa05131	Shigellosis	239	29	2.08E-04	0.035444957
path:hsa04510	Focal adhesion	200	28	1.30E-03	0.114595143
path:hsa04976	Bile secretion	81	12	0.001513946	0.114595143
path:hsa05230	Central carbon metabolism in cancer	70	13	0.001680281	0.114595143
path:hsa05135	Yersinia infection	134	17	0.004363642	0.185250164
path:hsa04911	Insulin secretion	86	14	0.005282931	0.185250164
path:hsa04918	Thyroid hormone synthesis	75	12	0.005392863	0.185250164
path:hsa04930	Type II diabetes mellitus	46	10	0.005471343	0.185250164
path:hsa04931	Insulin resistance	108	15	0.00558973	0.185250164

MSigDB HALLMARK	Description	N	DE	P.DE	FDR
	HALLMARK_ALLOGRAFT_REJECTION	199	39	0.001634497	0.039865048
	HALLMARK_KRAS_SIGNALING_DN	198	42	0.001937179	0.039865048
	HALLMARK_MYOGENESIS	200	47	0.002391903	0.039865048
	HALLMARK_ESTROGEN_RESPONSE_LATE	200	38	0.026081956	0.326024447
	HALLMARK_INFLAMMATORY_RESPONSE	199	31	0.03455946	0.345594598
	HALLMARK_PANCREAS_BETA_CELLS	40	9	0.066437634	0.553646949
	HALLMARK_TNFA_SIGNALING_VIA_NFKB	200	33	0.110477721	0.78912658
	HALLMARK_ESTROGEN_RESPONSE_EARLY	198	37	0.14697513	0.918594562
	HALLMARK_SPERMATOGENESIS	135	19	0.238967512	0.999976282
	HALLMARK_REACTIVE_OXYGEN_SPECIES_PATHWAY	49	8	0.267207436	0.999976282

Supplemental Table 5. Pathways Represented by Differentially Methylated Genes in Recurrence High- vs. Low-Risk Stage I Lung Adenocarcinomas. Pathways involved in the differential methylation of genes between recurrence high- and low-risk tumors identified by GO Ontology, KEGG pathway, and GSEA Molecular Signatures Database analysis are shown

Table S6. Immune Populations and Coefficients in Immune Profile Classifier	
**Immune Population**	**Coefficient**
M1 Macrophages	-0.006568133
Classical Monocytes	0.088004255
B-cells	-0.007410987
Mast Cells	-0.066172467
Naïve T-cells	0.018646621
Exhausted cytotoxic T-cells	0.010821462

Supplemental Table 6. Immune Populations and Coefficients in Immune Profile Classifier. Elastic net penalized regression analysis was used to assign coefficients to each immune population based on that population's contribution to patient prognosis. An immune profile score was generated for each tumor sample by summing the product of the immune population density and the cox proportional hazards model coefficient for each immune cell population. Immune cell populations with coefficients of zero were not included in the model and are not shown.

Table S7. Summary of Patient Clinical and Pathological Characteristics

	All Stages	Stage I
Number of Patients	500	268
Median Age at Resection	66	67
Females	270 (54%)	158 (59%)
Smoking History		
Yes	414 (83%)	227 (85%)
No	72 (14%)	37 (14%)
Unknown	14 (3%)	4 (2%)
Recurrence at 3 years	177 (35%)	67 (25%)
Pathologic Stage		
I	268 (54%)	268 (100%)
Ia	130 (26%)	130 (49%)
Ib	133 (27%)	133 (50%)
not specified	5 (1%)	5 (2%)
II	120 (24%)	0
IIa	50 (10%)	0
IIb	69 (14%)	0
not specified	1 (<1%)	0
III	79 (16%)	0
IIIa	69 (14%)	0
IIIb	10 (2%)	0
IV	25 (5%)	0
undetermined	8 (2%)	0

Numbers in parentheses represent precentage of total cohort

Supplemental Table 7. Summary of Patient Clinical and Pathological Characteristics. The clinicopathological characteristics of the entire patient cohort (n=500) and stage I patient cohort (n=268) investigated in this study are shown.
